# A Positive Feedback Loop of lncRNA MIR31HG-miR-361-3p -YY1 Accelerates Colorectal Cancer Progression Through Modulating Proliferation, Angiogenesis, and Glycolysis

**DOI:** 10.3389/fonc.2021.684984

**Published:** 2021-08-17

**Authors:** Tao Guo, Defeng Liu, Shihao Peng, Meng Wang, Yangyang Li

**Affiliations:** Department of General Surgery, The Fourth Affiliated Hospital of Anhui Medical University, Hefei, China

**Keywords:** MIR31HG, miR-361-3p, YY1 transcription factor, colorectal cancer, proliferation, angiogenesis, glycolysis

## Abstract

**Background:**

Colorectal cancer (CRC) is a common malignant tumor with high metastatic and recurrent rates. This study probes the effect and mechanism of long non-coding RNA MIR31HG on the progression of CRC cells.

**Materials and Methods:**

Quantitative real-time PCR (qRT-PCR) was used to analyze the expression of MIR31HG and miR-361-3p in CRC tissues and normal tissues. Gain- or loss-of-function assays were conducted to examine the roles of MIR31HG, miR-361-3p and YY1 transcription factor (YY1) in the CRC progression. 3-(4,5-Dimethylthiazol-2-yl)-2,5-diphenyltetrazolium bromide (MTT) assay, and colony formation experiment were conducted to test CRC cell proliferation. CRC cell invasion was determined by Transwell assay. The glucose detection kit and lactic acid detection kit were utilized to monitor the levels of glucose and lactate in CRC cells. The glycolysis level in CRC cells was examined by the glycolytic stress experiment. Western blot was performed to compare the expression of glycolysis-related proteins (PKM2, GLUT1 and HK2) and angiogenesis-related proteins (including VEGFA, ANGPT1, HIF1A and TIMP1) in HUVECs. The binding relationships between MIR31HG and miR-361-3p, miR-361-3p and YY1 were evaluated by the dual-luciferase reporter assay and RNA immunoprecipitation (RIP).

**Results:**

MIR31HG was up-regulated in CRC tissues and was associated with poorer prognosis of CRC patients. The *in-vitro* and *in-vivo* experiments confirmed that overexpressing MIR31HG heightened the proliferation, growth, invasion, glycolysis and lung metastasis of CRC cells as well as the angiogenesis of HUVECs. In addition, MIR3HG overexpression promoted YY1 mRNA and protein level, and forced overexpression of YY1 enhanced MIR31HG level. Overexpressing YY1 reversed the tumor-suppressive effect mediated by MIR31HG knockdown. miR-361-3p, which was inhibited by MIR31HG overexpression, repressed the malignant behaviors of CRC cells. miR-361-3p-mediated anti-tumor effects were mostly reversed by upregulating MIR31HG. Further mechanism studies illustrated that miR-361-3p targeted and negatively regulated the expression of YY1.

**Conclusion:**

This study reveals that MIR31HG functions as an oncogenic gene in CRC *via* forming a positive feedback loop of MIR31HG-miR-361-3p-YY1.

## Introduction

Colorectal cancer (CRC) is among the most frequent and lethal tumors in the world, and its occurrence and development are closely related to genetic factors, dietary habits, and inflammation (1). Besides, the mortality and morbidity of CRC are high due to the inconspicuous early symptoms and high metastatic rate (2, 3). Traditional therapies including surgery, chemotherapy, and radiotherapy have improved the survival of CRC patients. Moreover, targeted therapies, such as BRAF mutation-targeted therapies, EGFR-targeted therapy, immunotherapy, repressing angiogenesis, also bring hope for curing CRC (4). Therefore, understanding the biological mechanisms involved in CRC proliferation, angiogenesis and glycolysis contributes to CRC therapy.

Long noncoding RNAs (lncRNAs) are over 200 nucleotides in length (5). A number of studies have illustrated that lncRNAs exert a crucial role in tumor development by acting as oncogenes or tumor suppressors (6). Multiple biological functions are modulated by the altered lncRNAs in tumors. For example, it has been found that lncRNA DANCR promotes lung cancer cell proliferation and growth by down-regulating miR-216a (7). LncRNA RNA associated with metastasis-11 (RAMS11) has higher expression in CRC patients. RAMS11 promotes the proliferation, migration, metastasis, and inhibits apoptosis, and autophagy of CRC cells (8). The lack of oxygen and nutrients limit cancer growth and metastasis during the progression of cancers. In that process, novel capillary blood vessels growth from pre-existing vasculature, also known as angiogenesis, tremendously contributes to tumor development (9). Additionally, aerobic glycolysis is an important metabolic process in tumor cells, which is conducive to tumor cell survival and proliferation (10). Interestingly, lncRNAs have been found with potent effects on angiogenesis and glycolysis. For instance, Zhang Q et al. found that lncRNA NR2F1-AS1 induces angiogenesis *via* activating IGF-1/IGF-1R/ERK pathway (11). lncRNA GLCC1 is overexpressed in CRC cells, and promotes cell survival and proliferation by enhancing glycolysis (12). Notably, it has been found that low expression of MIR31HG was associated with the dismal overall survival, disease-free survival, high tumor stage and lymph node metastasis among patients with digestive system cancers (13). In another study, MIR31HG functions as a predictor of CRC prognosis and potentially regulates intrinsic invasive and/or immuno-evasive capabilities (14). These findings suggest that MIR31HG might be an oncogenic gene in CRC and exerts a carcinogenic effect.

MicroRNAs (miRNAs) are small noncoding RNAs that modulate gene expression by recognizing homologous sequences and interfering with transcription, translation or epigenetic processes, thereby regulating the growth and development of cells (15). Since the discovery of the first miRNA more than 20 years ago, miRNAs have attracted extensive attention in biology. Emerging studies have demonstrated that miRNAs contribute to body development and diseases (especially in cancers), making them effective tools and targets for developing new therapies (16). Similar to lncRNAs, several miRNAs are found to affect the angiogenesis and glycolysis of tumors (17). For example, circSEMA5A accelerates the proliferation, migration, invasion, angiogenesis and glycolysis of breast cancer cells *via* inducing downregulation of miR-330-5p (18). What’s more, the regulatory role of miR-361-3p in tumors has been confirmed. For example, miR-361-3p is significantly up-regulated in liver tumor-initiating cells (T-ICs) which involves in regulating the malignant behaviors of hepatocellular carcinoma (HCC) cells, and miR-361-3p promotes the self-renewal and tumorigenicity of liver T-ICs (19). Importantly, miR-361-3p is confirmed to be significantly down-regulated in CRC and negatively regulated by BBOX1-AS1 (20), indicating that miR-361-3p serves as a tumor suppressor gene in CRC. Nevertheless, it is not clear whether the role of miR-361-3p in CRC is mediated by MIR31HG.

It has been reported that the transcription factor Yin Yang 1 (YY1) can form a dimer and promote the interaction between the activity enhancer and the proximal promoter element, which is a common feature of mammalian gene regulation (21). Several studies have shown that YY1 regulates biological functions of tumor cells by interacting with miRNAs or lncRNAs. For example, miR-142-5p regulates the expression of epithelial-mesenchymal transition (EMT)-related proteins by targeting YY1, thereby dampening lung cancer metastasis (22). Besides, YY1-induced ARAP1-AS1 up-regulation facilitates CRC cell migration, invasion and EMT *via* the Wnt/β-catenin axis (23). However, it is not clear whether the role of YY1 in CRC is affected by miR-361-3p and MIR31HG.

Overall, this article intends to probe the effect of MIR31HG on CRC and its possible mechanism. We tested cell proliferation, angiogenesis, and glycolysis by designing a series of experiments. Our results revealed that MIR31HG was overexpressed in CRC tissues, and overexpressing MIR31HG enhanced CRC cell proliferation and glycolysis as well as endothelial cell angiogenesis. The mechanism study showed that MIR31HG exerted a carcinogenic effect by inhibiting miR-361-3p and boosting YY1, providing a reference for clinical research and intervention treatment of CRC.

## Materials and Methods

### Collection and Treatment of Clinical Specimens

From January to October 2015 to October 2016, the cancerous tissues from 35 CRC patients and paired 35 normal adjacent colorectal tissues were collected in the Fourth Affiliated Hospital of Anhui Medical University. None of the 35 patients received preoperative chemotherapy or radiotherapy, and all of them were identified as CRC by two pathologists with deputy senior titles or above. The normal All tissue specimens were stored in liquid nitrogen at -196°C for later use. The normal colorectal tissues were about 3 cm away from the tumor margins. No tumor cells were found in the normal adjacent colorectal tissues. This study was conducted in accordance with Declaration of Helsinki. The Medical Ethics Committee of The Fourth Affiliated Hospital of Anhui Medical University approved this study, and all the patients involved signed their informed consent before the surgery. The clinical information of all the involved patients were included in **Table 1**.

**Table 1:** The clinical characteristics of the 35 CRC patients.

MIR31HG expression		High level (n=18)	Low level (n=17)	P value
Gender	Male	11	12	0.55
	Female	7	5	
Age	≦60 years old	8	9	0.62
	>60 years old	10	8	
Tumor location	colon	9	10	0.60
	rectum	9	7	
Tumor size	≦5 cm	10	8	0.62
	>5 cm	8	9	
TNM stage	I+II	13	5	0.01*
	III+IV	5	12	

*P < 0.05 was considered as significant. The cutoff value was the medium value of MIR31HG expression.

### Cell Culture

The CRC cell lines [RKO (Cat. No. CRL-2577), SW480 (Cat. No.CCL-228), SW620 (Cat. No. CCL-227), LoVo (Cat. No. CCL-229), and HCT116(Cat. No. CRL-2577)], and human umbilical vein endothelial cells (HUVECs, Cat.No. CRL-1730) used in this study were all bought from the GuangZhou Jennio Biotech Co., Ltd. (China). The cells are originated from American Type Culture Collection (ATCC, Rockville, MD, USA). Human colonic epithelial cells (HCoEpiC; Cat.No.2950) were purchased from Shanghai Zhongqiaoxinzhou Biotech (China). The above cells were cultured in the RPMI-1640 (Cat.No. C22400500BT, Thermo Fisher Scientific, Inc., USA), or DMEM medium (Cat.No.11330032, Thermo Fisher Scientific, Inc., USA) containing 10% fetal bovine serum (Cat.No. 10099-141, Gibco, Grand Island, New York, USA), 100 U/mL penicillin and 0.1 mg/mL streptomycin (Cat.No. ST488, Beyotime, Shanghai, China). The medium was maintained in an incubator with 5% CO_2_ and saturated humidity at 37°C. The trypsinization and sub-culture were performed with 0.25% trypsin when the cells had a 70%~80% fusion rate. The medium was altered every 2 days. RPMI-1640 medium (Cat.No. C22400500BT) and 0.25% trypsin (Cat.No. 25200072) were provided by Thermo Scientific Hyclone, Inc. (Utah, USA).

### Cell Transfection

When the cell fusion rate reached 70%~80%, CRC cells (RKO, SW480, SW620, LoVo and HCT116) and HUVECs were transfected with MIR31HG overexpression plasmids, small RNA (si)-MIR31HG, YY1 overexpression plasmids, miR-361-3p mimics and their negative controls (vector, si-NC, miR-NC), which were all provided by Guangzhou Ribo Biotechnology Co., Ltd (China). All transfections were performed using Lipofectamine^®^2000 (Cat.No. 11668030, Thermo Fisher Scientific, Inc., USA). The transfected cells were screened by quantitative real-time PCR (qRT-PCR) 48 hours after the transfection and then incubated at 37°C with 5% CO_2_ and saturated humidity until the experiment was finished.

### qRT-PCR

After cell transfection, the total RNA of CRC cells and HUVECs was isolated with the TRIzol reagent, and the RNA concentration was determined. Then, the RNA was reversely transcribed into cDNA following the instructions of PrimeScript™ II 1st Strand cDNA Synthesis Kit (Cat.No. 6210A-1, TaKaRa, Tokyo, Japan). qRT-PCR was performed using SYBR^®^ Premix Ex Taq™ II (Cat.No. RR820Q, TakaRa, Dalian, China). The reaction conditions include: pre-denaturation at 95°C for 30 s, denaturation at 95°C for 5 s, and annealing/extension at 60°C for 30 s. A total of 40 cycles were implemented. miR-361-3p was detected using All-inOne™ miRNA qPCR Detection kit (Cat.No. AOMD-Q050, GeneCopoeia, Guangzhou, China) and all procedures were performed according to the instructions of the manufacturers. β-actin served as the endogenous control of MIR31HG, YY1, STAT1, STAT3, ZEB1, NF-κB and SOX2, while U6 was for miR-361-3p. The relative expression of target genes was calculated by 2^-ΔΔCT^. Primer sequences of target genes were shown in the following table:

**Table:** 

Genes	Primer sequences (5’→3’)
MIR31HG	F: AATGACTGGTCTACGTGGGG
	R: GGGTGATTGAGGGCTCTACA
YY1	F: TTGCTCAGTCAACTAACCTGAAAT
	R: GAGGCA TATTTATTCCCAATCACAC
STAT1	F: GTATGCCATCCTCGAGAGCT
	R: TACCACTGAGACATCCTGCC
STAT3	F: AGAAGGAGGCGTCACTTTCA
	R: TTTCCGAATGCCTCCTCCTT
ZEB1	F: AGGCGAGAGTAGTGAGCAAG
	R: GTGTGTGTGTGTGTGTGTGT
NF-kB	F: ACACCGTGTAAACCAAAGCC
	R: CAGCCAGTGTTGTGATTGCT
SOX2	F: TGATGGAGACGGAGCTGAAG
	R: GCTTGCTGATCTCCGAGTTG
miR-361-3p	F: GCCGCTCCCCCAGGTGTGATT
	R: GTGCAGGGTCCGAGGT
β-actin	F: CTCCATCCTGGCCTCGCTGT
	R: GCTGTCACCTTCACCGTTCC
U6	F: CAACAGGCTCGTGAAAGACC
	R: GTTCGTCAACCTAGCGCAG

### 3-(4,5-Dimethylthiazol-2-yl)-2,5-Diphenyltetrazolium Bromide (MTT) Assay

CRC cells (RKO, SW480, SW620, LoVo and HCT116) and HUVECs in the logarithmic growth phase were seeded into 96-well plates after adjusting the cell concentration to 1×10^3^ cells/mL, and 4 replicates were set in each well. The stably transfected cells were taken, and the primary medium was discarded at the 24th, 48th, and 72th hour after cell transfection. Then, 20 μL MTT solution (5 mg/mL) (Cat.No. HY-15924, MedChemExpress, New Jersey, USA) was added to each well and further cultured for 4 hours. Next, the medium in the well was removed, and 150 μL dimethyl sulfoxide (Cat.No. D8370, Solarbio, Beijing, China) was added to each well and shaken on a shaker at low speed for 10 min to fully dissolve the crystals. A microplate reader was adopted to measure the absorbance at 450 nm of each well, and the average value of the four wells was statistically analyzed.

### The Colony Formation Experiment

Stably transfected CRC cells and HUVECs were inoculated into culture dishes at the same density (300 cells/dish) and incubated at 37°C with 5% CO_2_ and saturated humidity. After 2 weeks, they were dyed with crystal violet solution (0.1% crystal violet) and observed under a light microscope (DM1000; Leica Microsystems GmbH) for counting. A colony was defined if it contained more than 50 cells.

### Transwell Assay

Stably transfected CRC cells were taken, trypsinized with 0.25% trypsin) (Cat.No. HY-15924, MedChemExpress, New Jersey, USA) 24 hours after the transfection, and collected. Afterward, the cells were resuspended with serum-free RPMI1460 complete medium and adjusted to reach a cell density of 1×10^5^ cells/mL. The cells were inoculated in the upper chamber of a Transwell cell with an 8 μm pore size membrane, and the lower chamber was 500 μL RPMI-1460 complete medium containing 10% PBS. After culturing for 6 hours, the unmigrated cells on the membranes were wiped off, and the migrated cells were immobilized with 4% paraformaldehyde and stained with crystal violet. Five representative high-magnification fields on the membranes were randomly chosen to calculate transmembrane cells, and the mean value of the three repetitive wells was adopted to represent tumor cell invasion.

### Glucose and Lactic Acid Detection

Stably transfected CRC cells and HUVECs were taken. The fresh medium was adopted to replace the original one 24 hours after the transfection. After further culture for 24 hours, the medium was collected using the lactate assay kit (Cat.No. MAK064, Sigma-Aldrich, USA) and glucose assay kit (Cat.No. GAHK20, Sigma-Aldrich, USA) to measure lactic acid and glucose levels.

### Glycolysis Stress Test

CRC cells and HUVECs stably transfected for 48 hours were taken for detection. The commercial Seahorse XF Glycolysis Stress Test Kit (Cat.No.103020-100, Agilent Technologies, Suzhou, China) was employed to test glycolysis levels following the protocol in the manual. Key parameters of glycolysis were reflected by the extracellular acidification rate (ECAR) reported by this method.

### Tube Formation Assay

In order to evaluate the angiogenesis of HUVECs, a Matrigel’s tube formation assay was performed *in vitro*. Briefly, Matrigel (Cat. No. 356234, BD company) was coated on the 96-well plates (50 μl per well) for 12 hours on 4°C. HUVECs (transfected with MIR31HG overexpression plasmids or Vector) were digested, centrifugated and suspended in DMEM with 10% FBS. About 10000 cells suspended in 100 μl culture medium were cultured on the Matrigel-coated plates for 24 h. Tube formation was evaluated by light microscopy. ImageJ (version 1.52a) was used to quantify the number of capillary-like structures.

### Western Blot (WB)

Stably transfected CRC cells and HUVECs were used for protein analysis. Tumor tissues of nude mice were treated with a homogenizer and centrifuged. The primary cell culture medium and the supernatant of tumor tissues were discarded, and the total protein was isolated using the RIPA lysis buffer (Cat.No. P0013B, Beyotime, Shanghai, China). Then, 50 μg of total protein was added into 12% polyacrylamide gel, and electrophoresis was conducted at 100 V for 2 hours. The protein was then transferred to polyvinylidene fluoride (PVDF) membranes under a current of 300 mA. After being blocked with 5% skimmed milk powder at room temperature (RT) for 1 hour, the membranes were rinsed with TBST 3 times (10 min each). Then, the Anti-PKM2-antibody (1:1000, ab137852), Anti-GLUT1-antibody (1:100000, ab115730), Anti-HK2-antibody (1:1000, ab209847), Anti-CD31-antibody (1:1000, ab32457), Anti-VEGFA-antibody (1:1000, ab46154), Anti-YY1-antibody (1:2000, ab109237), Anti-HIF-1α-antibody (1:1000, ab179483), Anti-TIMP1-antibody (1:2000, ab211926), Anti-ANGPT1-antibody (1:1000, ab183701), and Anti-β-actin-antibody (1:1000, ab8226) were added and incubated overnight at 4°C. After being rewashed with TBST, the membranes were incubated with Goat Anti-Rabbit (1:2500, ab6721) at RT for 1 hour. Subsequently, the membranes were cleaned with TBST 3 times (10 min each). Finally, ECL development was carried out, and the images were saved, and the gray value of each protein was analyzed by Image J. The above antibodies were purchased from Abcam (MA, USA).

### The Xenograft Experiment in Nude Mice

Sixty nude mice (6 weeks-old, body weight: 20-22g) were selected as the experimental subjects to establish the xenograft model. All mice were housed and maintained under specific pathogen-free conditions free of water and food. CRC cells (SW480 and HCT116) stably transfected with MIR31HG overexpression plasmids and their negative controls were selected, and the blank control group was set. The cells were adjusted to reach a density of 2×10^8^ cells/mL when they were in the logarithmic growth phase. Afterward, 0.1 mL cell suspension was subcutaneously injected into the left forelimb armpit of each nude mouse. Within 4 weeks after the injection, the survival status and body weight of the mice, as well as the number of lung metastases, were monitored. Meanwhile, the weight, the longest diameter (a) and shortest diameter (b) of the tumor stripped from the newly dead mice were measured. Tumor volume (*V*)=0.5×a×b^2^. For evaluating the lung metastasis of CRC cells, CRC cells (SW480 and HCT116) stably transfected with MIR31HG overexpression plasmids and their negative controls were injected into the tail vein of the nude mice. Four weeks later, the mice were euthanized and their lungs were removed. The images and number of metastatic nodules in the lungs were captured and counted. Moreover, immunohistochemistry was conducted to detect VEGFA (1:200, ab52917) and KI67 (1:150, ab15580) in the formed tumor tissues according to previous study (24). The antibodies were all bought from Abcam company. The primary antibodies include All experiments were approved by the Animal Care and Use Committee of Fouth Affilated Hospital of Anhui Medical University and performed in accordance with institutional guidelines.

### Immunofluorescence Assay

CRC cells (SW480 and HCT116) and HUVECs stably transfected with MIR31HG overexpression plasmids were selected. The above cells were inoculated in 24-well plates at 5×10^6^ cells/well, immobilized with 4% paraformaldehyde for 15 min at RT, and washed with PBS 3 times (5 min each time). After membrane-breaking with 0.3% Triton-PBS for 5 min, the cells were rinsed with PBS 3 times (5 min/time). Then, they were added to 2% BSA-10% goat serum-PBS and blocked at 37°C for 45 min. Next, 1% BSA-PBS was added to dilute the primary anti-YY1-Antibody (1:2000, ab109237), which was refrigerated overnight at 4°C. The next day, the cells were washed with PBS for 5 min (3 times). The secondary Goat Anti-Rabbit antibody (1:2500, ab6721) was diluted with 1% BSA-PBS and incubated at RT for 45 min with the cells, which were washed with PBS for 5 min (3 times). Subsequently, the DAPI (Cat.No. C1002, Beyotime Biotechnology Co., Ltd, Shanghai, China) was diluted with PBS (at a ratio of 1:4) to stain the cells for 5 min, and the cells were washed with PBS 3 times (5 min/time). The images were observed and collected under a fluorescence microscope (BX53, Olympus, Japan).

### The Dual-Luciferase Reporter Assay

According to the prediction results on lncBase v.2 (http://carolina.imis.athena-innovation.gr/diana_tools/web/index.php?r=lncbasev2%2Findex) and Targetscan (http://www.targetscan.org/vert_72/), the luciferase reporter vectors used in this study (MIR31HG -WT, MIR31HG -MT, YY1-WT, and YY1-MT) were all constructed by Promega Corporation (Madison, WI, USA). MIR31HG -MT was mutant at Chromosome 9 21455665-21455689 and YY1-MT was mutant at 4772-4778 of the YY1 mRNA UTR. SW480 cells were seeded into 48-well plates at 4.5×10^4^ cells/well. The Lipofectamine^®^ 2000 Transfection Reagent (Cat.No. 11668030, Thermo Fisher Scientific, Inc., USA) was adopted to co-transfect the above-mentioned luciferase reporter vectors with miR-361-3p mimics or miR-NC into SW480 cells when the cell fusion rate reached 70%-80%. The luciferase activity was determined on the Dual-Luciferase^®^ Reporter Assay System (Cat.No. E1910, Promega Corporation, Madison, WI, USA) 48 hours after the transfection, following the manufacturer’s instructions.

### RNA Immunoprecipitation (RIP)

SW480 cells were transfected with miR-NC or miR-361-3p mimics, respectively. Forty-eight hours after the transfection, RIP detection was performed on stably transfected SW480 cells using the Magna-RIPTM RNA binding protein immunoprecipitation kit (Cat.No. 17-701, Millipore, Bedford, MA, USA). Then the cells were incubated with the anti-Ago2 antibody or negative control IgG (Thermo Fisher Technology, Shanghai, China). Finally, the relative expression of MIR31HG, miR-361-3p, and YY1 was compared by qRT-PCR.

### Statistical Analysis

Data from this study were processed with GraphPad Prism 8 (GraphPad Software, USA) and expressed as mean ± standard deviation (x ± s). Kaplan-Meier curve was drawn to clarify the association between MIR31HG expression and the patients’ survival time. The correlation between MIR31HG and miR-361-3p was studied by Pearson correlation analysis. The independent sample *t* test was utilized for data comparison between the two groups, and one-way analysis of variance was used for multivariate comparison. *P*<0.05 indicated statistical significance.

## Results

### Expression Characteristics of MIR31HG in CRC Tissues

**Figure 1:**
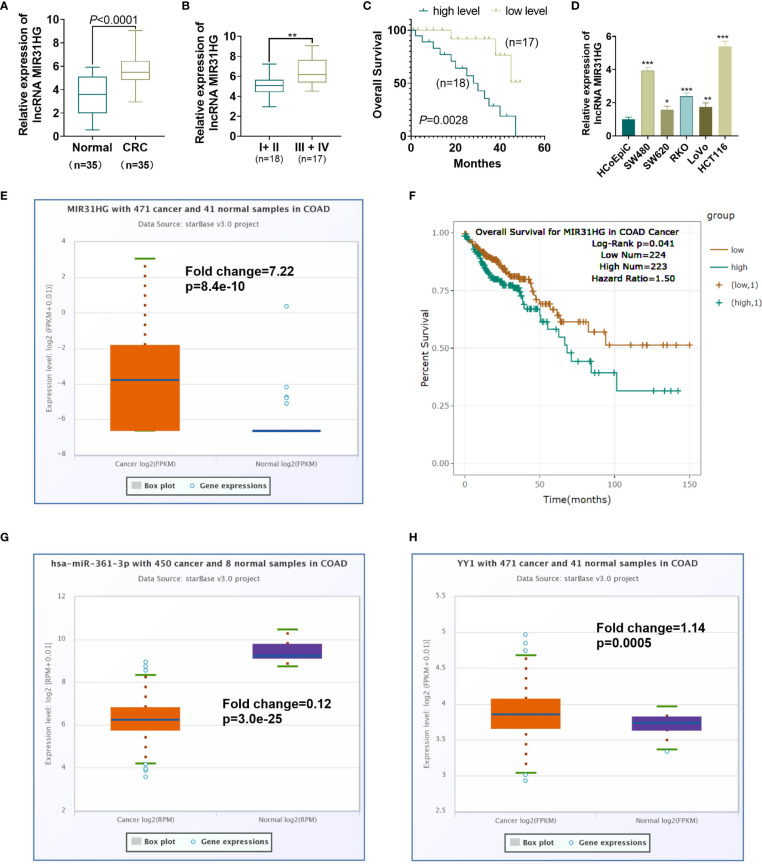
Expression characteristics of MIR31HG in CRC. **(A)** qRT-PCR was conducted to test the MIR31HG expression in 35 CRC tissues and 20 normal tissues. ***P < 0.001(*vs*. normal group). **(B)** The level of MIR31HG in two groups of CRC tissues (stage I-II *vs*.stage III-IV) was analyzed. **(C)** K-M plotter was used for analyzing the association between MIR31HG level with the overall survival of CRC patients. The survival curve of the 35 CRC patients was shown. *P*=0.0448 (*vs*. low MIR31HG level group). **(D)** The MIR31HG profile in CRC cells (Caco-2, RKO, SW480, SW620, LoVo and HCT116) and Human colonic epithelial cells (HCoEpiC). *P < 0.05, **P < 0.01, ***P < 0 .001 (*vs*. HCoEpiC group). (n=3). **(E)** MIR31HG level in CRC tissues (data of cancers were downloaded from TCGA project *via* Genomic Data Commons) was analyzed *via* ENCORI (The Encyclopedia of RNA Interactomes, http://starbase.sysu.edu.cn/index.php). **(F)** That the higher level of MIR31HG was associated with poorer survival of CRC patients analyzed by ENCORI database. G-H. miR-361-3p **(G)** and YY1 **(H)** levels in CRC tissues were analyzed through ENCORI (The Encyclopedia of RNA Interactomes, http://starbase.sysu.edu.cn/index.php).

We implemented qRT-PCR to examine the MIR31HG expression in tissues and cells. The results showed that the MIR31HG expression in CRC tissues was significantly higher than that in normal colorectal tissues (*P*<0.0001, **Figure 1A**). The MIR31HG level in different stages of CRC was analyzed. The result indicated that MIR31HG had higher level in stage III + IV CRC tissues compared with that in the stage I + II CRC tissues (*P*<0.001, **Figure 1B**). A further 50-month survival analysis of 35 CRC patients demonstrated that high levels of MIR31HG significantly shortened survival (*P*=0.0448, **Figure 1C**). The cellular experiment results manifested that the MIR31HG profile in CRC cells (Caco-2, RKO, SW480, SW620, LoVo and HCT116) was significantly higher than that in Human colonic epithelial cells (HCoEpiC) (*P*<0.05, **Figure 1D**). Moreover, we analyzed MIR31HG level in Colon Adenocarcinoma (COAD) tissues (data of cancers were downloaded from TCGA project *via* Genomic Data Commons) *via* ENCORI (The Encyclopedia of RNA Interactomes, http://starbase.sysu.edu.cn/index.php). It was found that MIR31HG has a fold change of 7.22 compared with that in the normal tissues (p=8.4e-10, **Figure 1E**). Higher level of MIR31HG was associated with poorer survival of CRC patients (p=0.041, **Figure 1F**). Moreover, the ENCORI database showed that miR-361-3p was significantly downregulated in COAD (Fold change=0.12, p=3.0e-25), while YY1 was significantly upregulated in COAD (Fold change=1.14, p=0.0005, **Figures 1G, H**). Herein, MIR31HG might be an oncogene in CRC.

### Overexpressing MIR31HG Facilitated the Proliferation and Glycolysis of CRC Cells and Angiogenesis of Endothelial Cells

**Figure 2:**
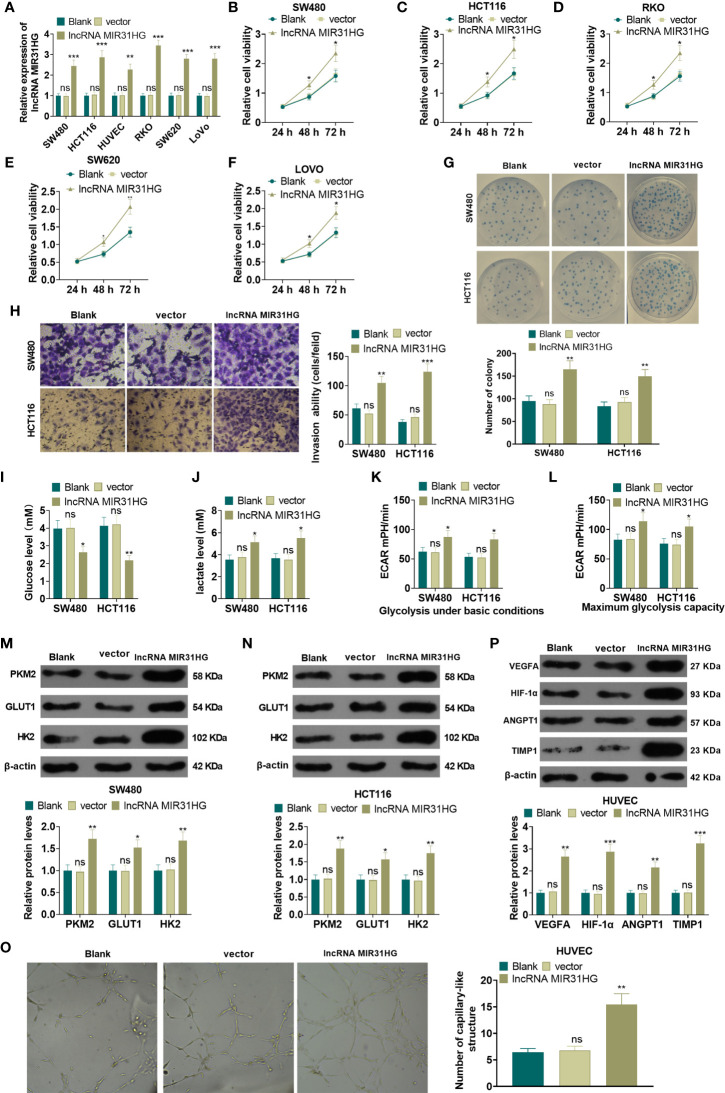
Overexpressing MIR31HG facilitated CRC cell proliferation and glycolysis and endothelial cell angiogenesis. The MIR31HG overexpression model was constructed in CRC cell lines (Caco-2, RKO, SW480, SW620, LoVo and HCT116) and HUVEC cell lines. **(A)** The transfection validity was examined by qRT-PCR. **(B–F)** MTT assay was used to detect the proliferation of Caco-2, RKO, SW480, SW620, LoVo and HCT116 cells. **(G)** CRC cell proliferation was tested by colony formation assay. **(H)** Transwell assay were employed to evaluate CRC cell invasion. **(I, J)** The glucose and lactic acid production of CRC cells were measured using a glucose detection kit and lactic acid detection kit. **(K, L)** Glycolytic stress test was utilized to monitor the glycolytic level of CRC cells. **(M, N)** The expression of glycolysis-related proteins (PKM2, GLUT1, and HK2) in CRC cells was compared by WB. **(O)** Tube formation assay was used for evaluating the angiogenesis of HUVECs *in vitro*. The number of capillary-like structures was counted. **(P)** Western blot was used for detecting the expression of angiogenesis markers (including VEGFA, ANGPT1, HIF-1α and TIMP1) in HUVECs with MIR31HG overexpression. NS p > 0.05 (*vs*. Blank group), *P < 0.05, **P < 0.01, ***P < 0.01 (*vs*. vector group). (n=3).

The MIR31HG overexpression model was constructed in CRC cell lines (RKO, SW480, SW620, LoVo and HCT116) and HUVEC cell lines. qRT-PCR was conducted to test the transfection validity. The results illustrated that the expression of MIR31HG in RKO, SW480, SW620, LoVo, HCT116, and HUVECs was significantly higher than that of the vector group after transfection of MIR31HG overexpression plasmids (*P*<0.05, **Figure 2A**). MTT assay was used for evaluating cell proliferation. As shown in **Figures 2B–F**, the CRC cells (RKO, SW480, SW620, LoVo, HCT116) with overexpressed MIR31HG had enhanced proliferation compared with the Vector group. Colony formation results confirmed that overexpressing MIR31HG heightened SW480 and HCT116 cell proliferation (*P*<0.05, **Figure 2G**). Additionally, the Transwell assay testified that overexpressing MIR31HG significantly enhanced SW480 and HCT116 cell invasion (*P*<0.05, **Figure 2H**). The glucose detection kit and lactate detection kit showed that overexpressing MIR31HG significantly reduced the glucose level and promoted the production of lactate in SW480 and HCT116 cells (*P*<0.05, **Figures 2I, J**). The glycolytic stress test illustrated that overexpressing MIR31HG increased basal and maximum glycolytic levels of SW480 and HCT116 cells (*P*<0.05, **Figures 2K, L**). Besides, the expression of glycolysis-related proteins in SW480 and HCT116 cells was monitored by WB. Moreover, the gray intensity analysis of the protein map showed that the protein expressions of PKM2, GLUT1 and HK2 were increased after overexpressing MIR31HG (*P*<0.05, **Figures 2M, N**). We performed tube formation assay to detect the angiogenic ability of HUVECs. It was found that HUVECs with upregulated MIR31HG grew more capillary-like structures (*vs*.Vector group, **Figure 2O**). Furthermore, high levels of MIR31HG significantly facilitated the expression of angiogenesis markers (including VEGFA, ANGPT1, HIF1A and TIMP1) in HUVECs (*vs*. the vector group) (*P*<0.05, **Figure 2P**). These findings suggested that overexpressing MIR31HG strengthened the proliferation and glycolysis of CRC cells and the angiogenesis of HUVECs.

### Knocking Down MIR31HG Repressed CRC Cell Proliferation and Glycolysis and Endothelial Cell Angiogenesis

**Figure 3:**
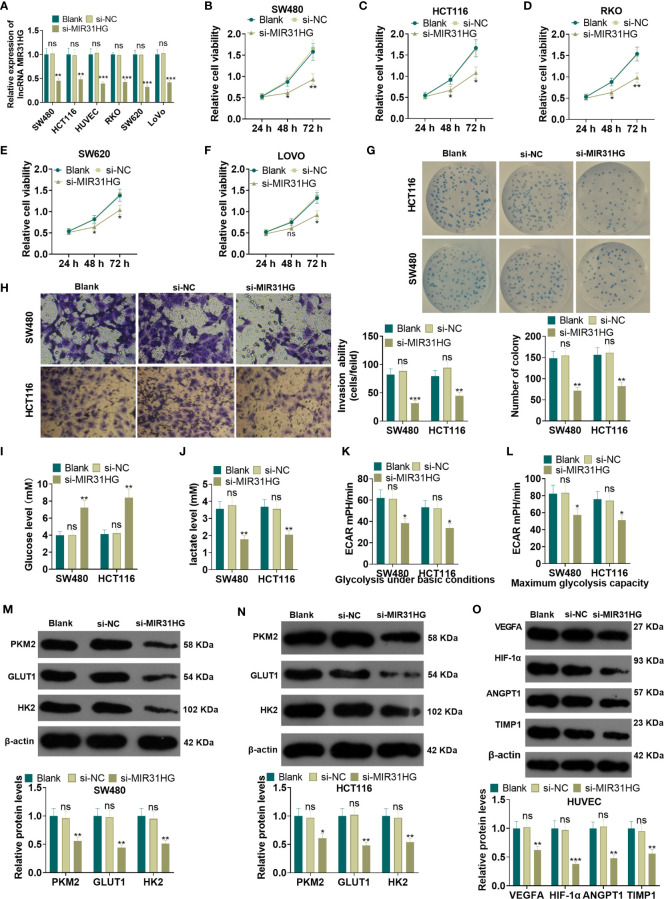
Knocking down MIR31HG abated the proliferation and glycolysis of CRC cells and angiogenesis of endothelial cells. The MIR31HG knockdown model was constructed in CRC cell lines (Caco-2, RKO, SW480, SW620, LoVo and HCT116) and HUVEC cell lines. **(A)** The transfection validity was examined by qRT-PCR. **(B–F)** MTT assay was used to detect the proliferation of Caco-2, RKO, SW480, SW620, LoVo and HCT116 cells. **(G)** CRC cell proliferation wastested by colony formation assay. **(H)**. Transwell assay were employed to evaluate CRC cell invasion. **(I, J)** The glucose and lactic acid production of CRC cells were measured using a glucose detection kit and lactic acid detection kit. **(K, L)** Glycolytic stress test was utilized to monitor the glycolytic level of CRC cells. **(M, N)** The expression of glycolysis-related proteins (PKM2, GLUT1, and HK2) in CRC cells was compared by WB. **(O)** Western blot was used for detecting the expression of angiogenesis markers (including VEGFA, ANGPT1, HIF-1α and TIMP1) in HUVECs with MIR31HG overexpression. NS p > 0.05 (*vs*. Blank group), *P < 0.05, **P < 0.01, ***P < 0.01 (*vs*. si-NC group). (n=3).

The MIR31HG knockdown model was constructed in CRC cell lines (RKO, SW480, SW620, LoVo, HCT116) and HUVEC cell lines. qRT-PCR demonstrated that the MIR31HG profile in RKO, SW480, SW620, LoVo, HCT116, and HUVECs was significantly declined after the transfection of si-MIR31HG (*P*<0.05, **Figure 3A**). Additionally, the MTT assay, and colony formation experiment were performed to detect cell proliferation. It was found that the proliferation of RKO, SW480, SW620, LoVo, HCT116 cells was significantly repressed after knocking down MIR31HG (*P*<0.05, **Figures 3B–G**). Besides, the Transwell assay illustrated that SW480 and HCT116 cell invasive ability was dampened after knocking down MIR31HG (*P*<0.05, **Figure 3H**). Moreover, glucose and lactic acid tests showed that the glucose level in SW480 and HCT116 cells was increased and the lactic acid level was decreased after MIR31HG knockdown (*P*<0.05, **Figures 3I, J**). Meanwhile, the glycolytic stress test confirmed that knocking down MIR31HG hampered basal and maximum glycolytic levels of SW480 and HCT116 cells (*vs*. the si-NC group) (*P*<0.05, **Figures 3K, L**). WB results testified that PKM2, GLUT1 and HK2 expressions were impeded in SW480 and HCT116 cells after knocking down MIR31HG (*P*<0.05, **Figures 3M, N**). Furthermore, knockdown of MIR31HG significantly repressed the angiogenesis markers (including VEGFA, ANGPT1, HIF1A and TIMP1) of HUVECs (*vs*. the si-NC group) (*P*<0.05, **Figure 3O**). In conclusion, knocking down MIR31HG hampered SW480 and HCT116 cell proliferation and glycolysis and HUVEC angiogenesis.

### MIR31HG Overexpression Promoted CRC Cell Growth and Lung Metastasis *In Vivo*

**Figure 4:**
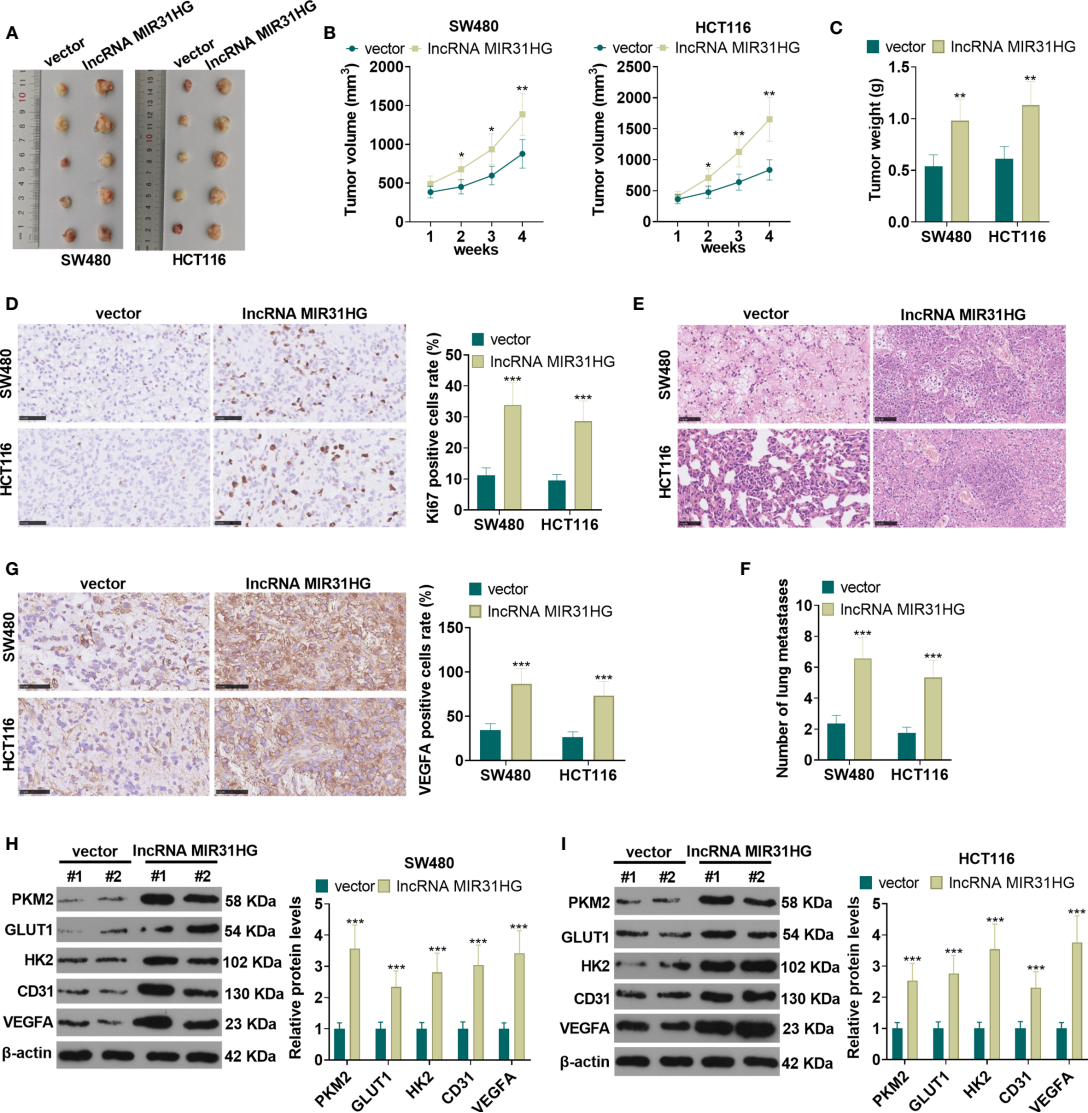
The role of MIR31HG in controlling CRC growth and metastasis *in vivo*. The suspension of SW480 and HCT116 cells overexpressing MIR31HG was used to construct the xenograft model *in vivo*. **(A)** The nude mice were sacrificed at the 4^th^ week after tumor cell injection, and the tumor images were shown. **(B, C)** Tumor volume and weight was counted. **(D)** IHC was used for detecting KI67 in the tumor tissues. The rate of KI67-positive cell was counted. **(E, F)** Lung metastasis assay was used for evaluating the metastasis of CRC cells with forced MIR31HG upregulation. The number lung metastase was counted. **(G)** IHC was used for detecting VEGFA in the tumor tissues. **(H, I)** WB was used to detect the expression of PKM2, GLUT1 and HK2 as well as CD31 and VEGFA in the tumor tissues. ns P > 0.05 (*vs* Blank group). *P < 0.05, **P < 0.01, ***P < 0.001 (*vs*.vector group). (n=5).

The suspension of SW480 and HCT116 cells with overexpressed MIR31HG was collected to build the xenograft model *in vivo*. The exfoliated tumor tissue in nude mice was shown in **Figure 4**. Tumor volume and weight analysis manifested that overexpressing MIR31HG significantly heightened the growth of tumor volume and weight (*vs*. the vector group) (*P*<0.05, **Figures 4B, C**). Additionally, overexpressing MIR31HG significantly increased the KI67-positive cell rate, indicating that MIR31HG promotes tumor cell proliferation (*P*<0.05, **Figure 4D**). Moreover, the number of lung metastases increased with MIR31HG overexpression (*P*<0.05, **Figures 4E, F**). IHC data showed that compared with the vector group, the tumors in MIR31HG group had enhanced VEGFA expression (**Figure 4G**). WB results revealed that overexpressing MIR31HG significantly promoted the expression of glycolysis-related proteins (PKM2, GLUT1, and HK2) and angiogenesis-related proteins (CD31 and VEGFA) (*P*<0.05, **Figures 4H, I**). These results confirmed that MIR31HG overexpression enhanced tumor growth, glycolysis and angiogenesis in SW480 and HCT116 cells.

### MIR31HG Up-Regulated YY1, Which Promoted MIR31HG Expression

**Figure 5:**
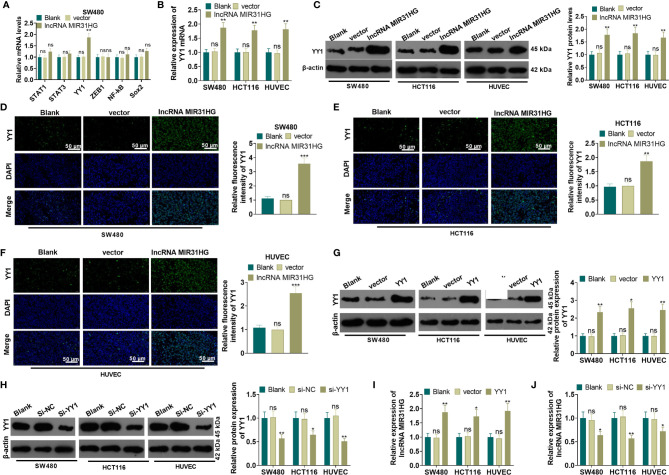
MIR31HG up-regulated YY1 and was promoted by YY1 feedback. The MIR31HG overexpression model was constructed in CRC cell lines (SW480 and HCT116) and HUVEC cell lines. **(A)** qRT-PCR was performed to detect the transcription factors (including STAT1, STAT3, YY1, ZEB1, NF-κB, Sox2) in SW480 cells. B-F. The YY1 expression was tested by qRT-PCR **(B)**, WB **(C)** and immunofluorescence assay **(D–F)**, respectively. **(G, H)**, YY1 overexpression and knockdown models were constructed in CRC cell lines and HUVEC cell lines, respectively. The expression of YY1 was detected by WB. **(I, J)**. qRT-PCR was used for detecting MIR31HG in these cells. ns *P*>0.05 (*vs*. Blank group). *P < 0.05, **P < 0.01, ***P < 0.001 (*vs*.vector/Si-NC group). (n=3).

The MIR31HG overexpression model was constructed in CRC cell lines (SW480 and HCT116) and HUVEC cell lines. We performed qRT-PCR to detect the mRNA level of five transcription factors (including STAT1, STAT3, YY1, ZEB1 and NF-κB) in SW480 cells. Compared with the vector group, overexpressing MIR31HG significantly promoted the expression of YY1 (*P*<0.05, **Figure 5A**). The YY1 mRNA profile was tested by qRT-PCR, WB, and immunofluorescence assay, respectively. As a result, the mRNA level, the protein expression, and the proportion of YY1-positive cell number were significantly elevated after overexpressing MIR31HG (*vs*. the vector group) (*P*<0.05, **Figures 5B–F**). Then, the overexpression and knockdown models of YY1 were constructed in CRC cell lines and HUVEC cell lines, respectively, and the transfection validity was tested by qRT-PCR and western blot. It turned out that the YY1 expression was significantly elevated (*vs*. the vector group, **Figure 5G**). Besides, compared with the si-NC group, the YY1 profile was significantly decreased (*P*<0.05, **Figure 5H**). Moreover, qRT-PCR results manifested that overexpressing YY1 facilitated the MIR31HG expression in CRC cells and HUVECs (*P*<0.05, **Figure 5I**), while knockdown of YY1 significantly reduced the expression of MIR31HG (*P*<0.05, **Figure 5J**). The above findings illustrated that MIR31HG up-regulated YY1 and was promoted by YY1 feedback.

### Overexpressing YY1 Reversed the Anti-Tumor Effects Mediated by MIR31HG Knockdown

**Figure 6:**
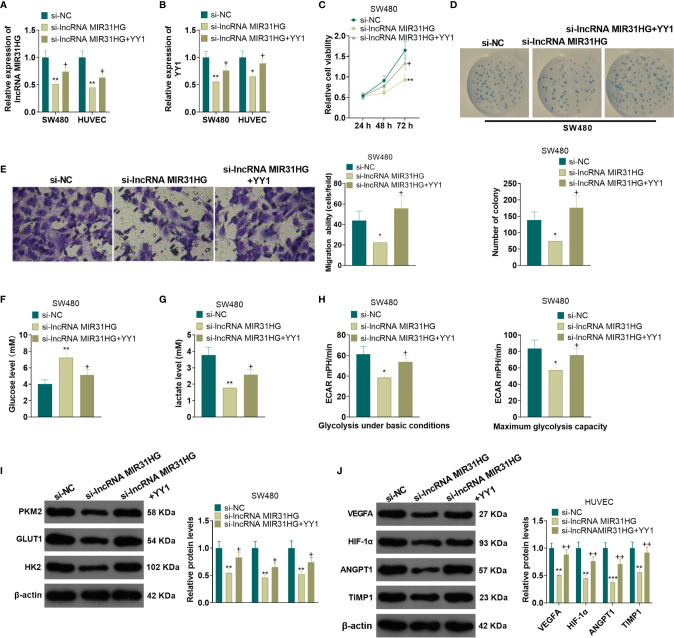
Overexpressing YY1 reversed the anti-tumor effect mediated by MIR31HG knockdown. The YY1 overexpression model was established in SW480 and HUVECs on the basis of MIR31HG knockdown. **(A, B)**. Expression of MIR31HG and YY1 was determined by qRT-PCR. **(C, D)** CRC cell proliferation was tested by MTT and colony formation assay. **(E)** Transwell assay were employed to evaluate CRC cell invasion. **(F, G)** The glucose and lactic acid production of CRC cells were measured using a glucose detection kit and lactic acid detection kit. **(H)** Glycolytic stress test was utilized to monitor the glycolytic level of CRC cells. **(I)** The expression of glycolysis-related proteins (PKM2, GLUT1, and HK2) in CRC cells was compared by WB. **(J)** Western blot was used for detecting the expression of angiogenesis markers (including VEGFA, ANGPT1, HIF-1α and TIMP1) in HUVECs with MIR31HG overexpression. *P < 0.05, **P < 0.01, ***P < 0.001. (*vs*. Si-NC group). +*P* < 0.05, ++*P* < 0.01 (*vs*. Si- MIR31HG group). (n=3).

A YYI overexpression model was constructed on the basis of MIR31HG knockdown. qRT-PCR results manifested that, compared with the si-MIR31HG group, the MIR31HG and YY1 expression was elevated in SW480 and HUVECs after the transfection of YY1 overexpression plasmids (*P*<0.05, **Figures 6A, B**). SW480 cell proliferation was tested by MTT and colony formation assay. It was found that compared with the si-MIR31HG group, SW480 cell proliferation was significantly heightened after overexpressing YY1 (*P*<0.05, **Figures 6C, D**). The Transwell assay illustrated that YY1 overexpression on the basis of MIR31HG knockdown significantly increased SW480 cell invasion (*P*<0.05, **Figure 6E**). The glucose and lactic acid tests showed that the glucose level significantly decreased, while the lactic acid level increased after overexpressing YY1 in SW480 cells (*P*<0.05, **Figures 6F, G**). The glycolysis stress test showed that the overexpression of YY1 up-regulated the basal and maximum glycolytic levels of SW480 cells (*vs*. the si-MIR31HG group) (*P*<0.05, **Figure 6H**). The expression of glycolysis-related proteins was compared by WB. As a result, PKM2, GLUT1 and HK2 expression was significantly heightened in SW480 cells after YY1 overexpression on the basis of MIR31HG knockdown (*P*<0.05, **Figure 6I**). Besides, compared with the si-MIR31HG group, overexpressing YY1 significantly increased angiogenesis markers (including VEGFA, ANGPT1, HIF1A and TIMP1) of HUVECs (*P*<0.05, **Figure 6J**). The above experimental results verified that overexpressing YY1 reversed the anti-tumor effect mediated by MIR31HG knockdown.

### MiR-361-3p Targeted MIR31HG and YY1

**Figure 7:**
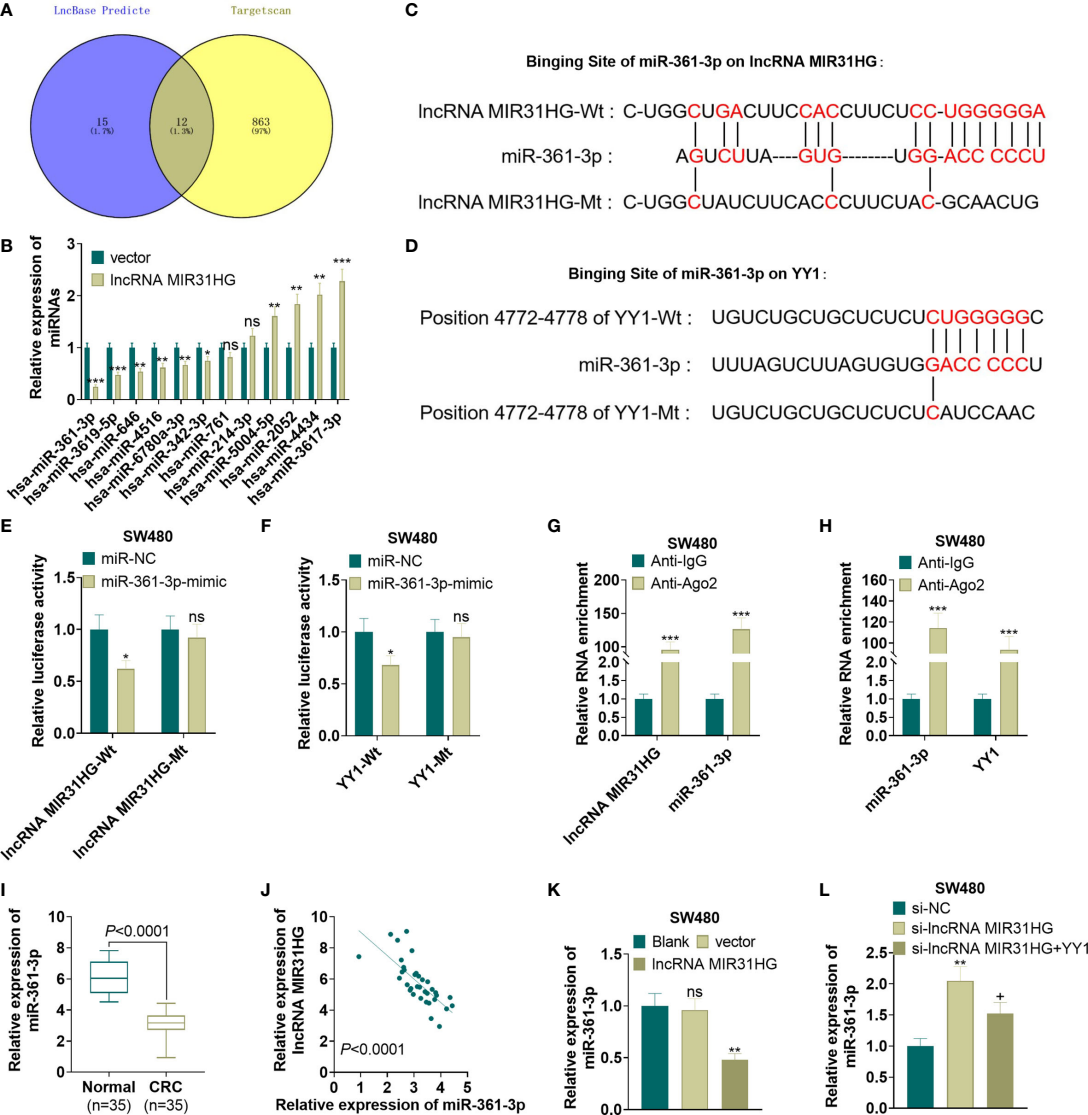
MiR-361-3p targeted MIR31HG and YY1. **(A)** The miRNA targets of MIR31HG and YY1 were predicted *via* lncBase v.2 (http://carolina.imis.athena-innovation.gr/diana_tools/web/index.php?r=lncbasev2%2Findex) and Targetscan (http://www.targetscan.org/vert_72/), respectively. 12 miRNAs were potential targets of MIR31HG and YY1 *via* Venn’s diagram analysis. **(B)** qRT-PCR was used to detect miR-361-3p in SW480 cells transfected with MIR31HG overexpression plasmids. **(C, D)**. The base binding sequences of miR-361-3p, MIR31HG and YY1 were shown. **(E-H)**. The binding relationship between miR-361-3p, MIR31HG and YY1 was examined by the dual-luciferase reporter assay and RIP assay. ns*P* > 0.05, *P < 0.05, ***P < 0.001 (*vs*. the miR-NC/Anti-IgG group). **(I)** Expression of miR-361-3p in CRC tissues and normal tissues was measured by qRT-PCR. ***P < 0.001(*vs*. normal group). **(J)** Correlation analysis of miR-361-3p and MIR31HG in CRC tissues. **(K)** The miR-361-3p expression in SW480 cells after overexpressing MIR31HG was examined by qRT-PCR. ns *P >* 0.05 (*vs*.Blank group). **P < 0.01 (*vs*. the vector group). **(L)**. qRT-PCR detected the miR-361-3p profile after knockdown of MIR31HG and overexpression of YY1. **P < 0.01 (*vs*. Si-NC group). +*P* < 0.05(*vs*. Si- MIR31HG group). (n=3).

For exploring the mechanism between MIR31HG and YY1, we predicted the miRNA targets of MIR31HG and YY1 *via* lncBase v.2 (http://carolina.imis.athena-innovation.gr/diana_tools/web/index.php?r=lncbasev2%2Findex) and Targetscan (http://www.targetscan.org/vert_72/), respectively. We found that 12 miRNAs were potential targets of MIR31HG and YY1 (**Figure 7A**). Next, we detected those 12 miRNAs in SW480 cells transfected with MIR31HG overexpression plasmids. Interestingly, the data showed that miR-361-3p was most significantly inhibited by MIR31HG (p<0.001, **Figure 7B**). The binding sites between miR-361-3p and MIR31HG, YY1 and miR-361-3p were shown in **Figures 7C, D**. Next, the binding relationships between the three were further tested by the dual-luciferase reporter assay and RIP experiment. The results showed that miR-361-3p overexpression in SW480 cells decreased the luciferase activities of cells transfected with MIR31HG -WT and YY1-WT, but had no significant effect on cells transfected with MIR31HG-MT and YY1-MT (*P*>0.05, **Figures 7E, F**). Additionally, the amount of miR-361-3p, MIR31HG and YY1 enriched by anti-AGO2 were significantly higher than those in the anti-IgG group (*P*<0.05, **Figures 7G, H**). What’s more, qRT-PCR testified that the miR-361-3p expression in CRC tissues was lower than that in normal tissues (*P*<0.0001, **Figure 7I**). Pearson analysis manifested that the expression of miR-361-3p and MIR31HG in CRC tissues was negatively correlated (*R* ^2^ = 0.4445, *P*<0.0001, **Figure 7J**). As shown in **Figure 7K**, overexpressing MIR31HG obviously impeded the miR-361-3p profile in SW480 cells (*vs*. the vector group) (*P*<0.05). Meanwhile, knocking down MIR31HG significantly elevated the miR-361-3p expression in SW480 cells compared with the si-NC group (**Figure 7L**, *P*<0.05). The above results indicated that miR-361-3p targeted MIR31HG and YY1.

### Overexpressing miR-361-3p Dampened the Proliferation and Glycolysis of CRC Cells and Angiogenesis of Endothelial Cells

**Figure 8:**
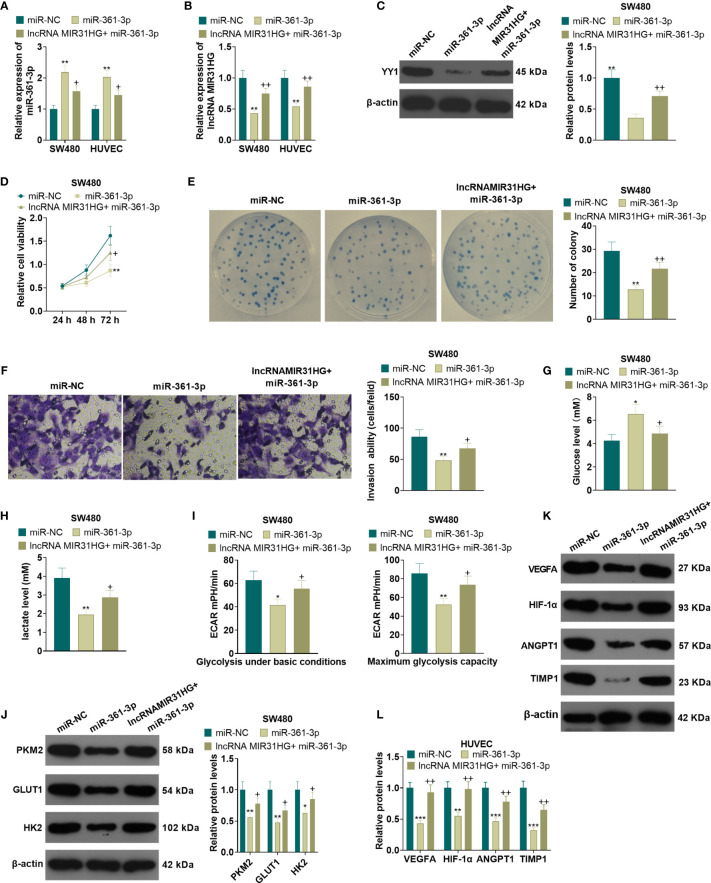
Overexpressing miR-361-3p hampered CRC cell proliferation and glycolysis and endothelial cell angiogenesis. After the transfection of miR-361-3p mimics, MIR31HG overexpression plasmids were transfected into SW480 and HUVECs. **(A, B)**. The expression of miR-361-3p and MIR31HG was determined by qRT-PCR. **(C)** WB was conducted to verify the YY1 expression. **(D, E)** CRC cell proliferation was tested by MTT and colony formation assay. **(F)** Transwell assay were employed to evaluate CRC cell invasion. **(G, H)**. The glucose and lactic acid production of CRC cells were measured using a glucose detection kit and lactic acid detection kit. **(I)** Glycolytic stress test was utilized to monitor the glycolytic level of CRC cells. **(J)** The expression of glycolysis-related proteins (PKM2, GLUT1, and HK2) in CRC cells was compared by WB. **(K, L)**. Western blot was used for detecting the expression of angiogenesis markers (including VEGFA, ANGPT1, HIF-1α and TIMP1) in HUVECs with miR-361-3p or MIR31HG overexpression. *P < 0.05, **P < 0.01, ***P < 0.001. (*vs*.miR-NC group). +*P* < 0.05, ++*P*<0.01 (*vs*. miR-361-3p group). (n=3). ***P <0.001.

**Figure 9:**
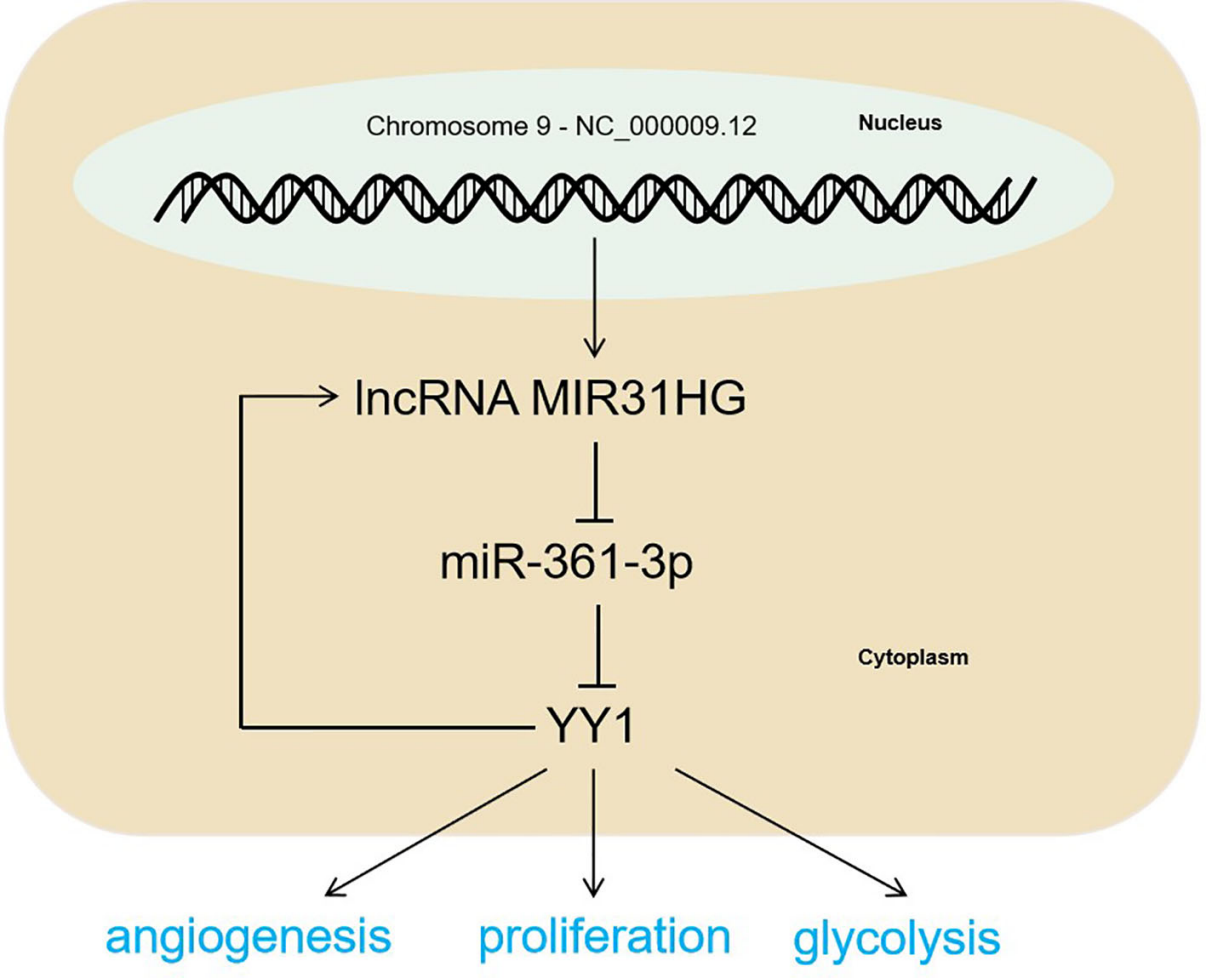
The mechanism diagram. The MIR31HG-miR-361-3p-YY1 positive feedback loop accelerated CRC progression by promoting the proliferation and glycolysis of CRC cells and the angiogenesis of HUVECs.

After the miR-361-3p mimics transfection, SW480 and HUVECs were transfected with MIR31HG overexpression plasmids. qRT-PCR manifested that the miR-361-3p expression was elevated and the MIR31HG profile was decreased in SW480 and HUVECs after the transfection of miR-361-3p mimics. Additionally, compared with the miR-361-3p group, the miR-361-3p+ MIR31HG group had reduced miR-361-3p levels and elevated MIR31HG expression (*P*<0.05, **Figures 8A, B**). The YY1 expression was examined by WB. As a result, overexpressing miR-361-3p significantly impeded the YY1 expression, and MIR31HG overexpression on this basis significantly facilitated the YY1 expression (*P*<0.05, **Figure 8C**). MTT and colony formation experiments illustrated that SW480 cell proliferation was impeded after overexpressing miR-361-3p. In contrast, compared with the miR-361-3p group, overexpressing MIR31HG significantly enhanced cell proliferation (*P*<0.05, **Figures 8D, E**). The Transwell assay manifested that overexpressing miR-361-3p impeded the invasion of SW480 cells, while overexpressing MIR31HG partially reversed this effect (*P*<0.05, **Figure 8F**). Glucose and lactic acid were detected in the supernatant of SW480 cells, and the results illustrated that miR-361-3p overexpression significantly elevated the glucose level and repressed the production of lactic acid. However, overexpression of MIR31HG reversed the effect of miR-361-3p (*P*<0.05, **Figures 8G, H**). Further glycolytic stress test manifested that miR-361-3p overexpression significantly repressed basal and maximum glycolytic levels of SW480 cells, and this effect was weakened by MIR31HG overexpression (*P*<0.05, **Figure 8I**). WB demonstrated that overexpressing miR-361-3p declined the levels of PKM2, GLUT1 and HK2 in SW480 cells, while overexpressing MIR31HG on the basis of miR-361-3p overexpression up-regulated these proteins (*P*<0.05, **Figure 8J**). The WB confirmed that miR-361-3p overexpression dampened the angiogenesis markers (including VEGFA, ANGPT1, HIF1A and TIMP1) of HUVECs. On the contrary, overexpressing MIR31HG promoted HUVEC angiogenesis (*vs*. the miR-361-3p group) (*P*<0.05, **Figures 8K, L**). Thus, we confirmed that MIR31HG reversed miR-361-3p mediated anti-tumor effects *via* sponging miR36-3p (**Figure 9**).

## Discussion

CRC is a common malignant tumor in the gastrointestinal tract and a threat to human health (25). This study probes the molecular mechanisms of CRC and provides a reference and basis for its clinical treatment. Our study found that MIR31HG was overexpressed in CRC tissues and cells. Both cellular and *in vivo* experiments confirmed that overexpressing MIR31HG accelerated CRC progression. Further mechanism studies illustrated that MIR31HG targeted and reversely regulated the miR-361-3p expression. In contrast, miR-361-3p targeted and hampered the expression of YY1, and YY1 had a positive promoting effect on MIR31HG expression.

It is well known that lncRNAs exert an extensive and critical role in modulating cell growth, development and metabolism, especially in tumor development (26–28). As a vital member of lncRNA, MIR31HG has been verified as an oncogene in multiple tumors. For example, in non-small cell lung cancer (NSCLC), MIR31HG functions as an independent predictor of poor overall survival (OS) of NSCLC patients, and also promotes tumor progression (29). In the study conducted by Yajing Li, it was found that MIR31HG had upregulated level in i cervical carcinoma tissues. The functional assay showed that knockdown of MIR31HG inhibits cervical carcinoma cell growth and invasiveness *via* inhibiting miR-361-3p as an endogenous ‘sponge’. miR-361-3p targets epithelial membrane protein 1 (EMP1) (30). This is an interesting study revealing that the MIR31HG-miR-361-3p-EMP1 axis plays a vital role in cervical carcinoma progression, and it also indicates a novel axis of MIR31HG-miR-361-3p in tumor development. Interestingly, another study found that MIR31HG is a recurrence-associated six-lncRNA, and it was significantly enriched in proliferation and angiogenesis, cell death, as well as critical cancer pathways in colon cancer (31). This study suggests that MIR31HG might play a vital in CRC progression. Our study confirmed that MIR31HG has up-regulated level in CRC tissues and cells. Forced upregulation of MIR31HG aggravated the proliferation, invasion and metastasis of CRC cells, which further confirms its oncogenic role in CRC.

Studies have stated that changes in metabolites are associated with prognosis, survival and recurrence of CRC (32). Glycolysis is a common energy metabolic pathway in living cells (33). On the other hand, ATP produced by increased glycolysis is the main source of energy supply for cancer cells. Changes in energy metabolism are the biochemical fingerprints of cancer cells and one of the markers of cancer (34). In addition, glycolysis-related proteins (PKM2, GLUT1, and HK2) have been used as important reference factors for detecting the glycolysis level (35–38). Moreover, the expression of glycolysis-related proteins is positively correlated with the level of glycolysis. Therefore, we adopted the glucose, lactic acid and glycolysis levels was the evaluation indicators of CRC. Besides, angiogenesis involving tumors is conducive to tumor cell proliferation and distant metastasis (39). We employed HUVECs as experimental subjects to conduct the tube formation experiment to verify the regulation of MIR31HG or miR-361-3p on angiogenesis. Our results illustrated that overexpressing MIR31HG elevated the glycolysis of CRC cells (SW480 and HCT116) and the angiogenesis of HUVECs, whereas overexpressing miR-361-3p had the opposite effect. The finding convinces us that MIR31HG expedites CRC progression by modulating glycolysis and angiogenesis *via* targeting miR-361-3p.

Multiple transcription factors (TFs) are activated during tumorigenesis and tumor progression. The TFs, such as STAT1, STAT3, ZEB1, NF-κB, have been found to mediate CRC progression *via* modulating the expression of lncRNAs (40–43). YY1 is a multifunctional protein that can activate or inhibit gene expression according to changes in conditions (44). Moreover, several studies have shown that YY1 is overexpressed in various cancers, and high levels of YY1 are associated with malignant phenotypes of several cancers, including CRC (45, 46). What’s more, YY1 has a role in regulating non-coding RNA, thus getting involved in CRC progression. For example, a recent study showed that YY1 heightens Ovarian Cancer Cell cell proliferation *via* miR-526b-3p/E2F1 (47). Besides, YY1-induced lncRNA DDX11-AS1 accelerates CRC progression by targeting the miR-873/CLDN7 axis. These studies suggest that YY1 acts as an oncogene in CRC (48). In another study, Ihira K et al. performed profound researches and suggested that EZH2 and YY1 were upstream mediators of miR-361 in endometrial cancer, which functions as a tumor suppressor microRNA *via* controlling cell proliferation and invasion (49). Here, we found that overexpression of YY1 reversed the anti-tumor effect mediated by MIR31HG knockdown and that YY1 positively promoted MIR31HG expression. Further mechanism studies illustrated that miR-361-3p targeted and negatively regulated YY1. Therefore, those results suggested that MIR31HG-miR-361-3p-YY1 forms a positive feedback loop in CRC.

Overall, this study revealed that MIR31HG heightened the proliferation and glycolysis of CRC cells as well as the angiogenesis of HUVECs. The mechanism study showed that this effect was exerted through the positive feedback loop of MIR31HG-miR-361-3p-YY1 axis, which provides the research direction and theoretical basis for target therapy of CRC.

## Data Availability Statement

The original contributions presented in the study are included in the article/**Supplementary Material**. Further inquiries can be directed to the corresponding author.

## Ethics Statement

The studies involving human participants were reviewed and approved by The Medical Ethics Committee of The Fourth Affiliated Hospital of Anhui Medical University. The patients/participants provided their written informed consent to participate in this study. The animal study was reviewed and approved by The Medical Ethics Committee of The Fourth Affiliated Hospital of Anhui Medical University.

## Author Contributions

Conceived and designed the experiments: DL. Performed the experiments: TG. Statistical analysis: DL, SP, MW, and YL. Wrote the paper: TG. All authors contributed to the article and approved the submitted version.

## Funding

This work was supported by the basic and clinical cooperative research and promotion program of Anhui Medical University (grant number: 2020xkjT045), National and provincial key specialty construction plan (grant number: Z155080000004).

## Conflict of Interest

The authors declare that the research was conducted in the absence of any commercial or financial relationships that could be construed as a potential conflict of interest.

## Publisher’s Note

All claims expressed in this article are solely those of the authors and do not necessarily represent those of their affiliated organizations, or those of the publisher, the editors and the reviewers. Any product that may be evaluated in this article, or claim that may be made by its manufacturer, is not guaranteed or endorsed by the publisher.
