# Supplementary figures and images for: A Positive Feedback Loop of lncRNA MIR31HG-miR-361-3p -YY1 Accelerates Colorectal Cancer Progression Through Modulating Proliferation, Angiogenesis, and Glycolysis
Source: Front Oncol. 2021 Aug 17;11:684984. doi: 10.3389/fonc.2021.684984 (PMC8416113; doi:10.3389/fonc.2021.684984)

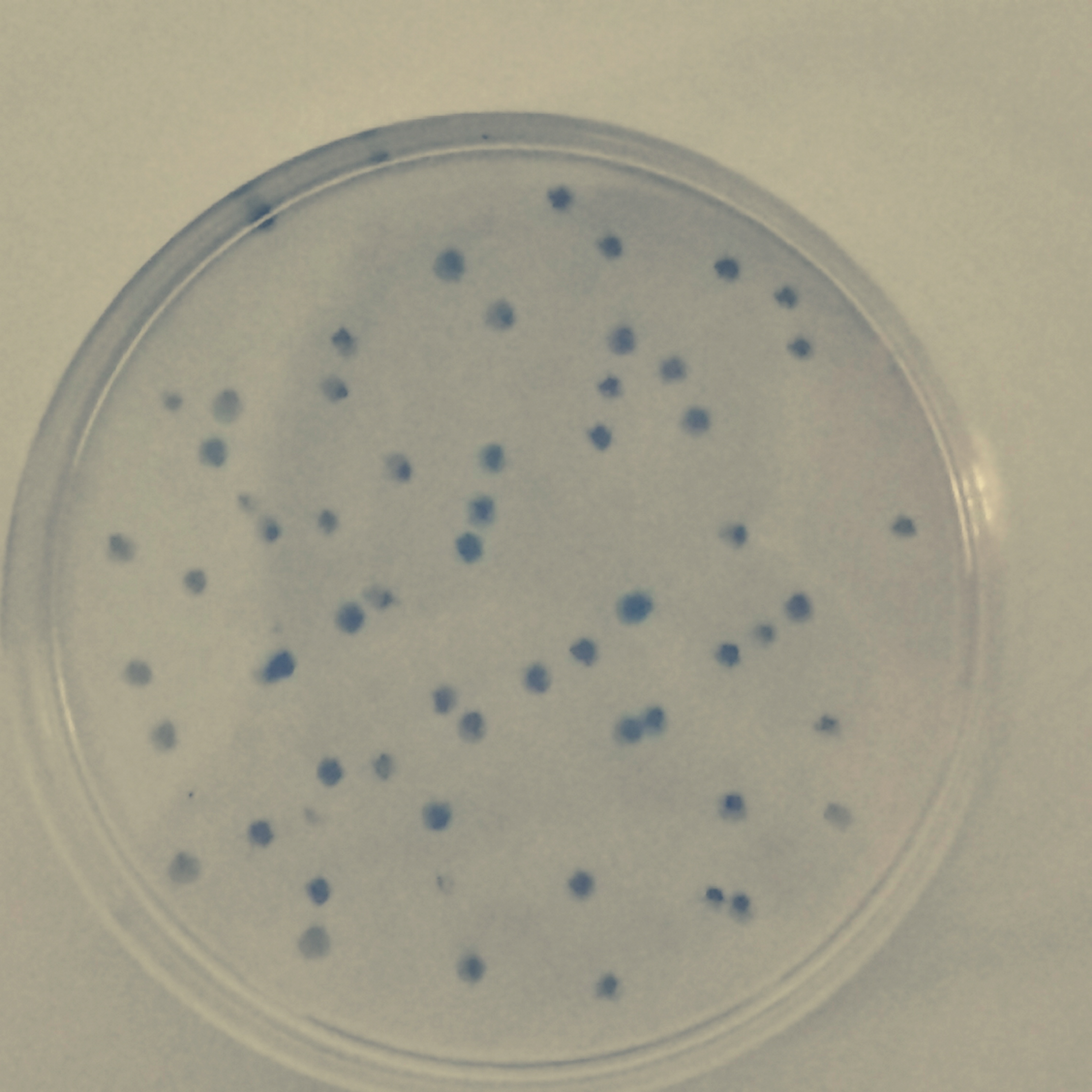

Supplement: Supplementary file 1 [file DataSheet_1.zip › fig2/HCT116-colony-1.jpg]

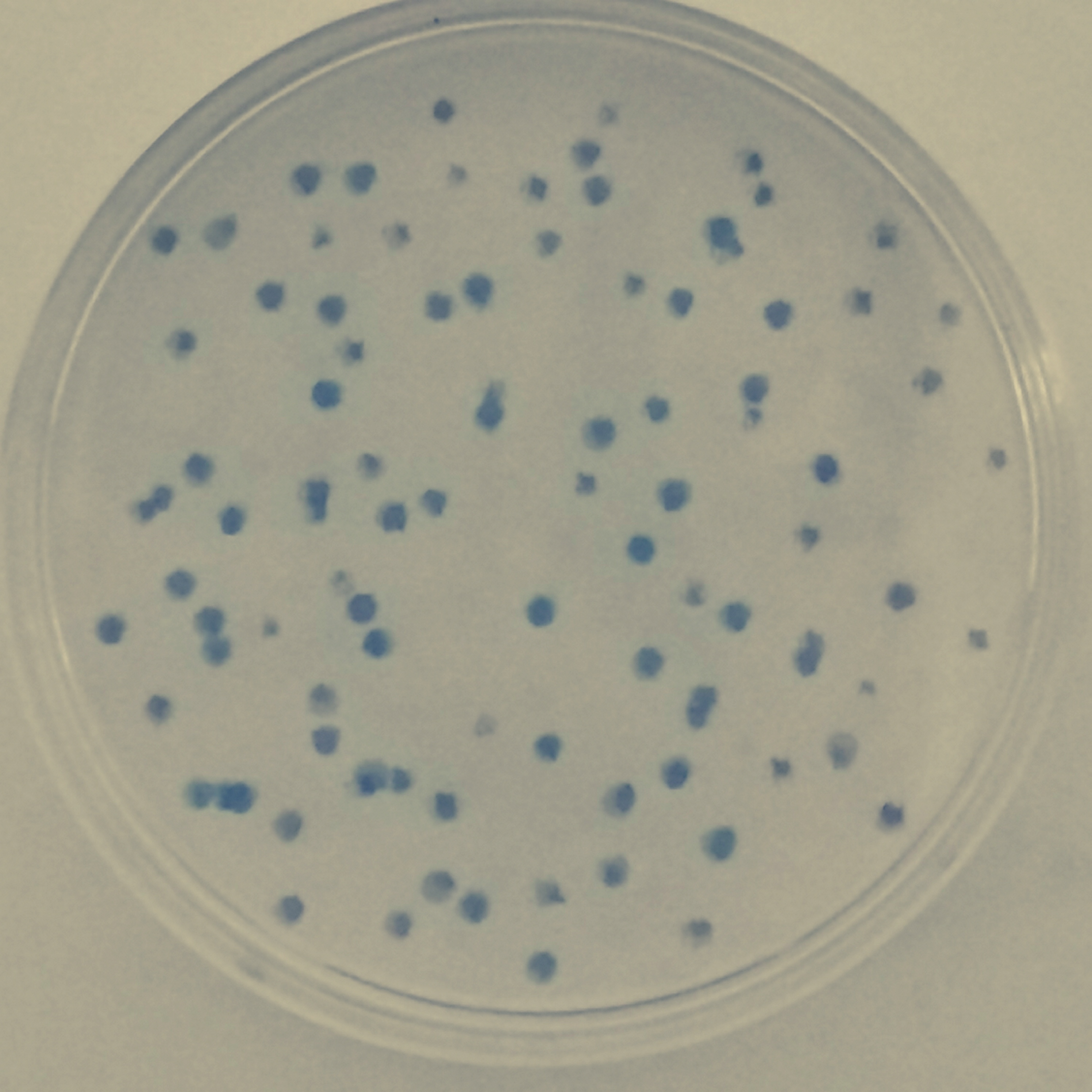

Supplement: Supplementary file 1 [file DataSheet_1.zip › fig2/HCT116-colony-2.jpg]

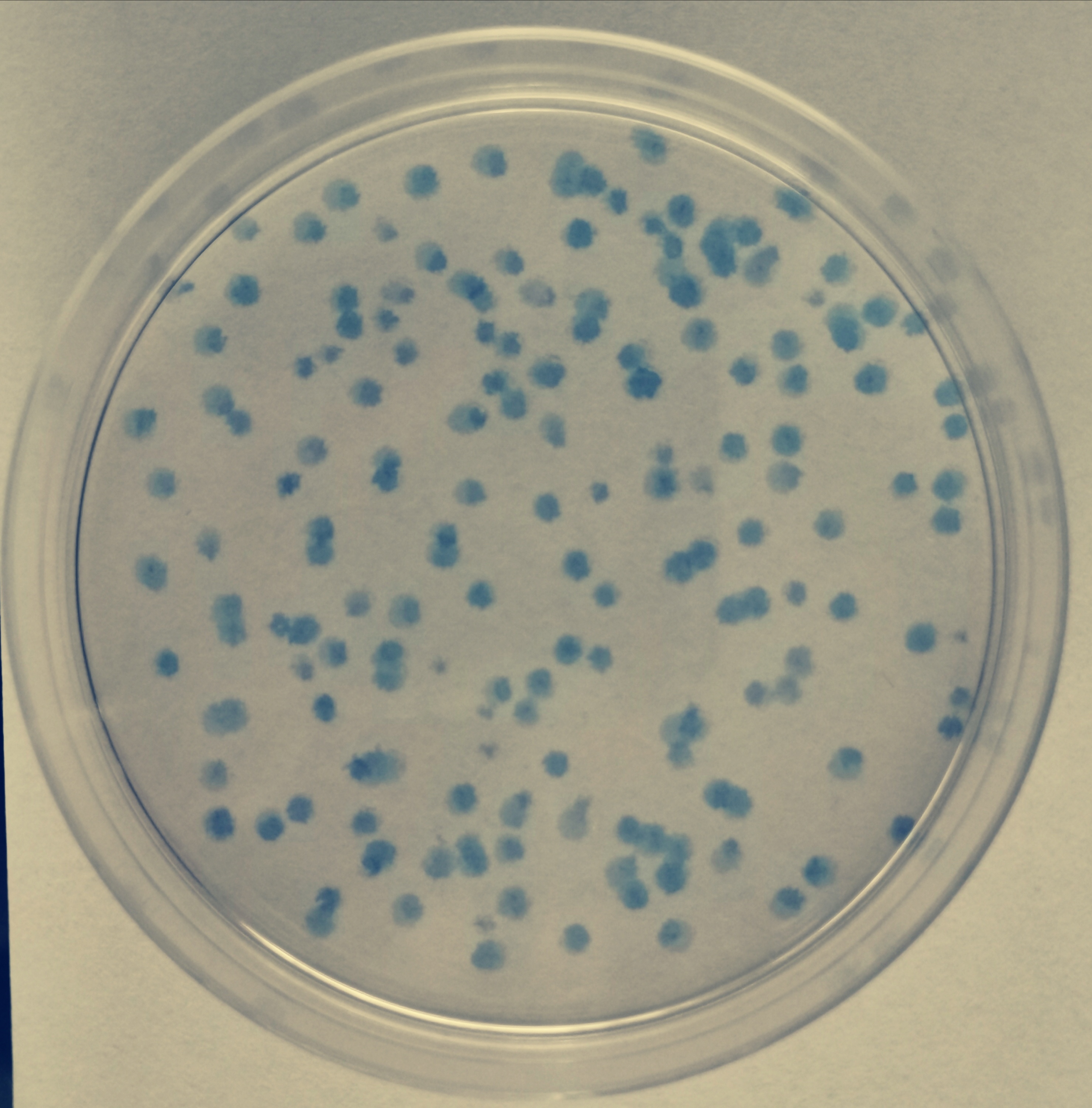

Supplement: Supplementary file 1 [file DataSheet_1.zip › fig2/HCT116-colony-3.jpg]

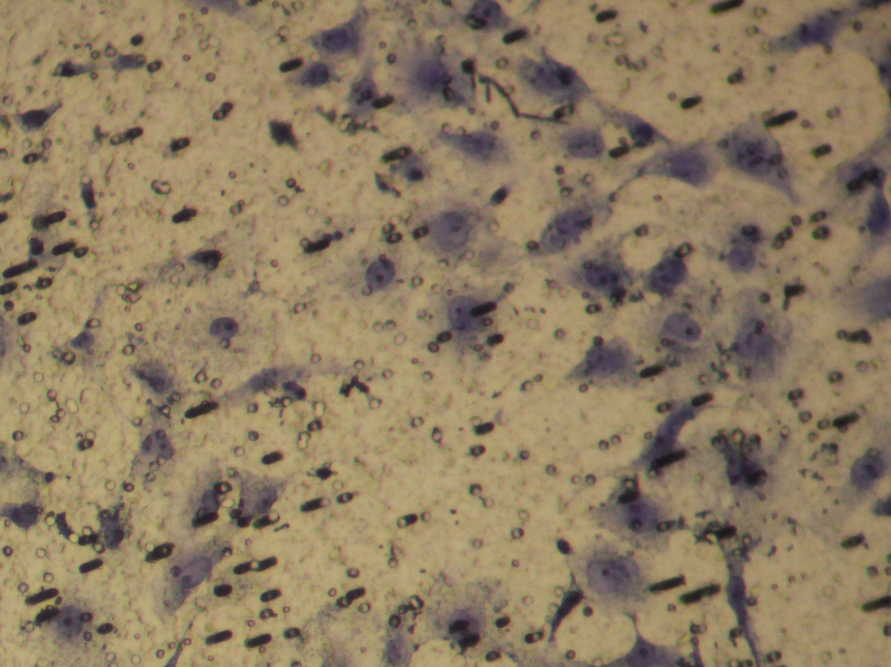

Supplement: Supplementary file 1 [file DataSheet_1.zip › fig2/HCT116-invasion-1.png]

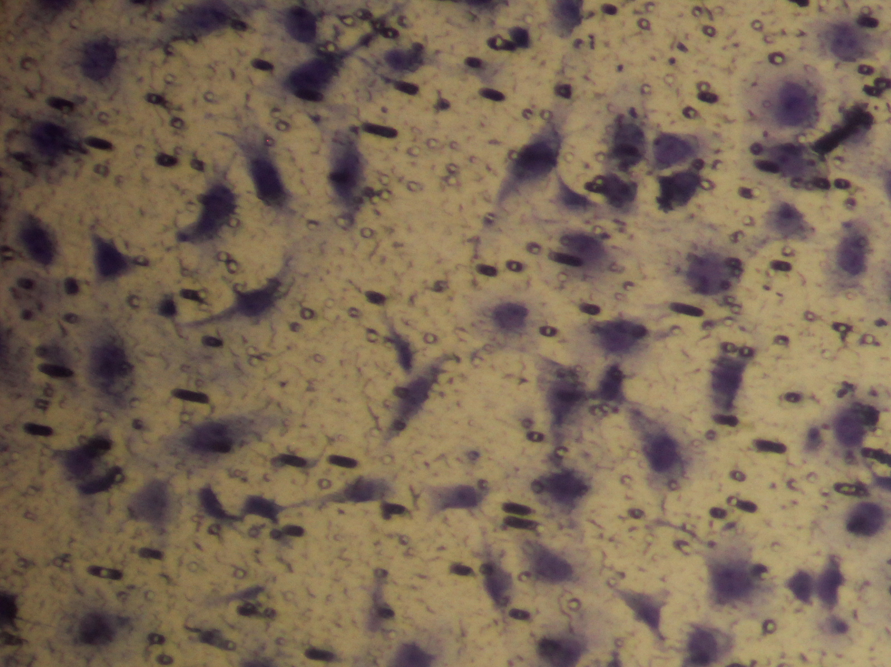

Supplement: Supplementary file 1 [file DataSheet_1.zip › fig2/HCT116-invasion-2.png]

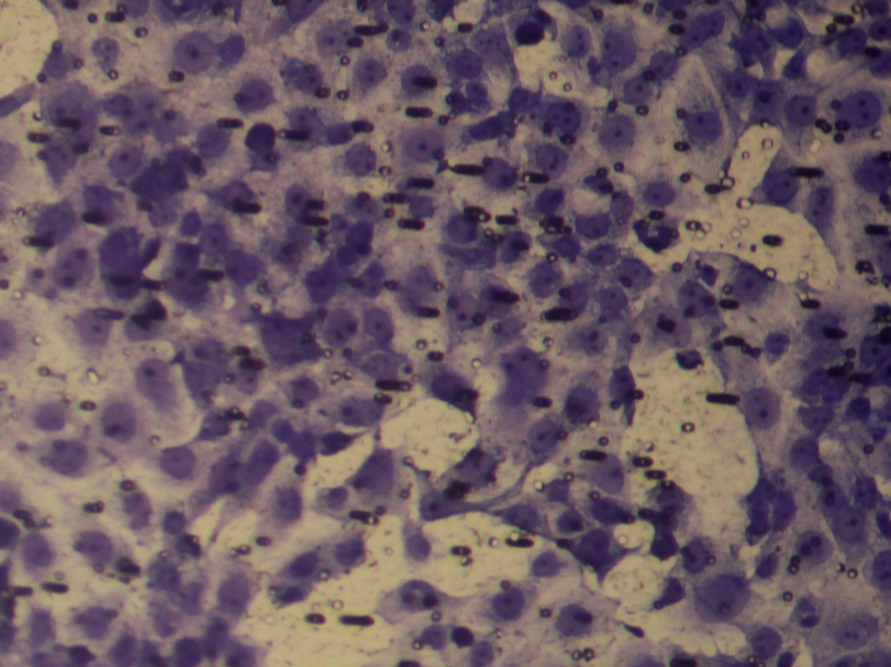

Supplement: Supplementary file 1 [file DataSheet_1.zip › fig2/HCT116-invasion-3.png]

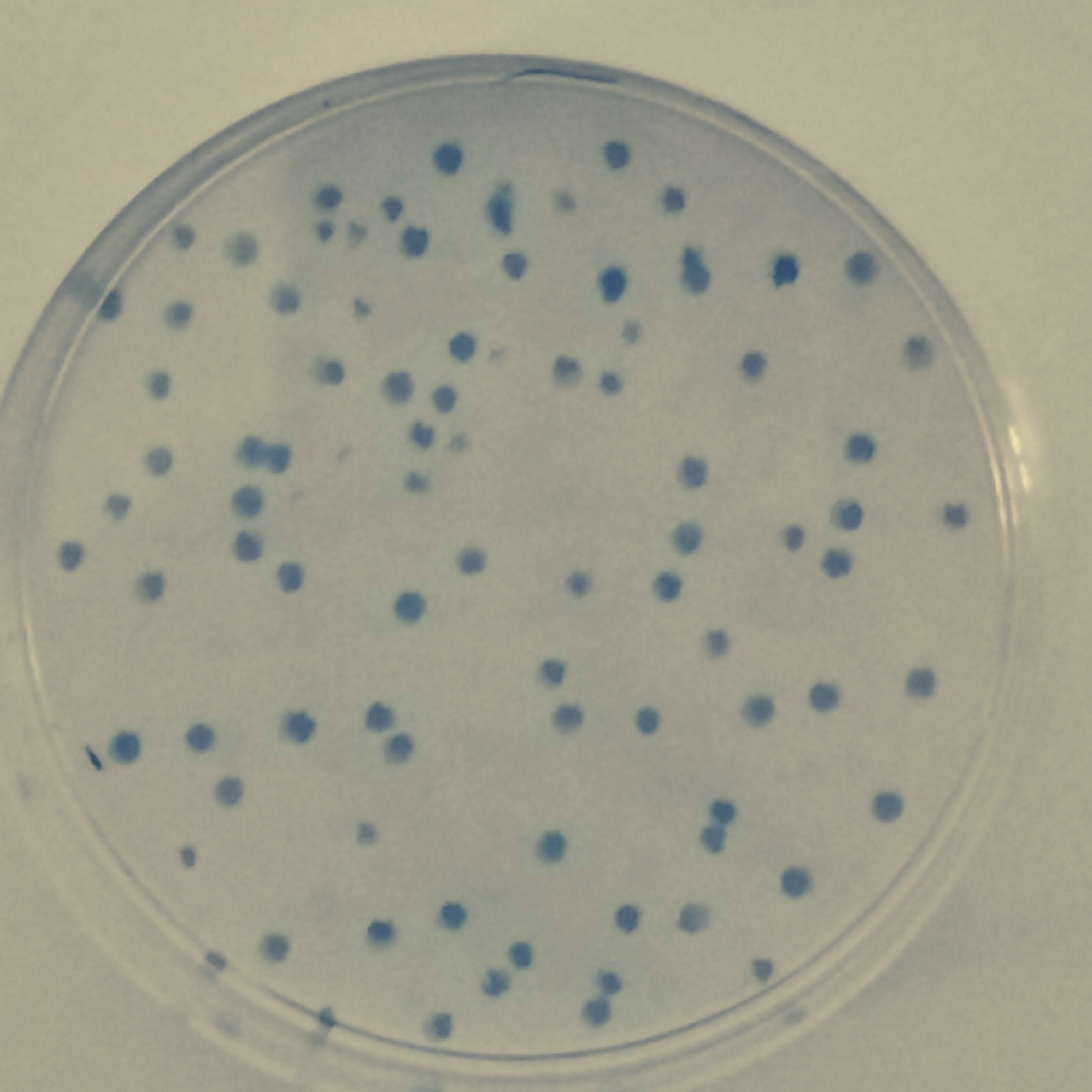

Supplement: Supplementary file 1 [file DataSheet_1.zip › fig2/sw480-colony-1.jpg]

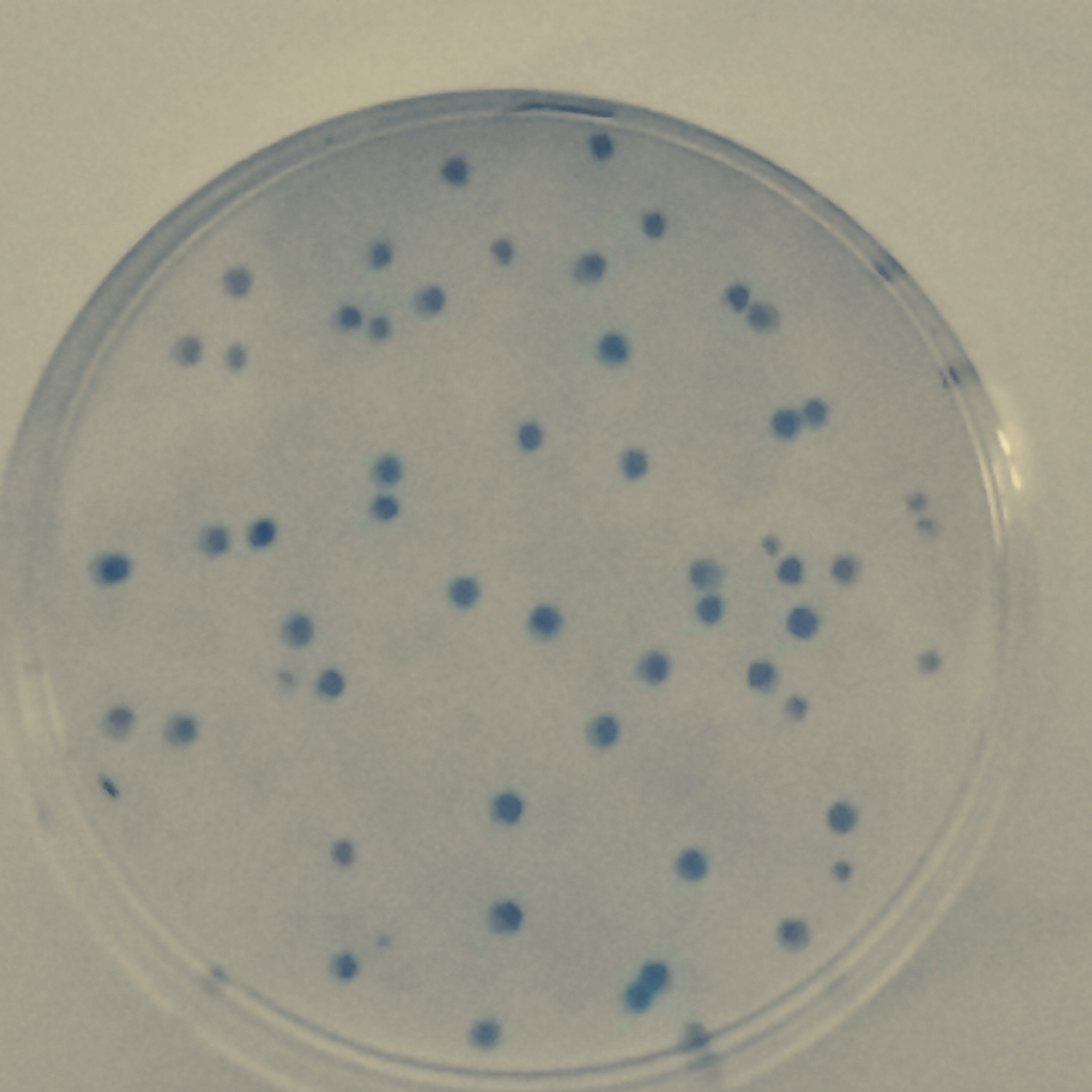

Supplement: Supplementary file 1 [file DataSheet_1.zip › fig2/sw480-colony-2.jpg]

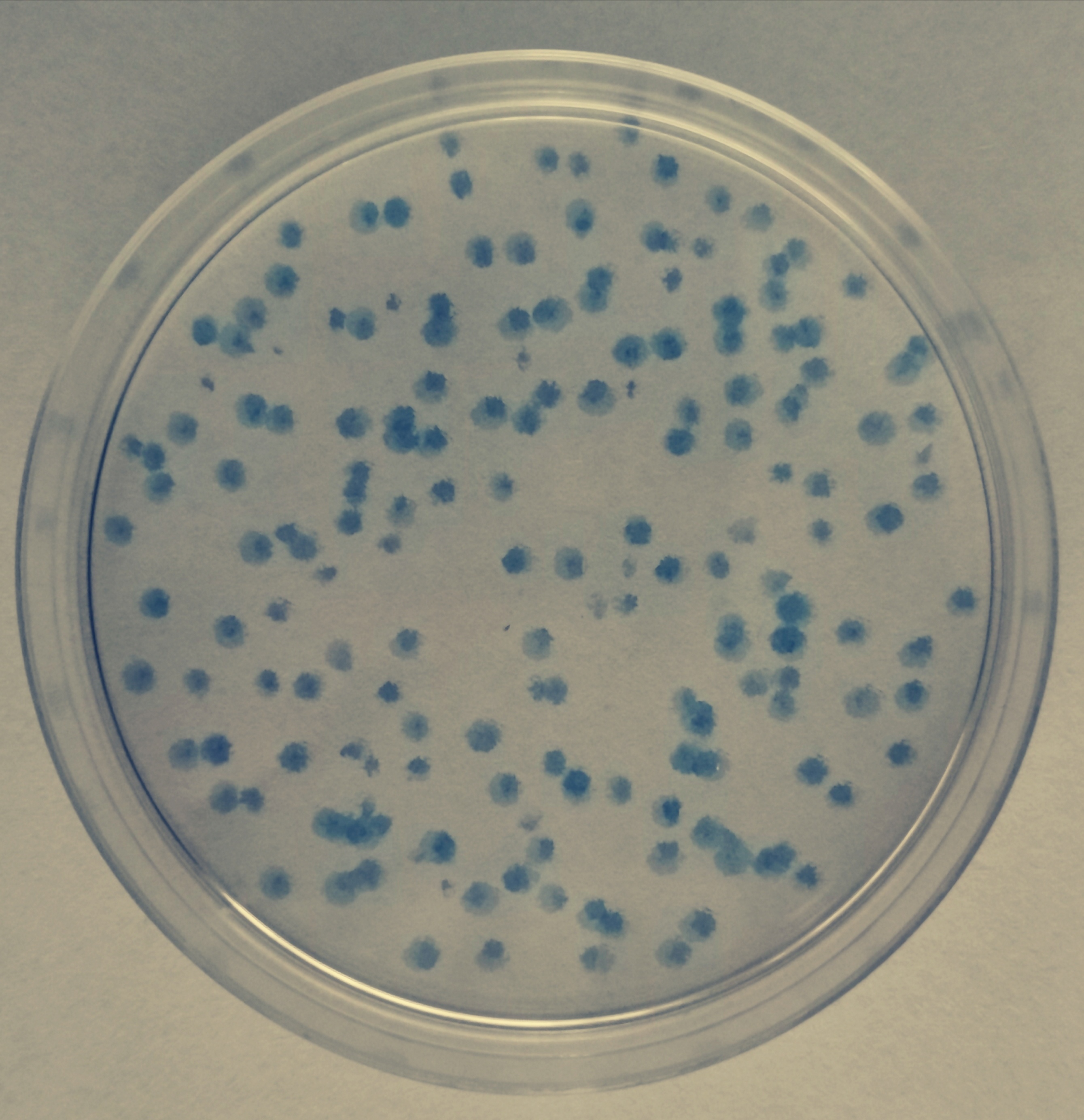

Supplement: Supplementary file 1 [file DataSheet_1.zip › fig2/sw480-colony-3.jpg]

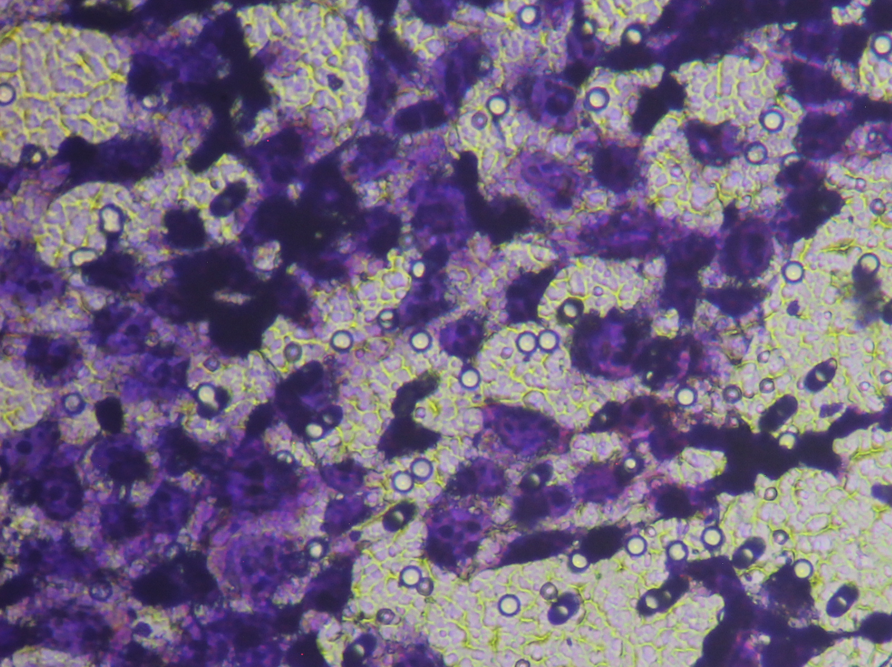

Supplement: Supplementary file 1 [file DataSheet_1.zip › fig2/sw480-invasion-1.png]

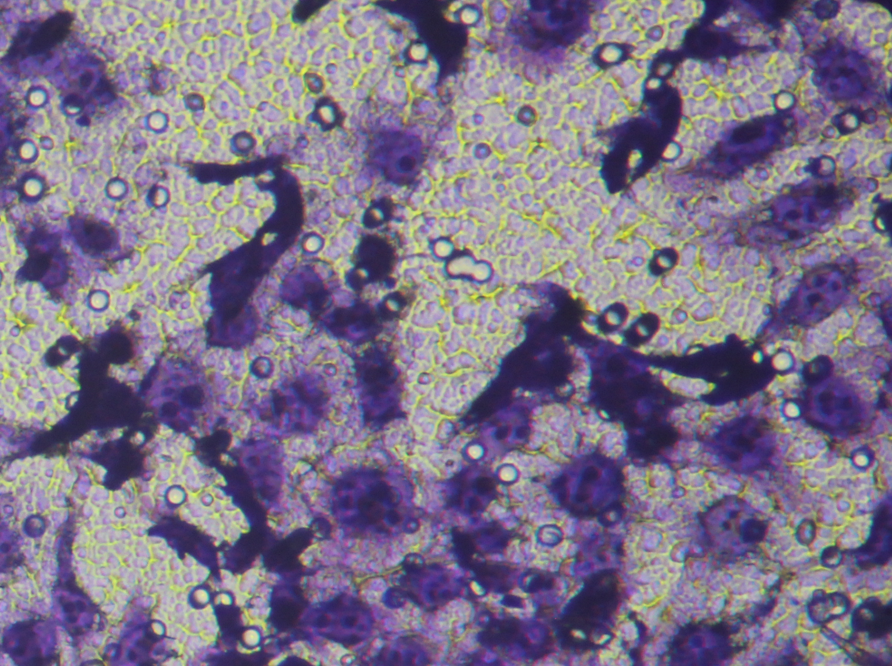

Supplement: Supplementary file 1 [file DataSheet_1.zip › fig2/sw480-invasion-2.png]

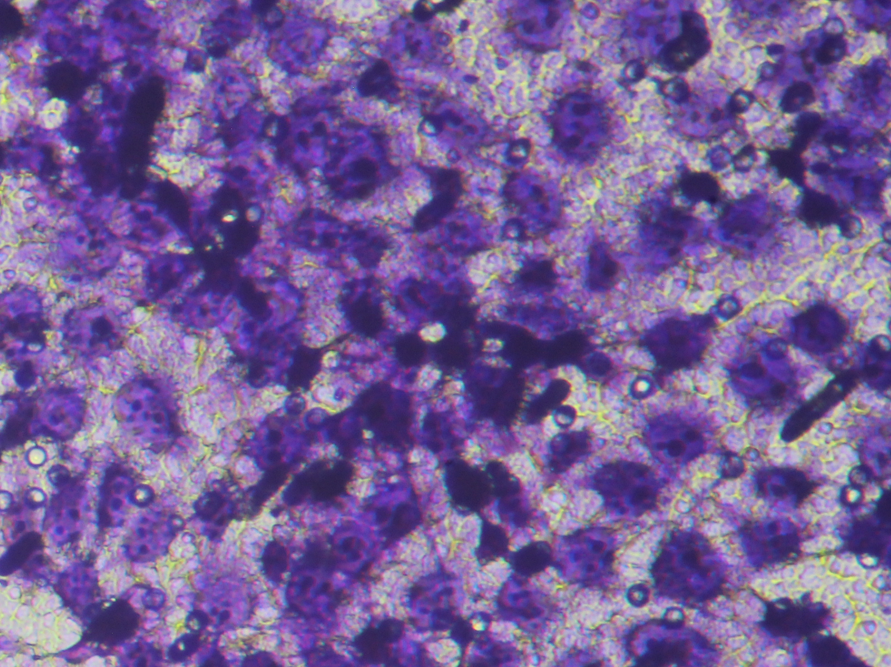

Supplement: Supplementary file 1 [file DataSheet_1.zip › fig2/sw480-invasion-3.png]

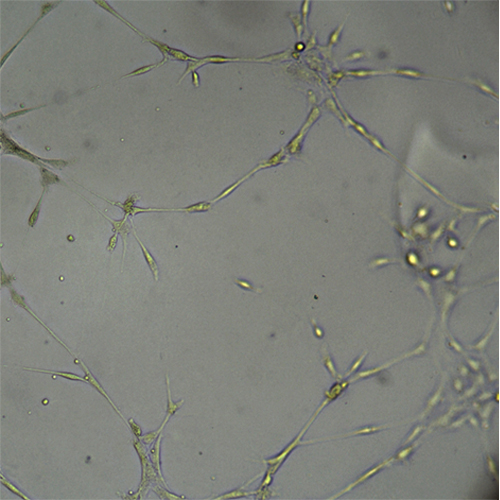

Supplement: Supplementary file 1 [file DataSheet_1.zip › fig2/tube formation-1.jpg]

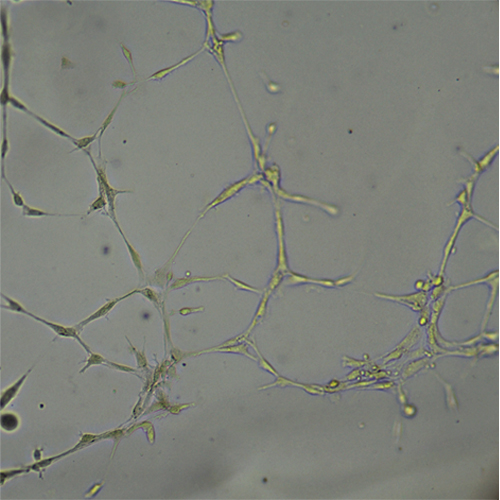

Supplement: Supplementary file 1 [file DataSheet_1.zip › fig2/tube formation-2.jpg]

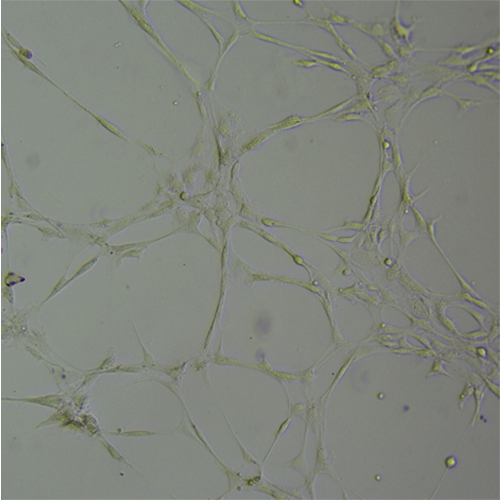

Supplement: Supplementary file 1 [file DataSheet_1.zip › fig2/tube formation-3.jpg]

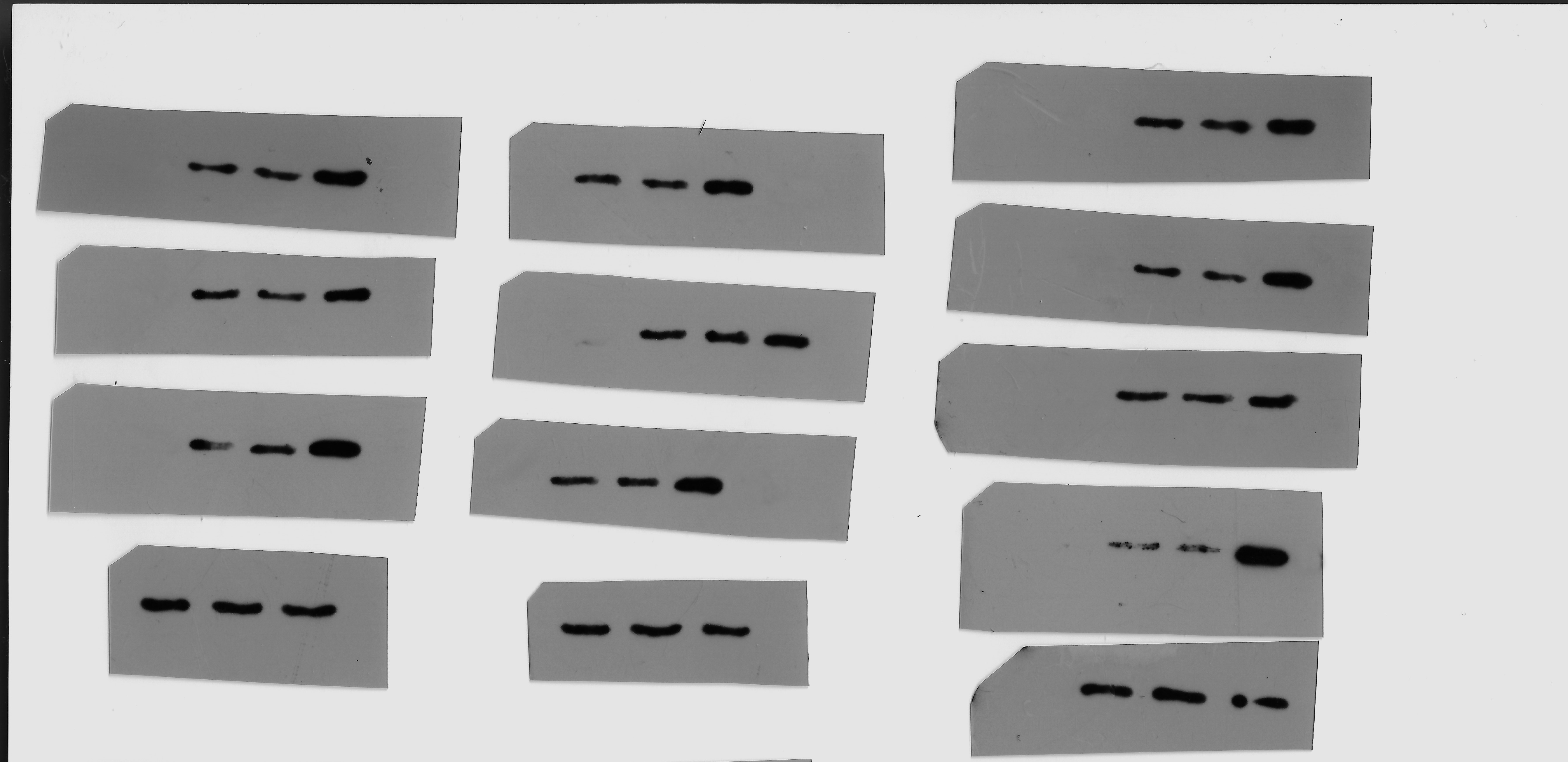

Supplement: Supplementary file 1 [file DataSheet_1.zip › fig2/western blot.jpg]

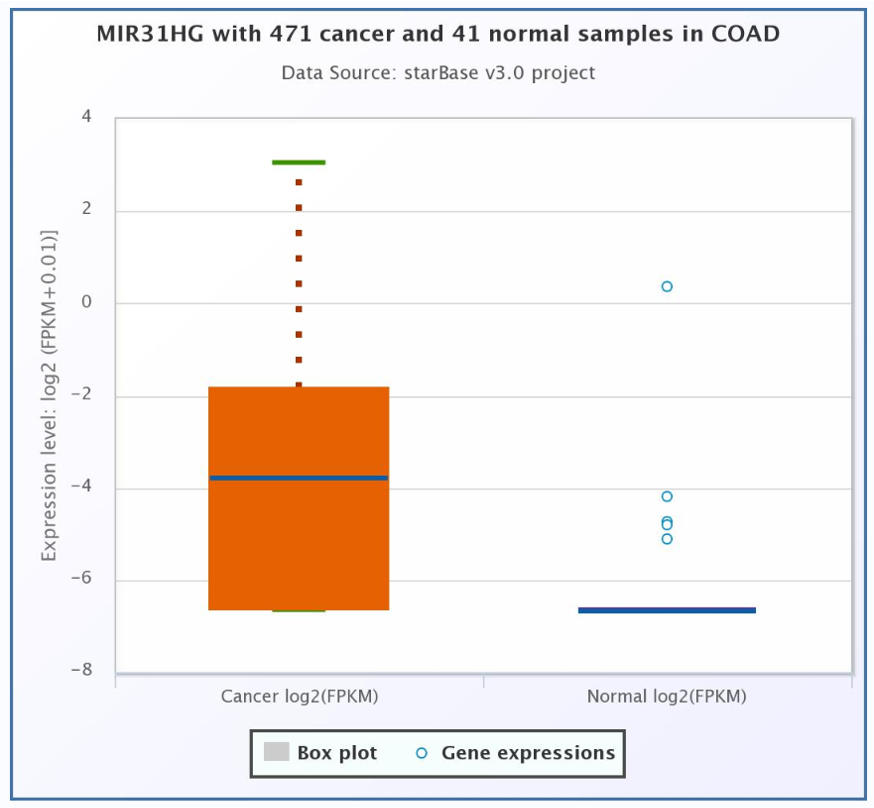

Supplement: Supplementary file 1 [file DataSheet_1.zip › fig1/E.png]

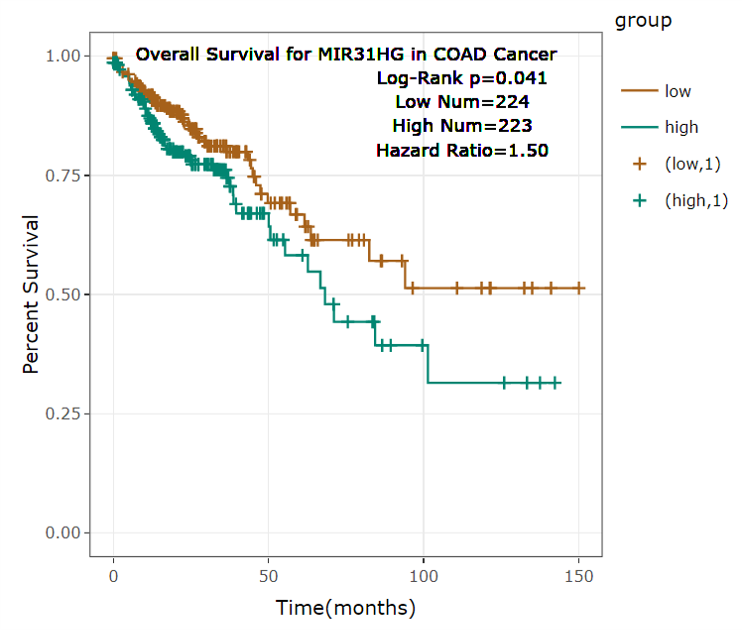

Supplement: Supplementary file 1 [file DataSheet_1.zip › fig1/F.png]

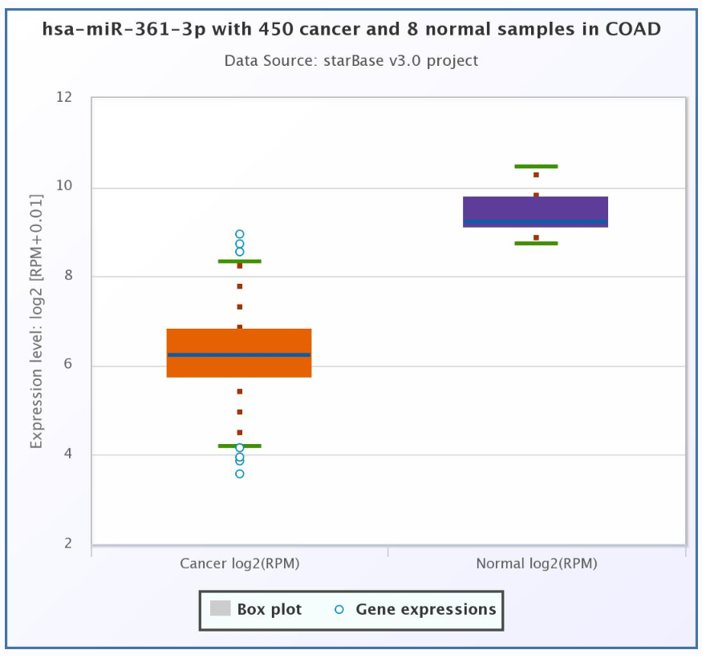

Supplement: Supplementary file 1 [file DataSheet_1.zip › fig1/G.png]

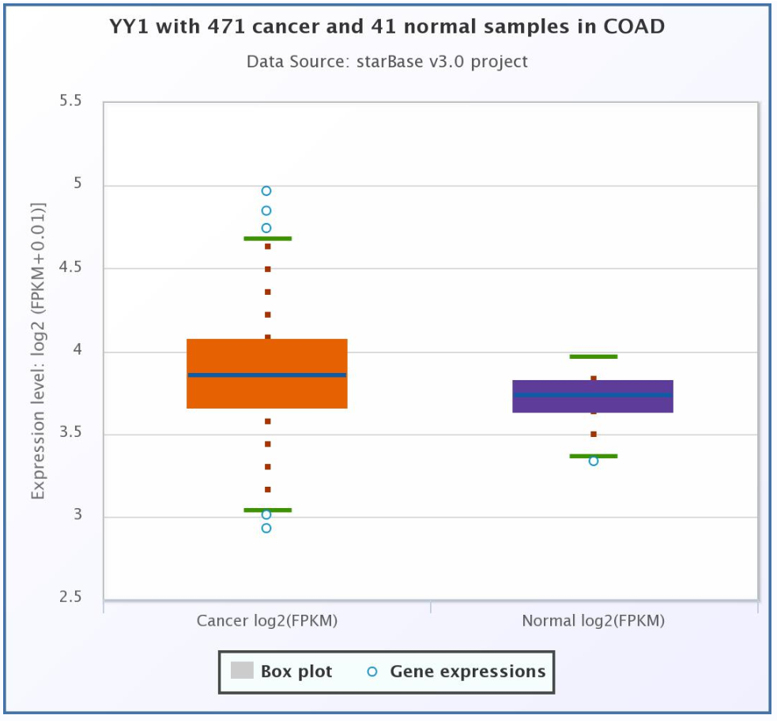

Supplement: Supplementary file 1 [file DataSheet_1.zip › fig1/H.png]

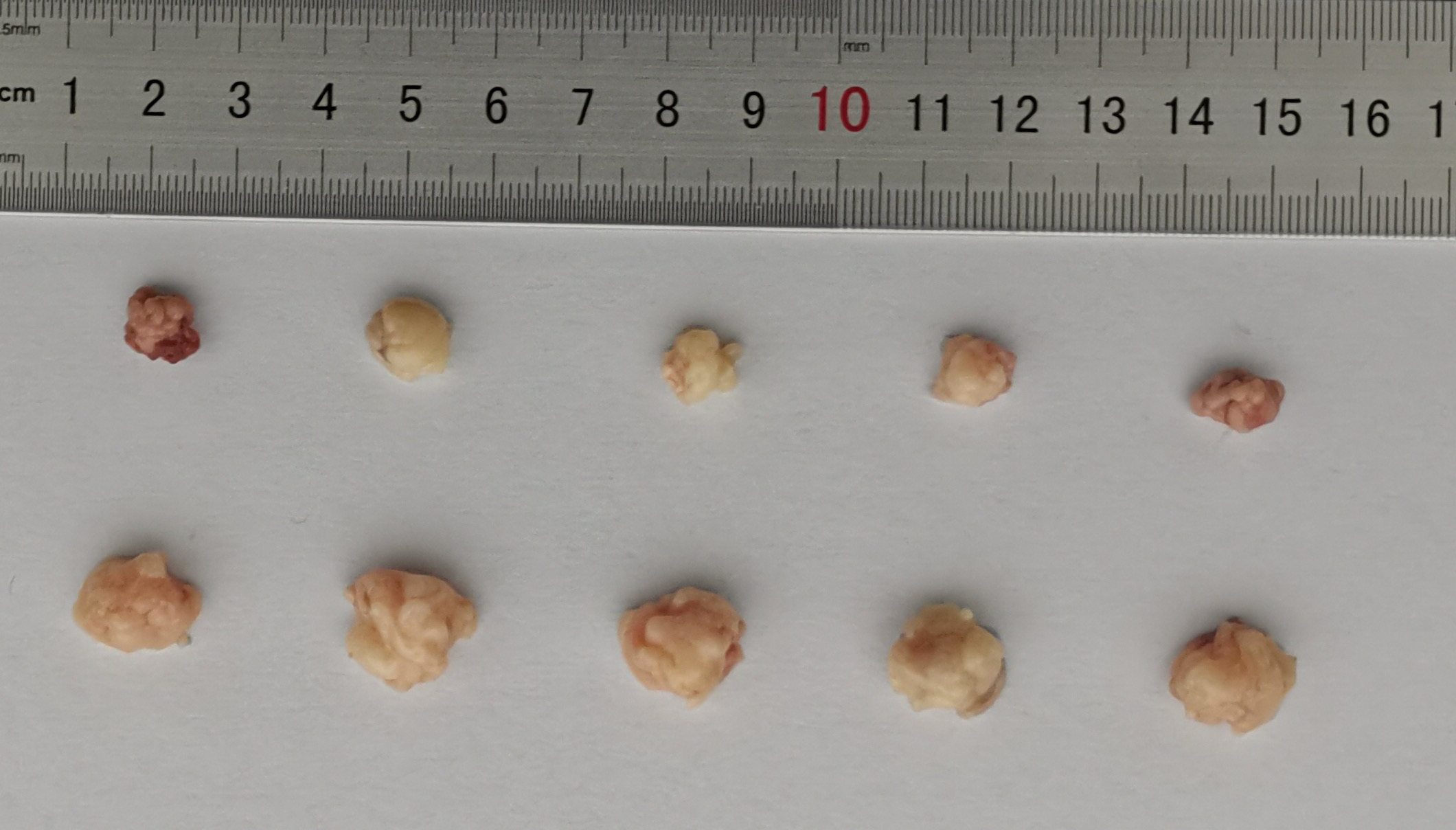

Supplement: Supplementary file 2 [file DataSheet_2.zip › fig4/HCT116.jpg]

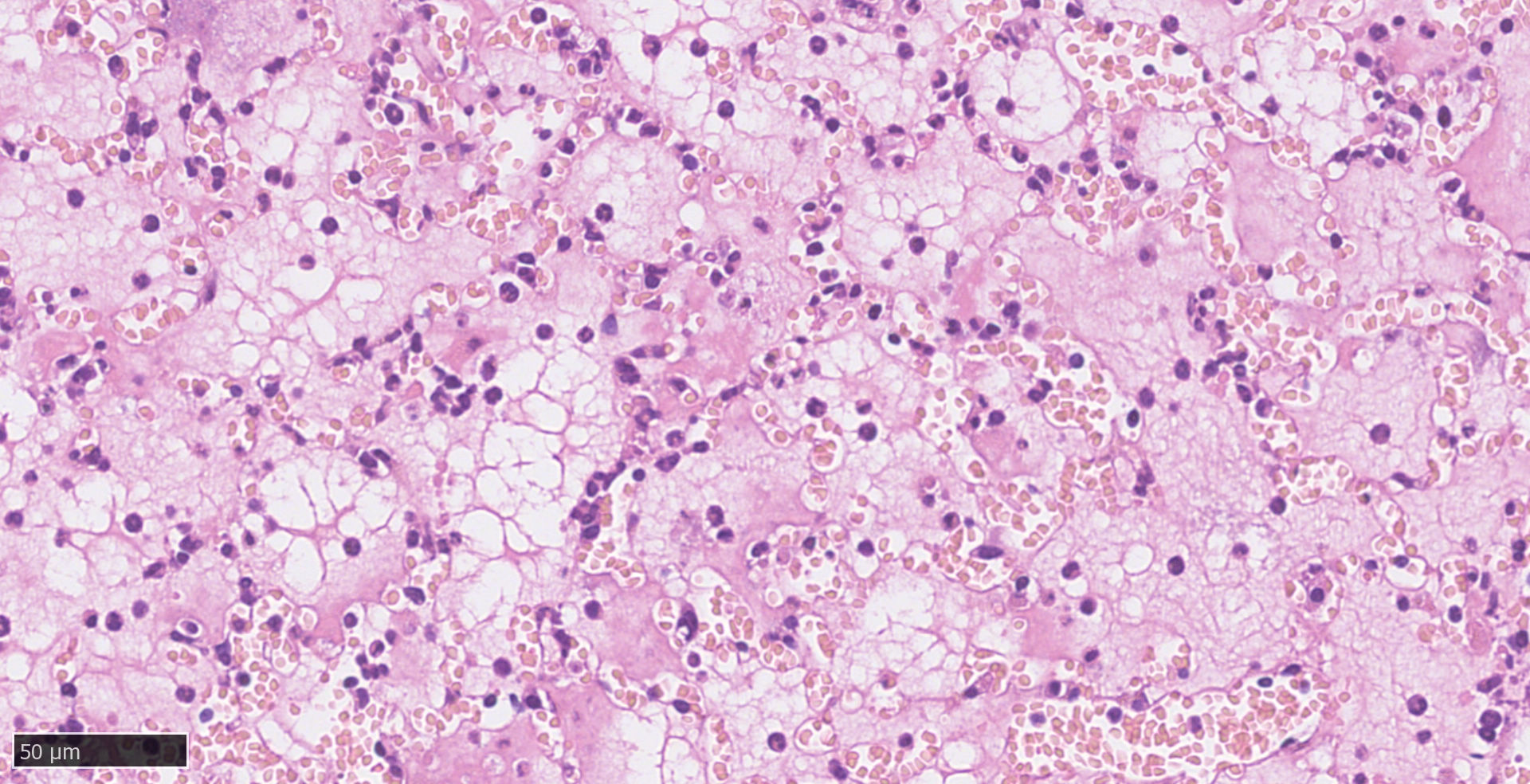

Supplement: Supplementary file 2 [file DataSheet_2.zip › fig4/HE-1-1.jpg]

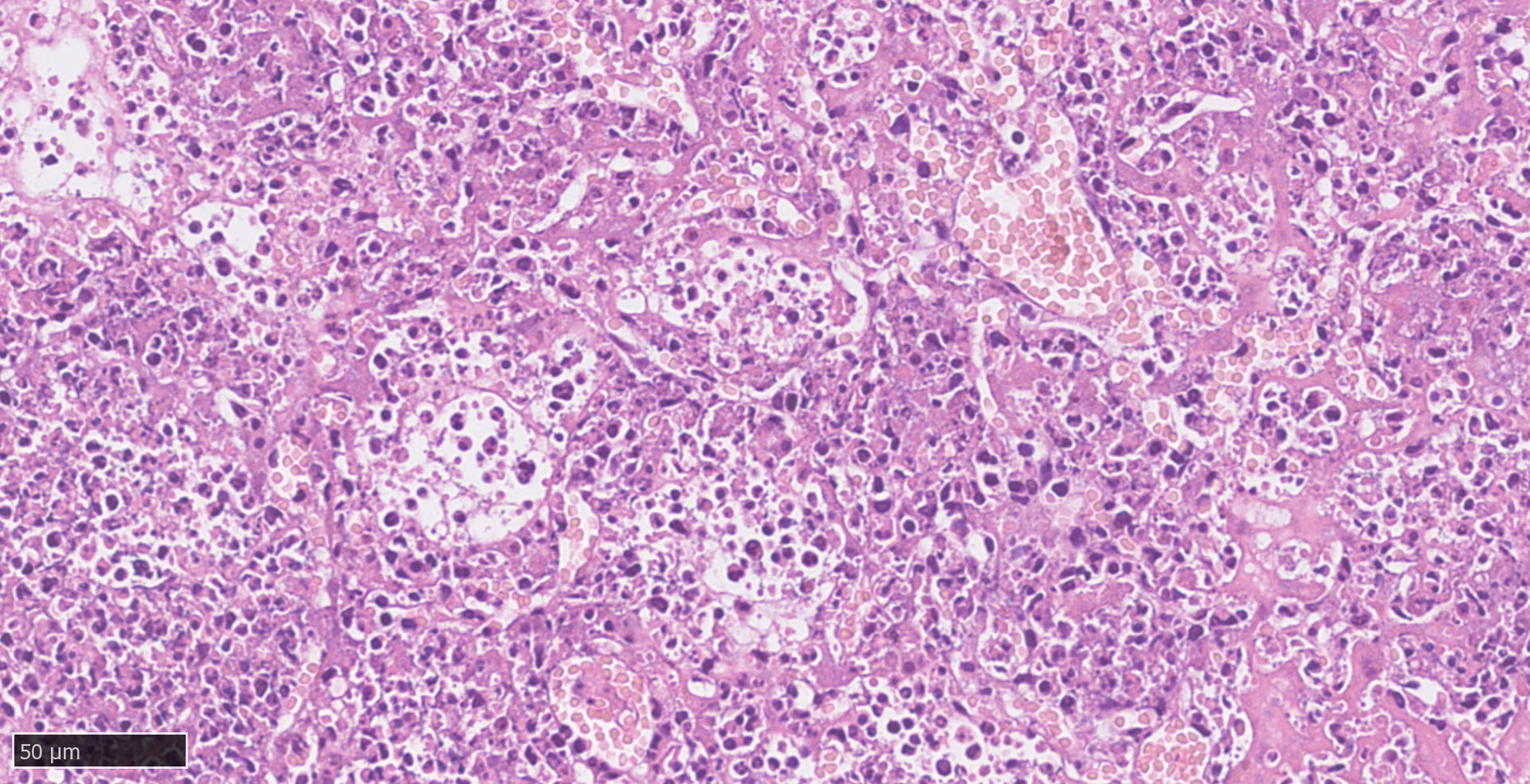

Supplement: Supplementary file 2 [file DataSheet_2.zip › fig4/HE-1-2.jpg]

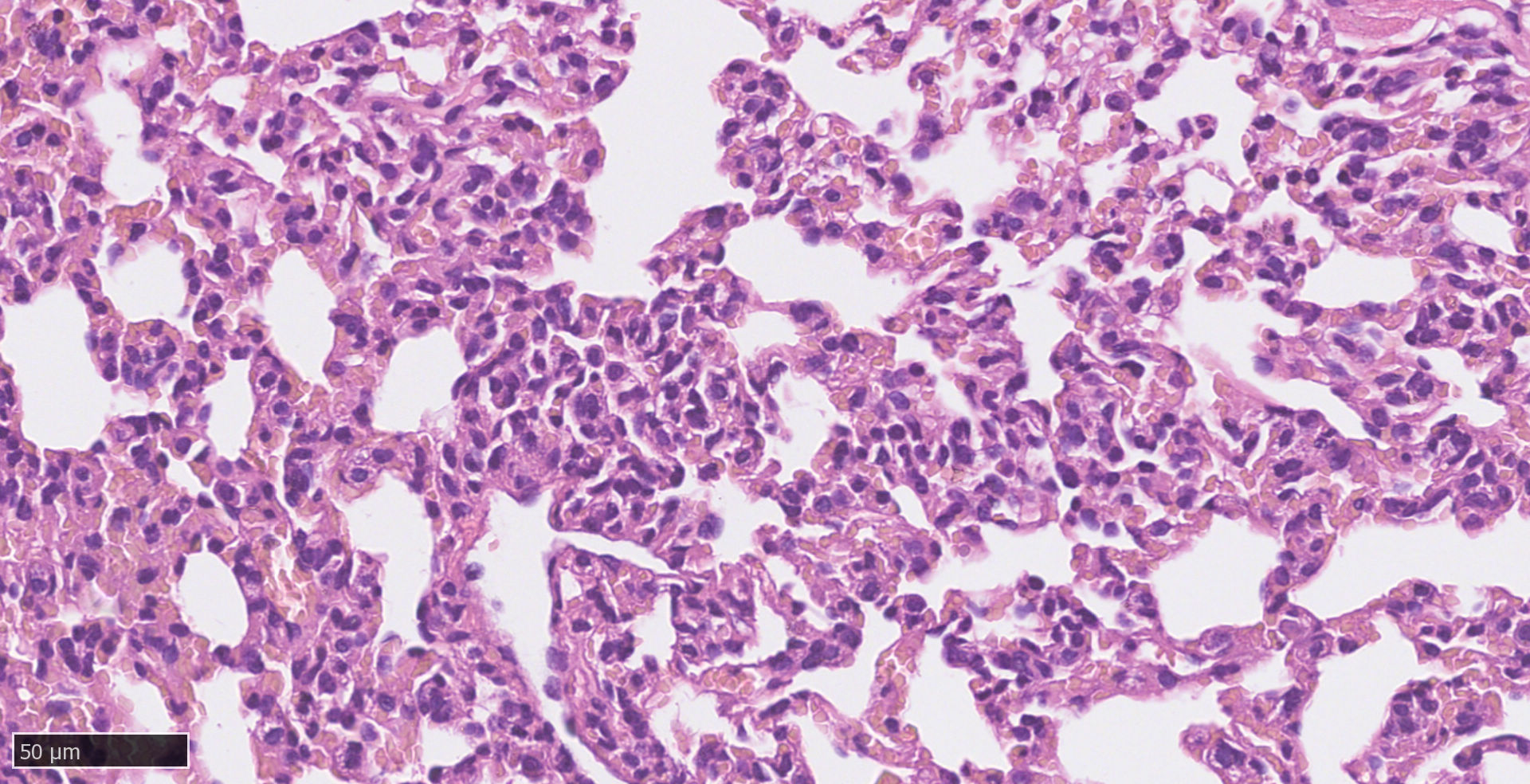

Supplement: Supplementary file 2 [file DataSheet_2.zip › fig4/HE-2-1.jpg]

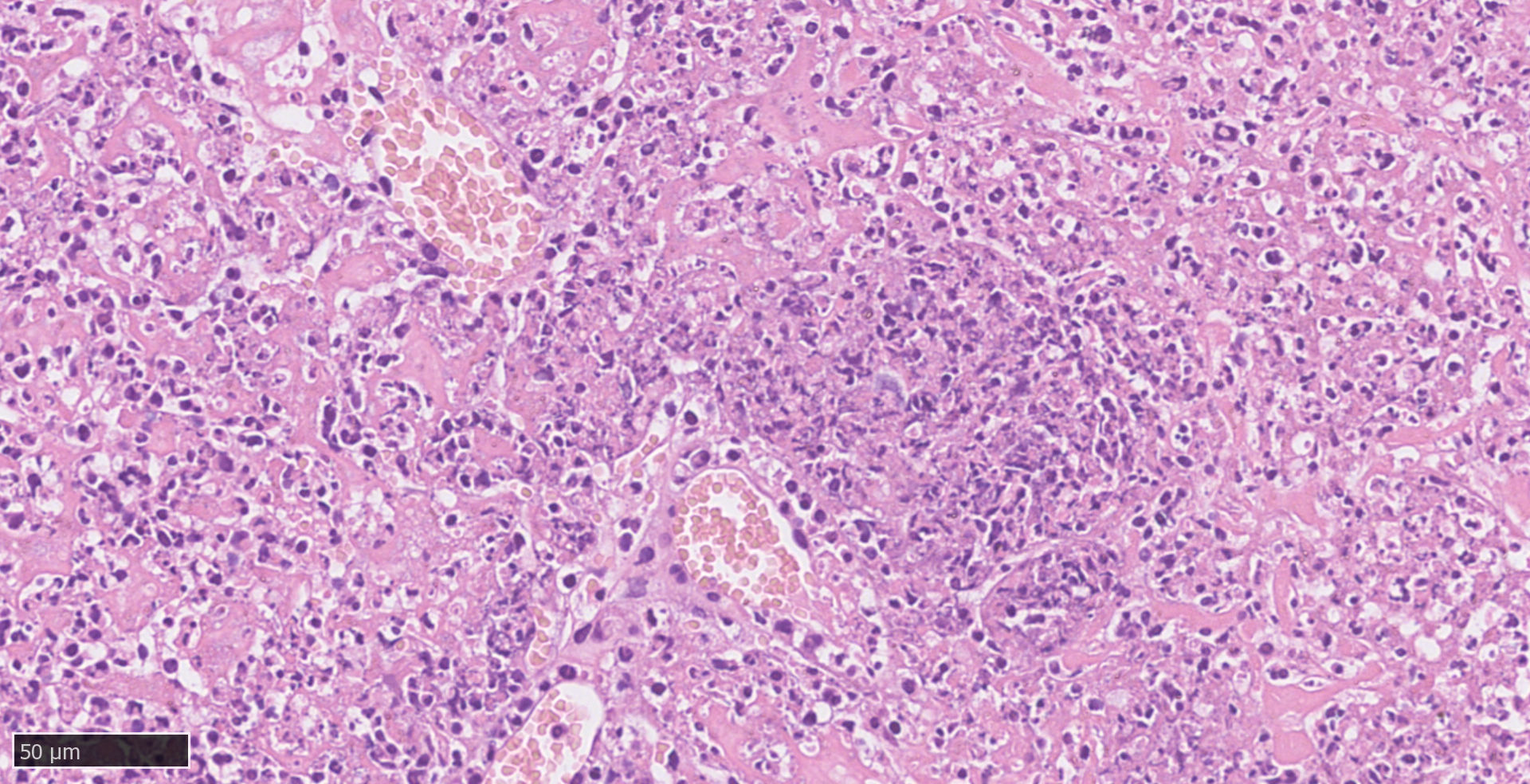

Supplement: Supplementary file 2 [file DataSheet_2.zip › fig4/HE-2-2.jpg]

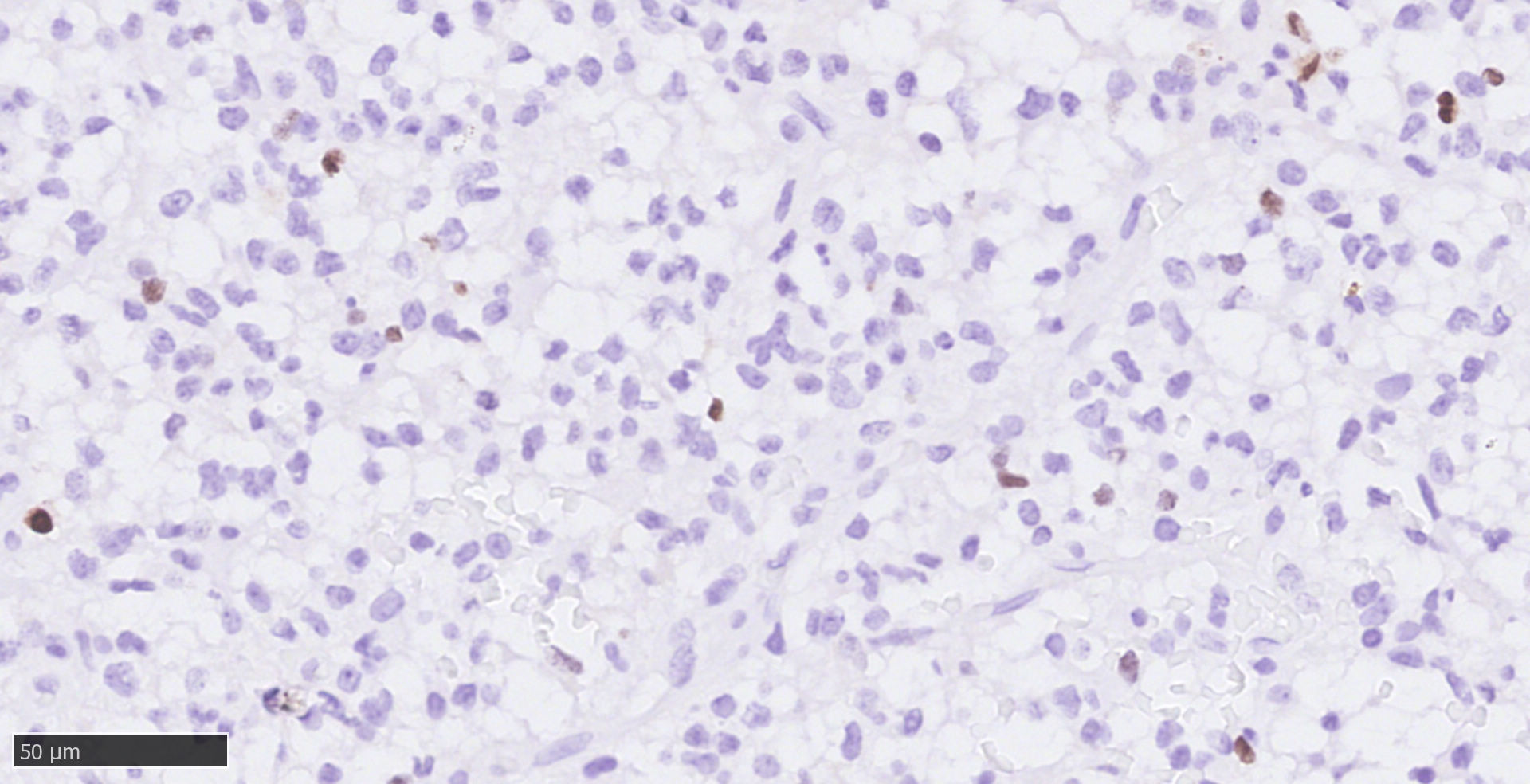

Supplement: Supplementary file 2 [file DataSheet_2.zip › fig4/KI67-1-1.jpg]

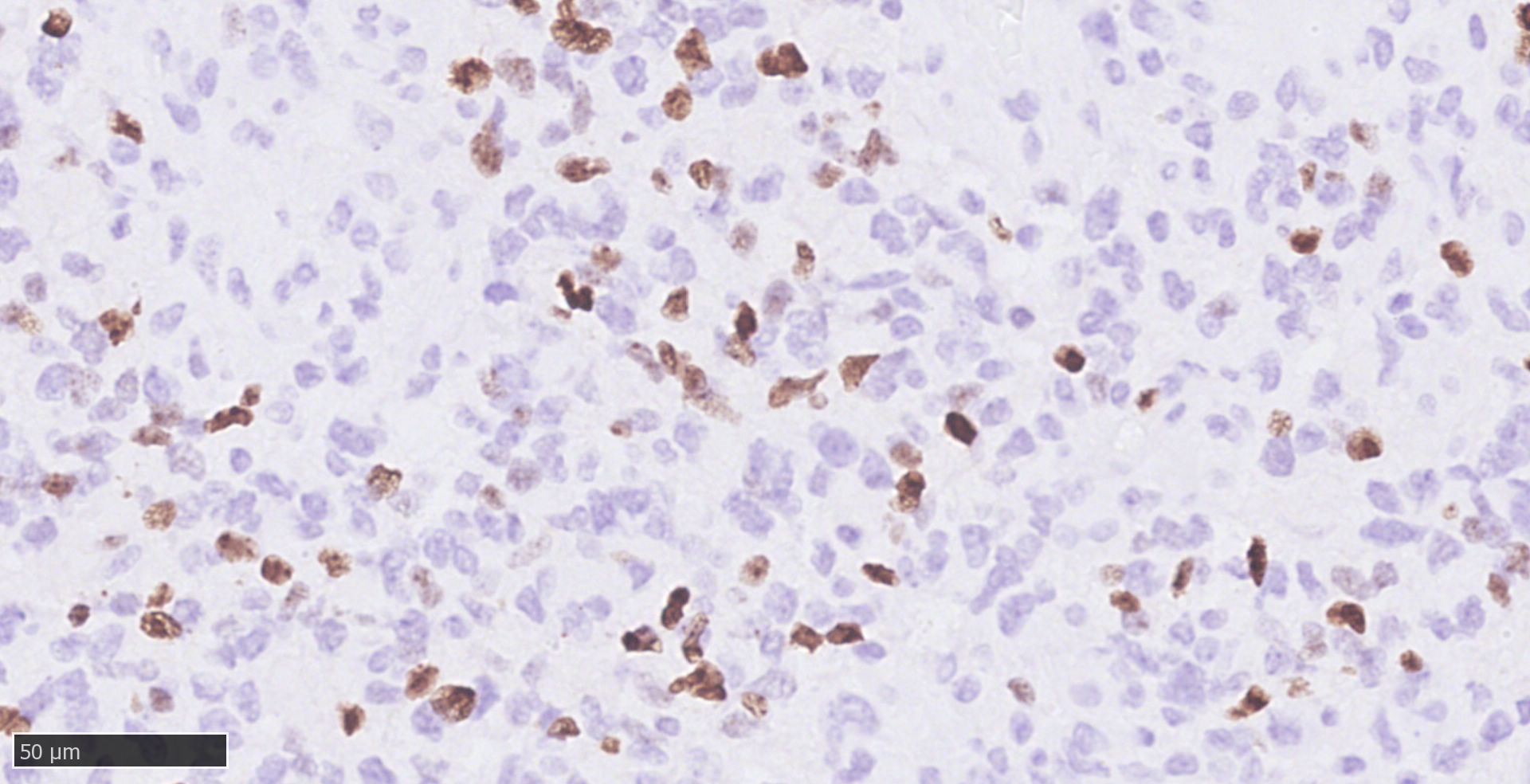

Supplement: Supplementary file 2 [file DataSheet_2.zip › fig4/KI67-1-2.jpg]

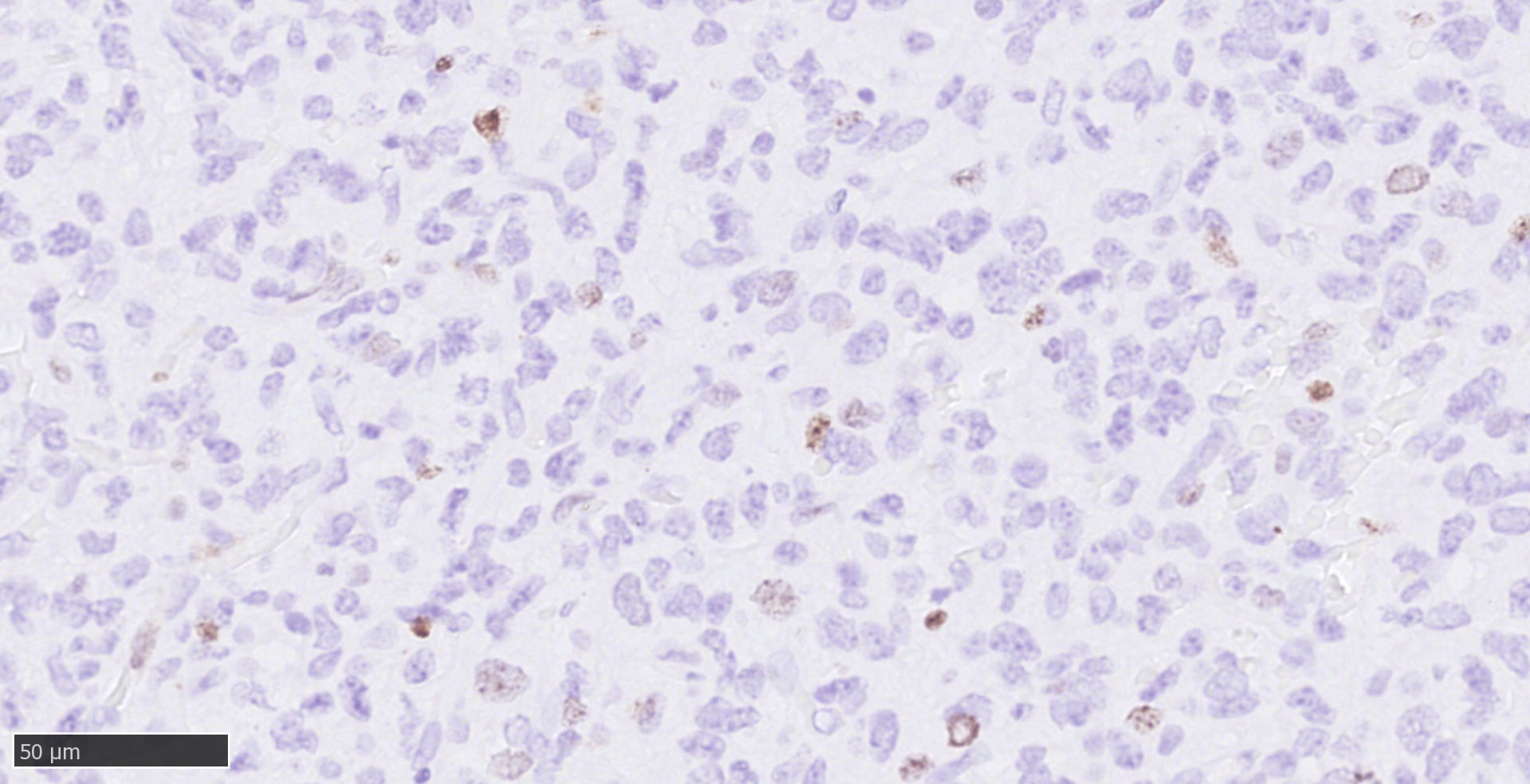

Supplement: Supplementary file 2 [file DataSheet_2.zip › fig4/KI67-2-1.jpg]

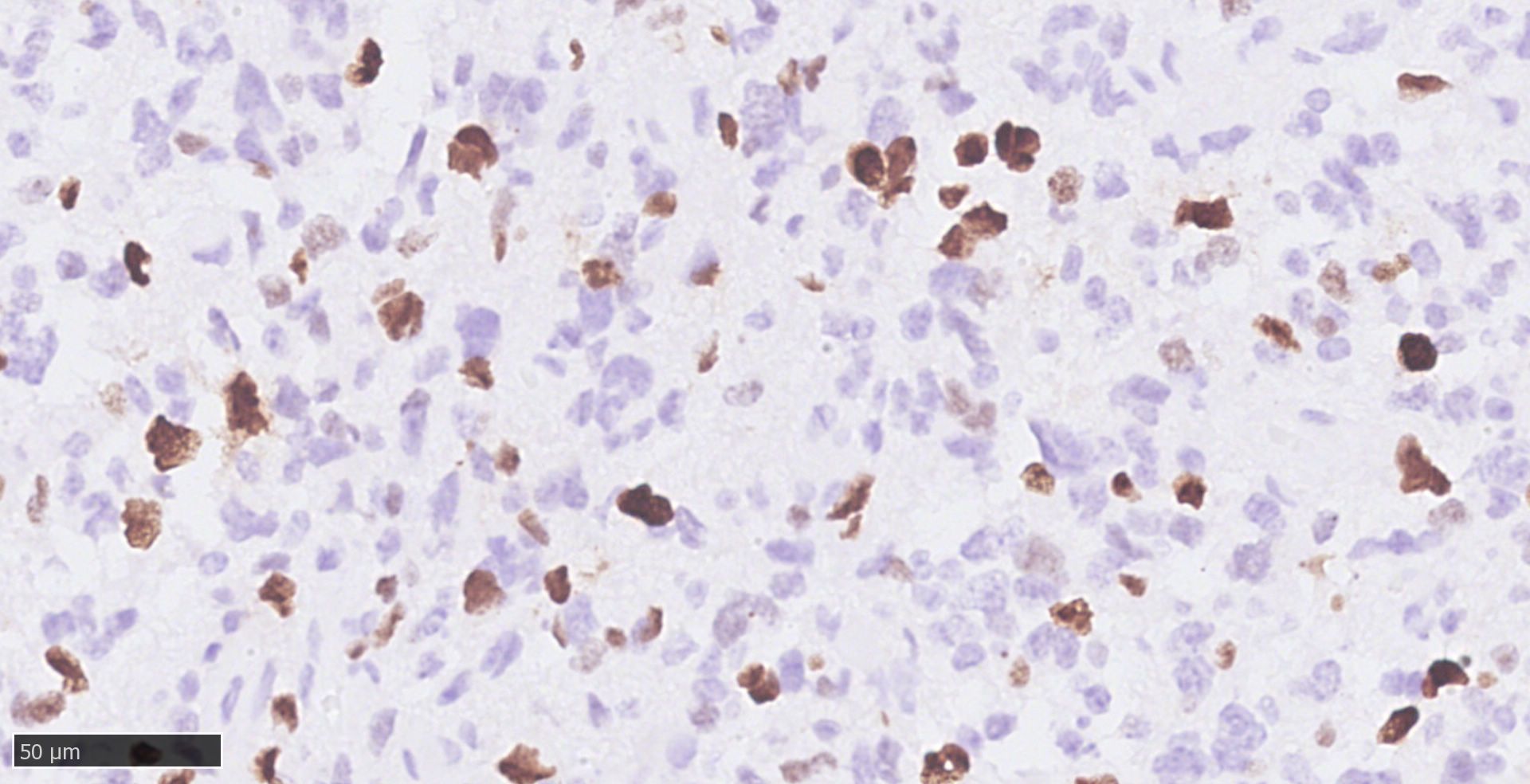

Supplement: Supplementary file 2 [file DataSheet_2.zip › fig4/KI67-2-2.jpg]

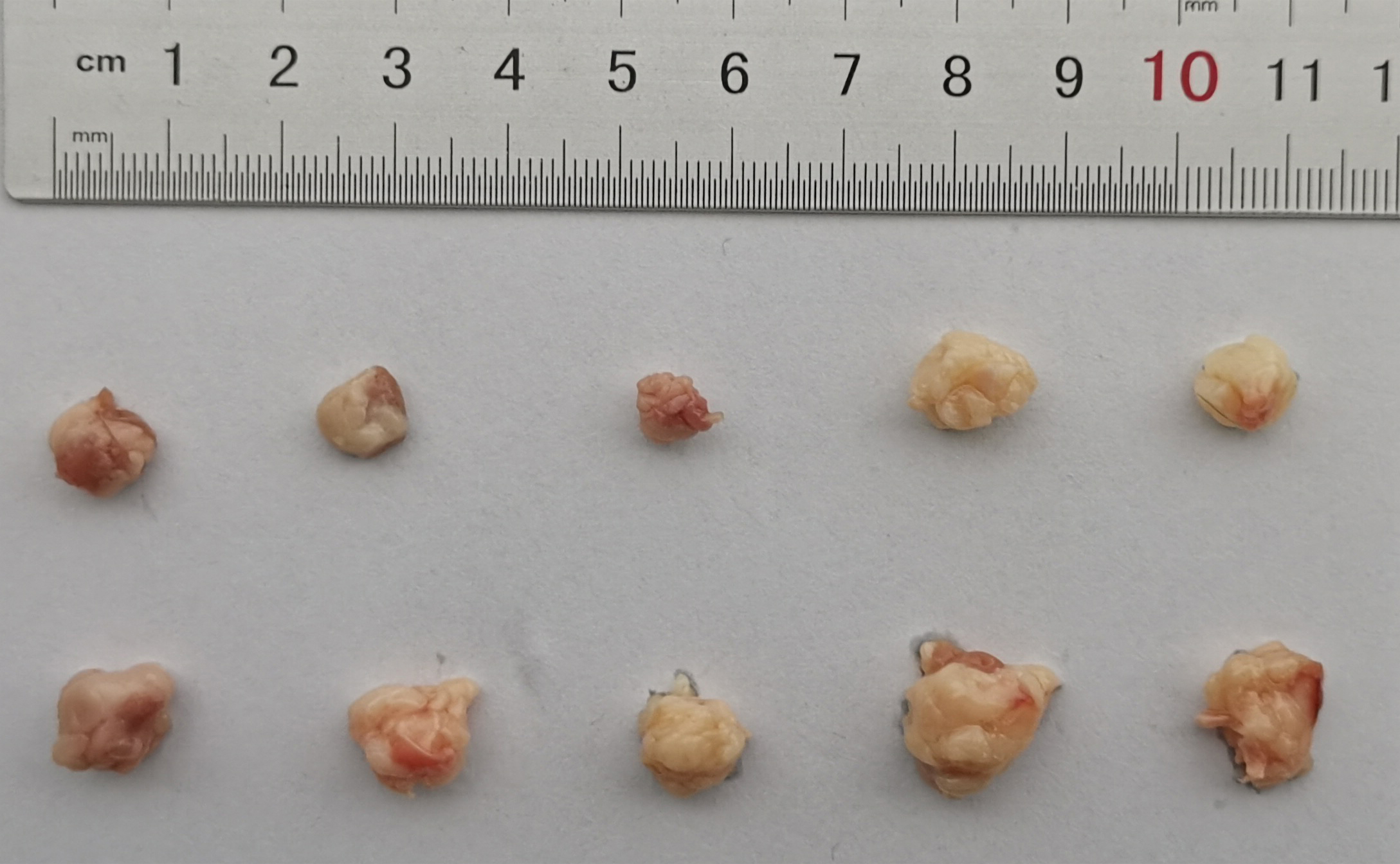

Supplement: Supplementary file 2 [file DataSheet_2.zip › fig4/SW480.jpg]

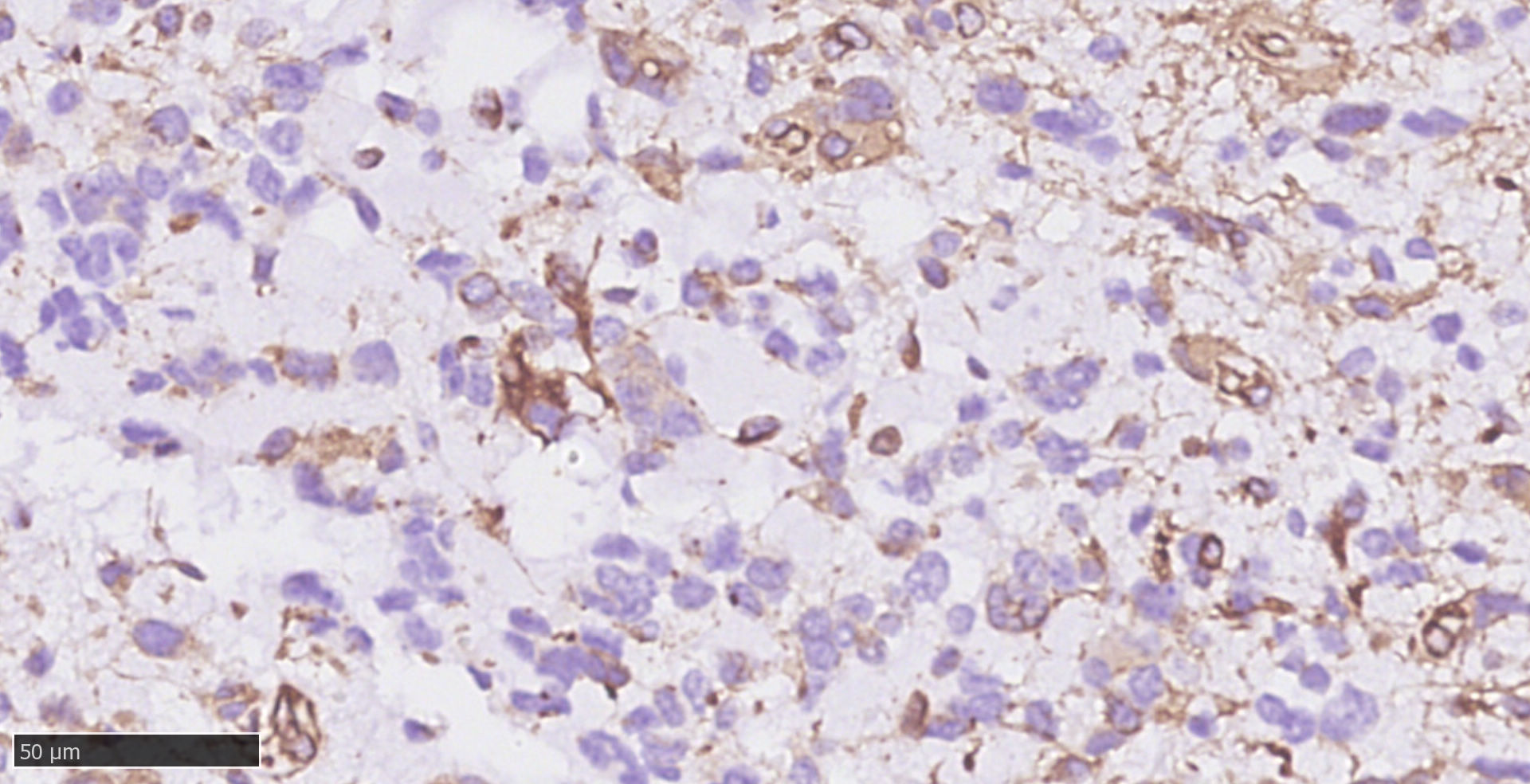

Supplement: Supplementary file 2 [file DataSheet_2.zip › fig4/VEGFA-1-1.jpg]

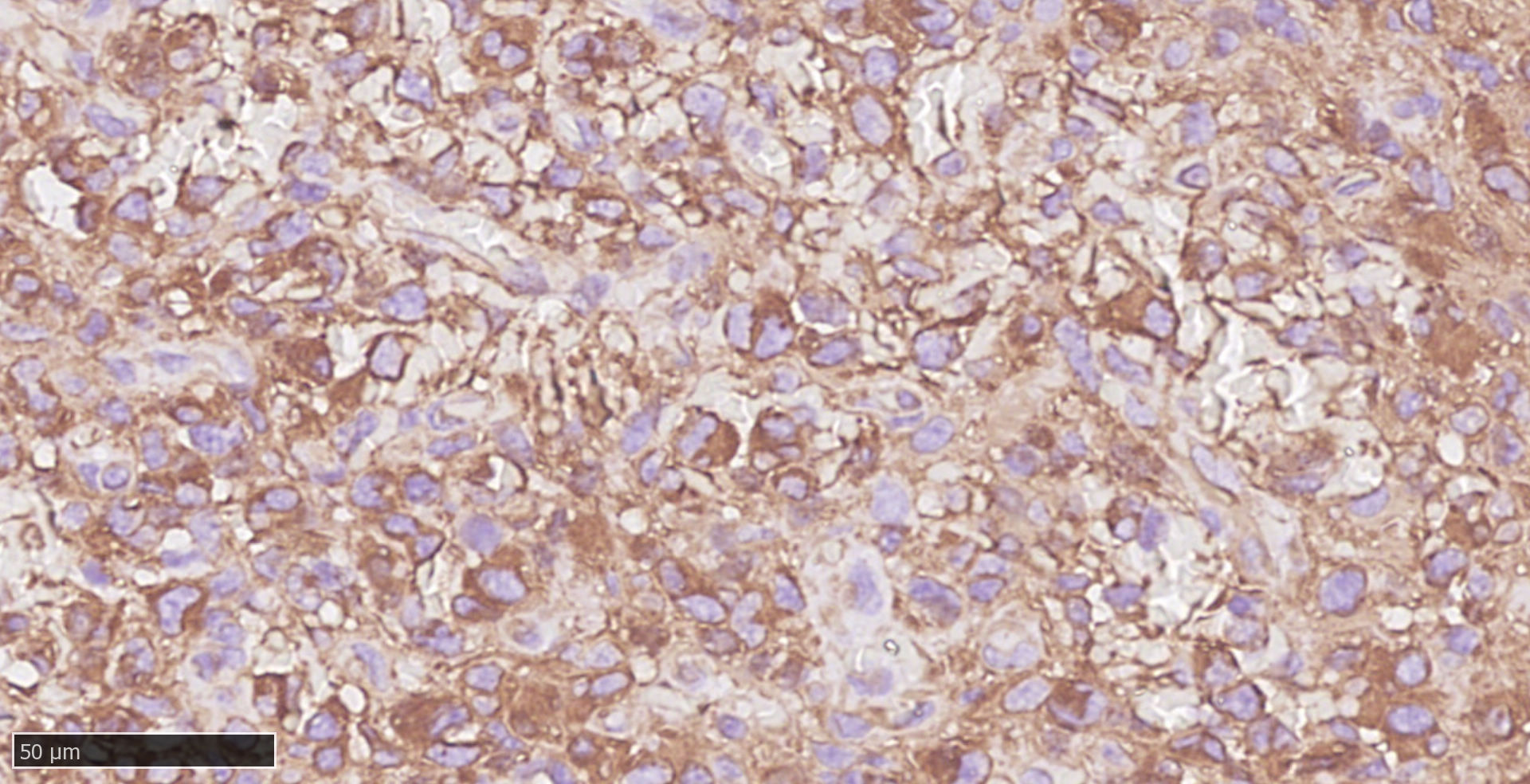

Supplement: Supplementary file 2 [file DataSheet_2.zip › fig4/VEGFA-1-2.jpg]

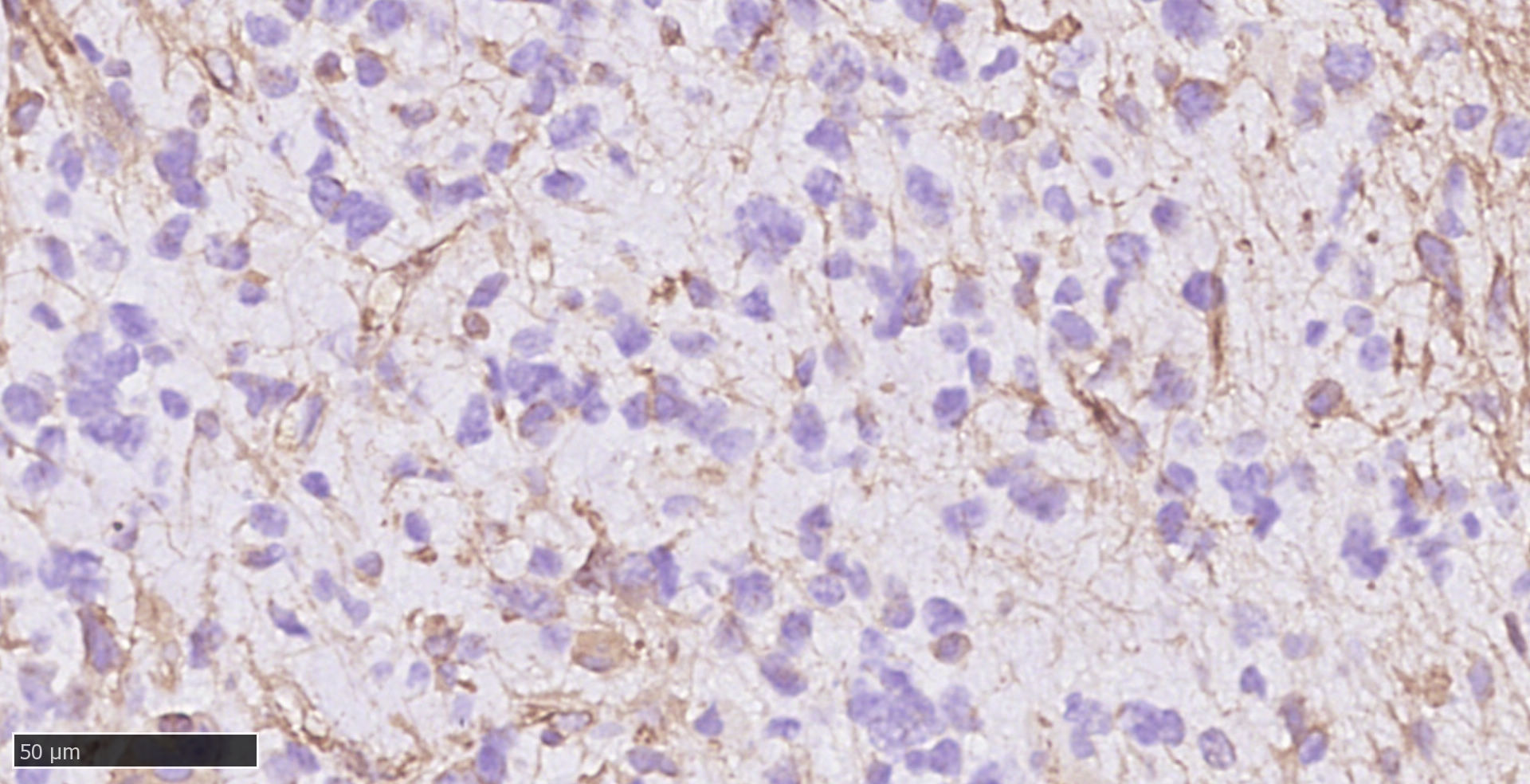

Supplement: Supplementary file 2 [file DataSheet_2.zip › fig4/VEGFA-2-1.jpg]

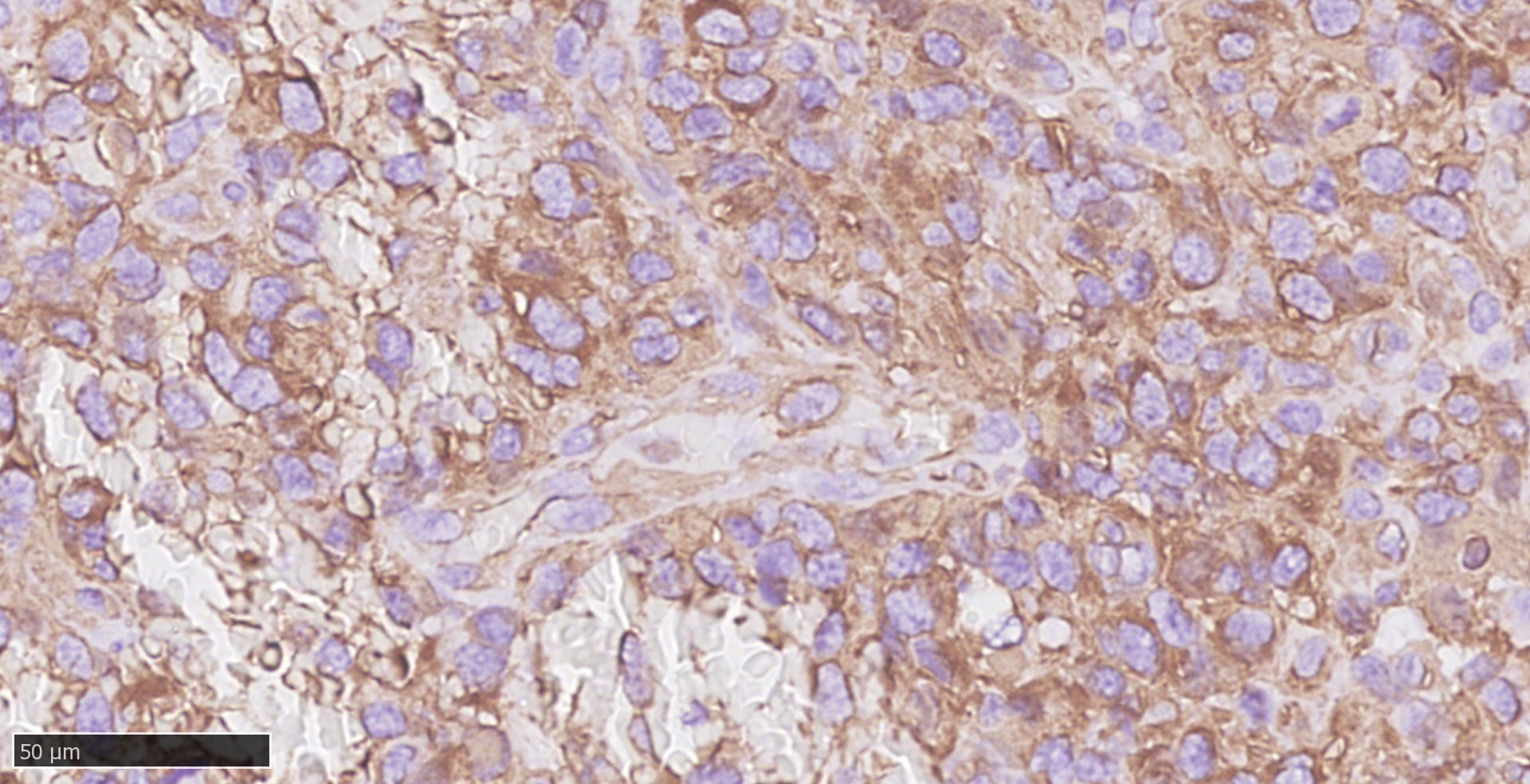

Supplement: Supplementary file 2 [file DataSheet_2.zip › fig4/VEGFA-2-2.jpg]

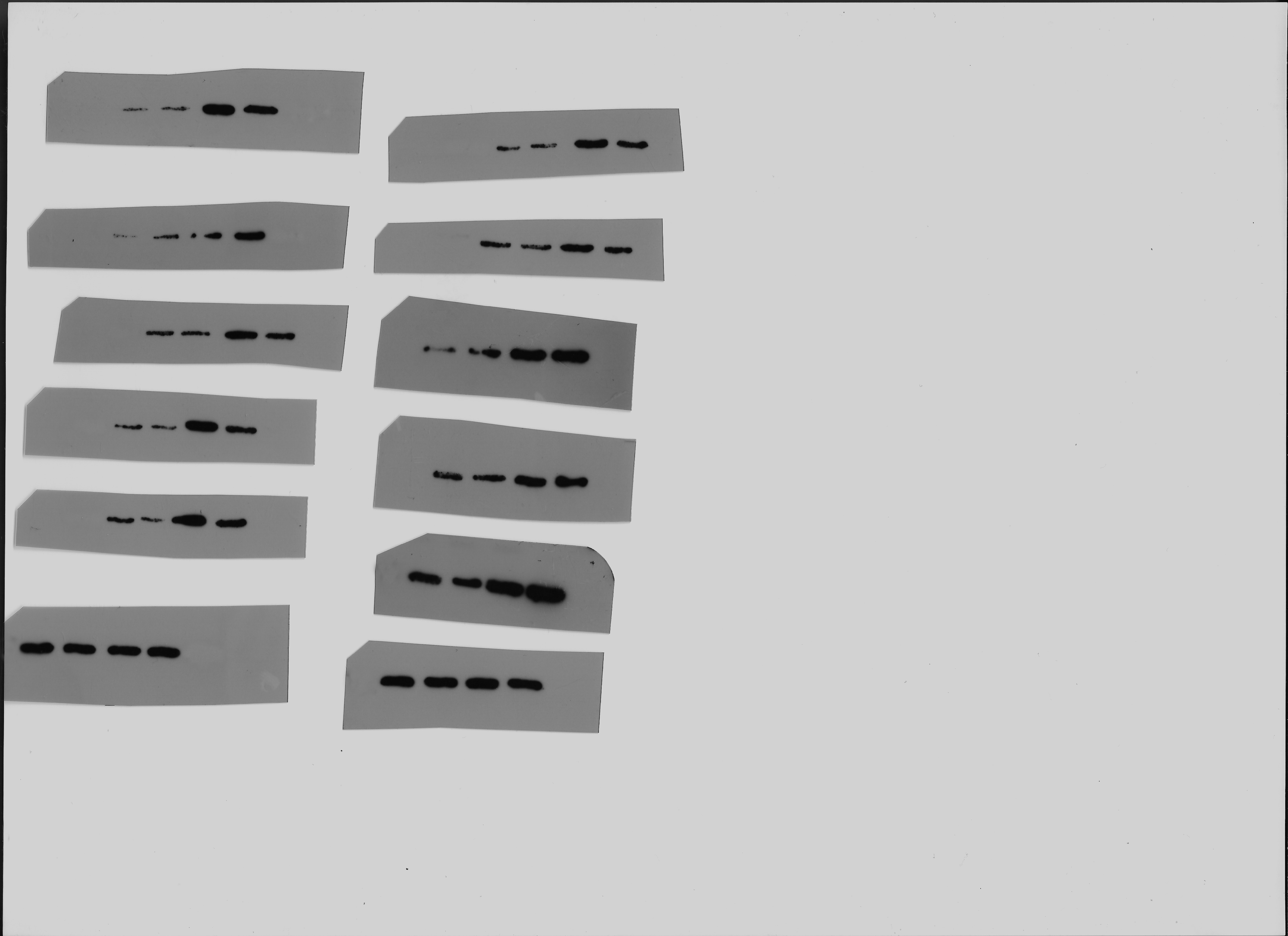

Supplement: Supplementary file 2 [file DataSheet_2.zip › fig4/western blot.jpg]

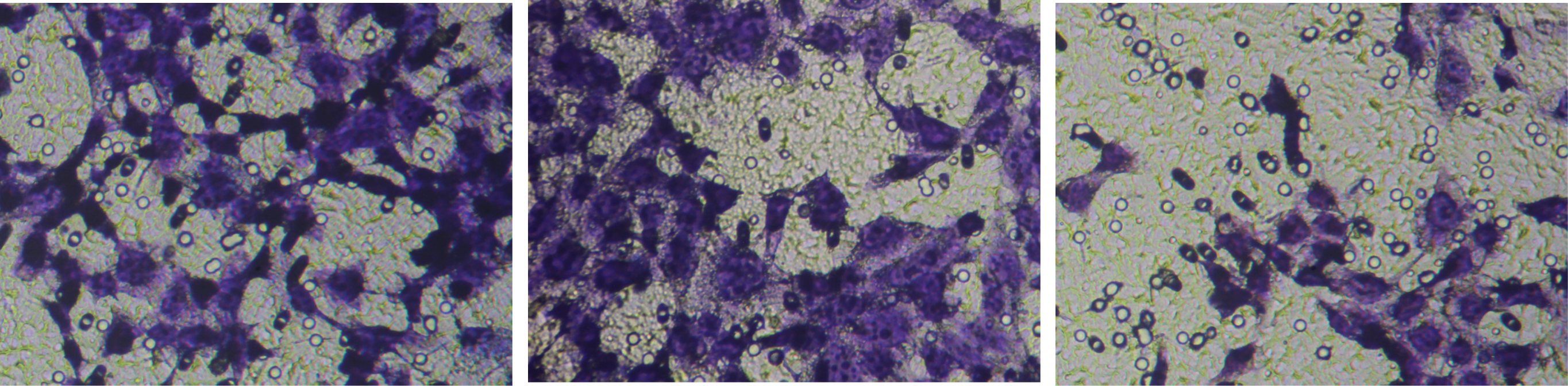

Supplement: Supplementary file 2 [file DataSheet_2.zip › fig3/-+¦1⁄42.png]

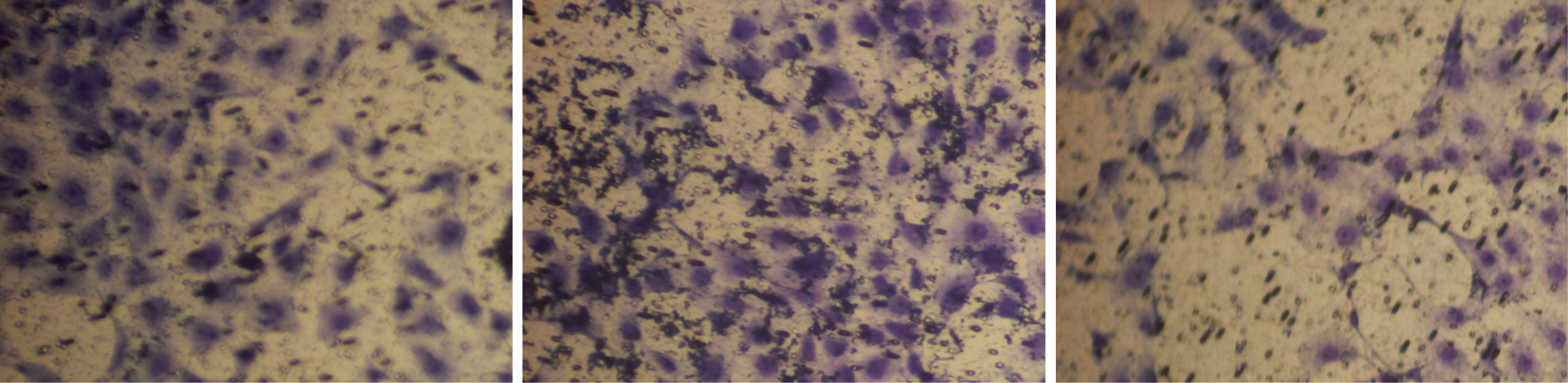

Supplement: Supplementary file 2 [file DataSheet_2.zip › fig3/-+¦1⁄46.png]

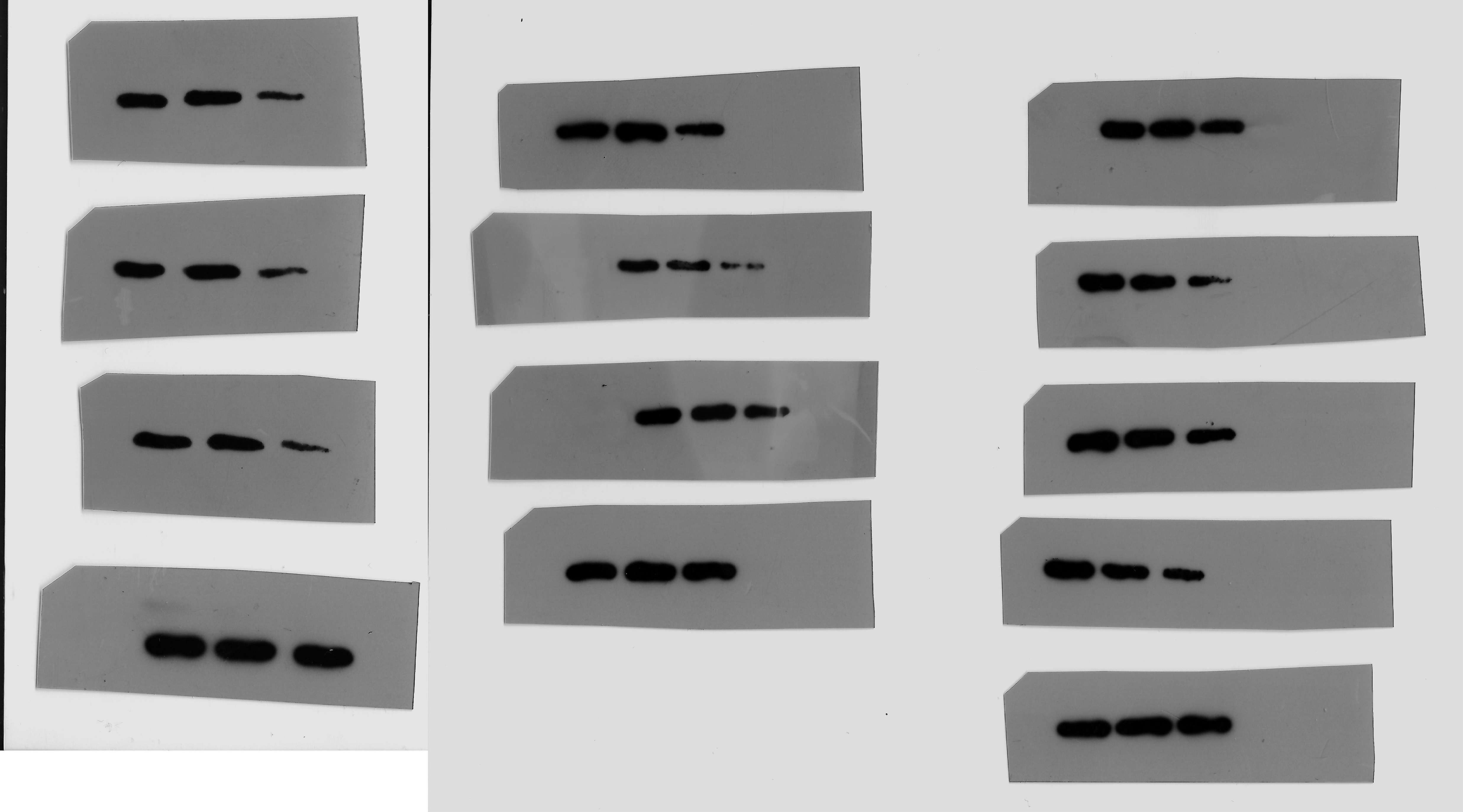

Supplement: Supplementary file 2 [file DataSheet_2.zip › fig3/684984_western blot.tif]

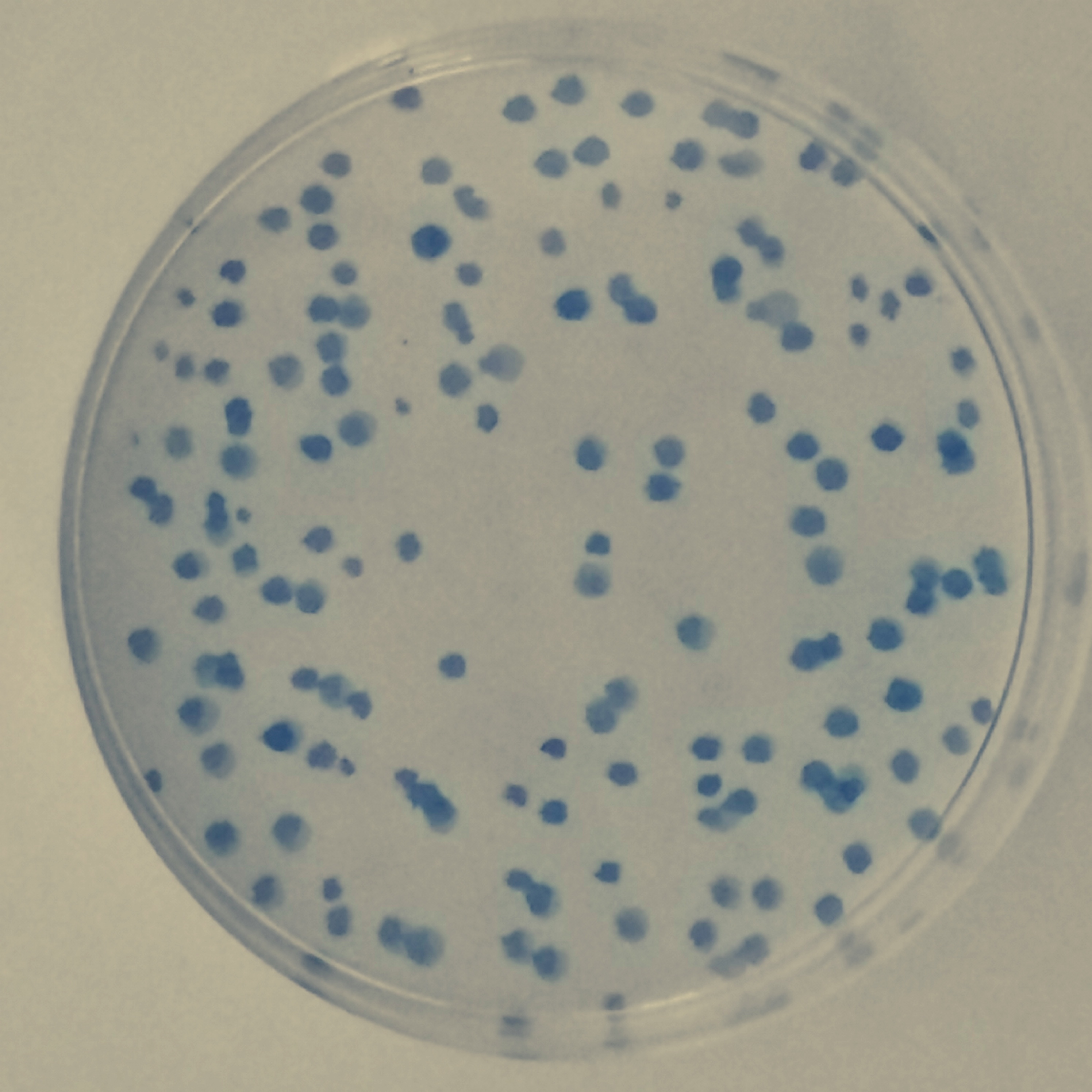

Supplement: Supplementary file 2 [file DataSheet_2.zip › fig3/colony-HCT116-1.jpg]

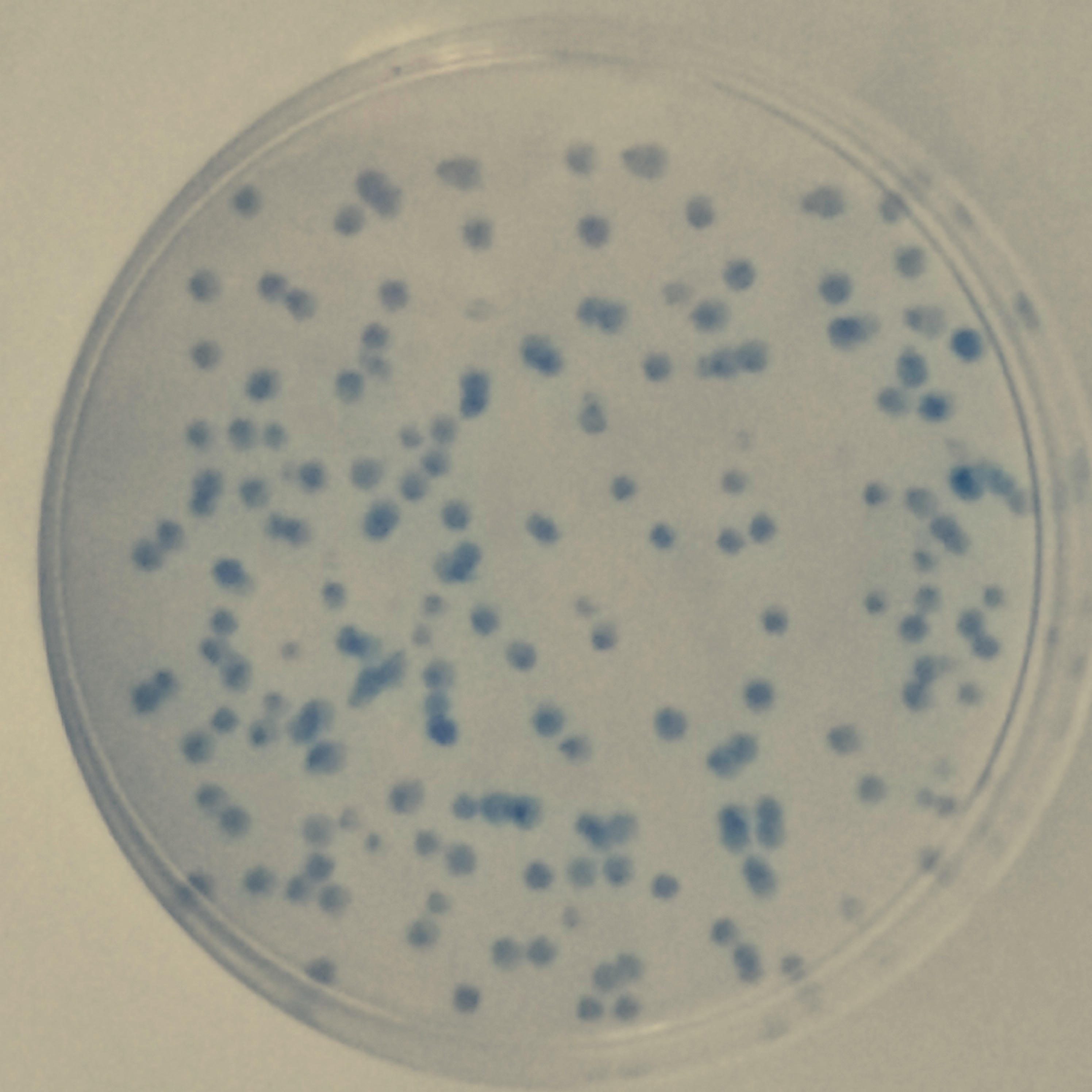

Supplement: Supplementary file 2 [file DataSheet_2.zip › fig3/colony-HCT116-2.jpg]

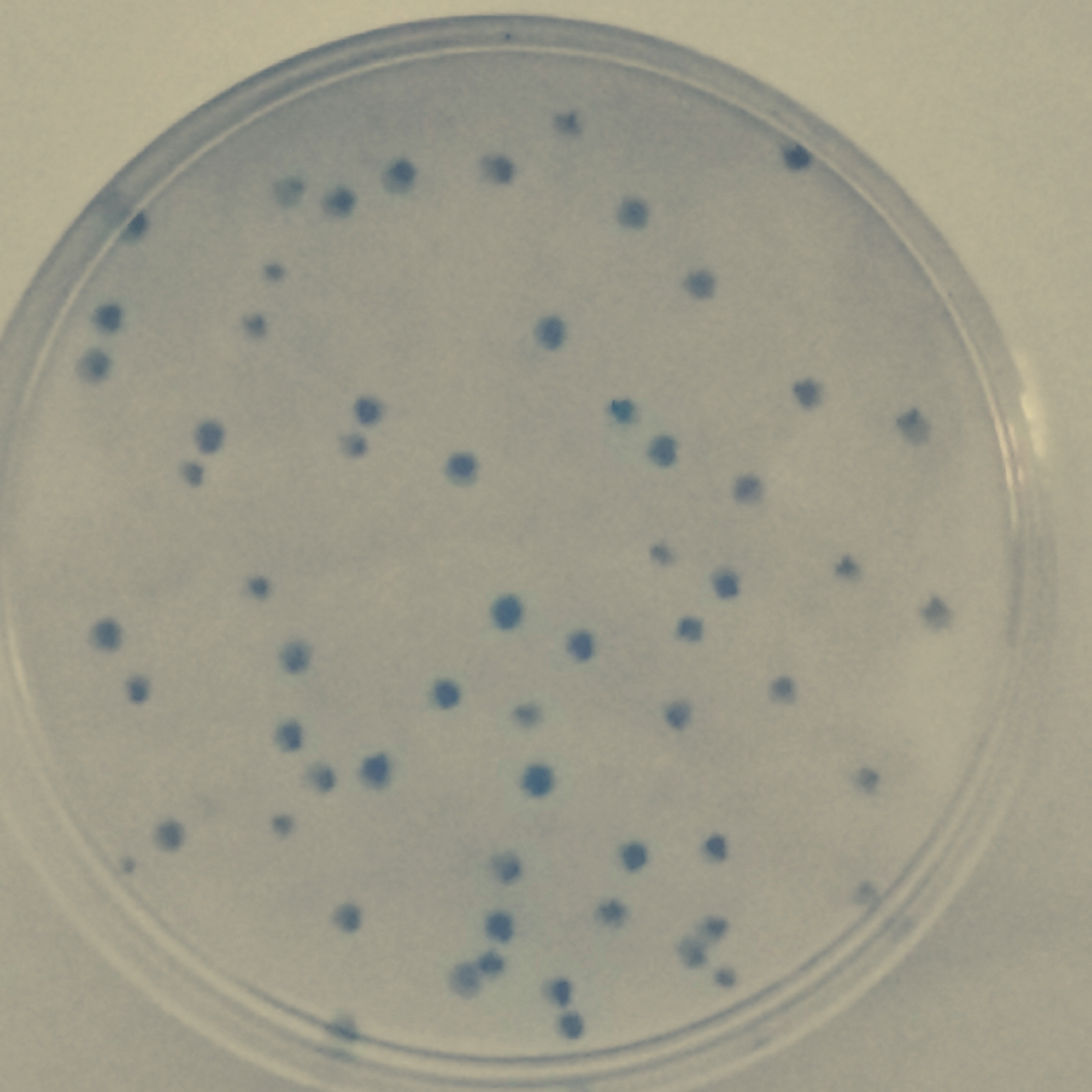

Supplement: Supplementary file 2 [file DataSheet_2.zip › fig3/colony-HCT116-3.jpg]

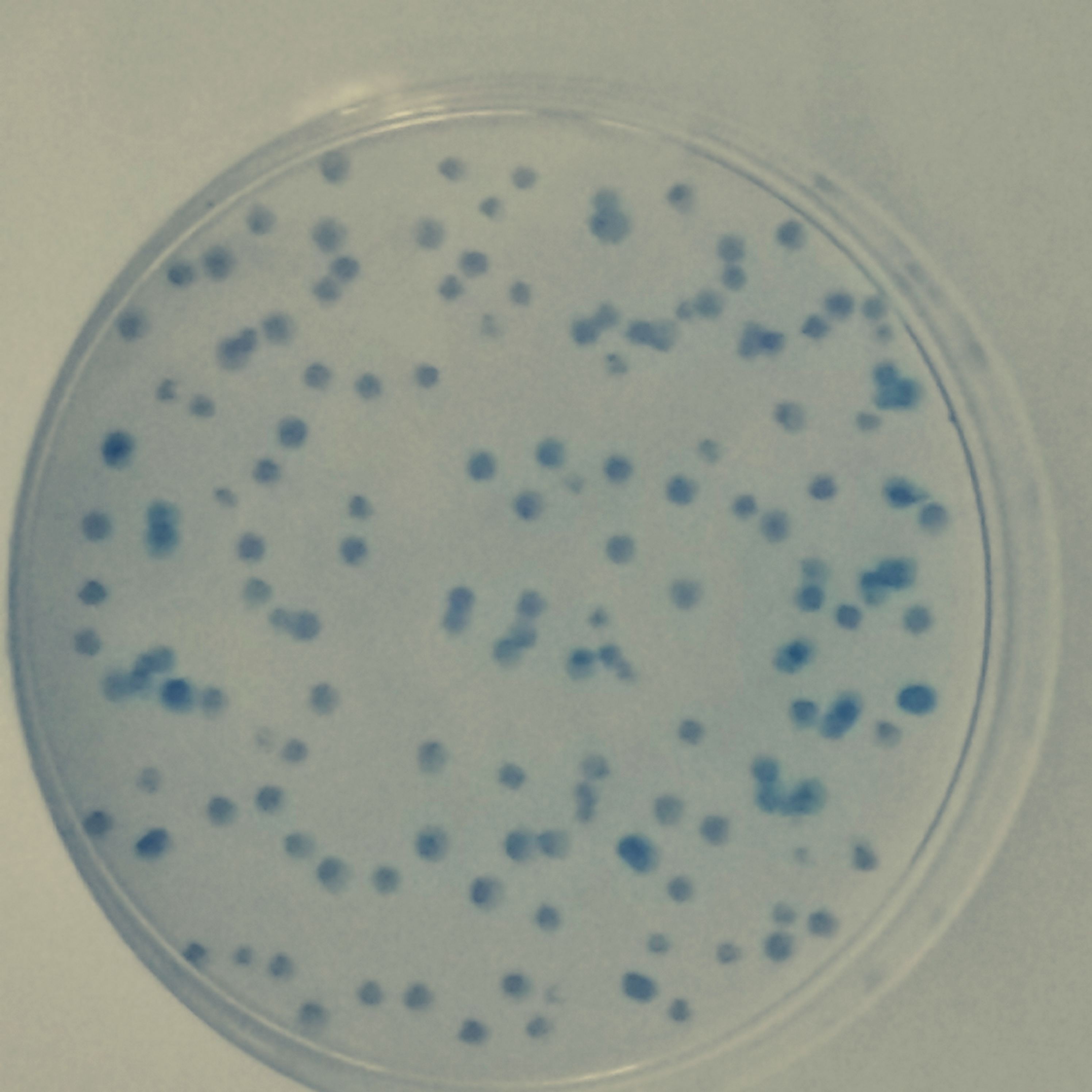

Supplement: Supplementary file 2 [file DataSheet_2.zip › fig3/colony-sw480-1.jpg]

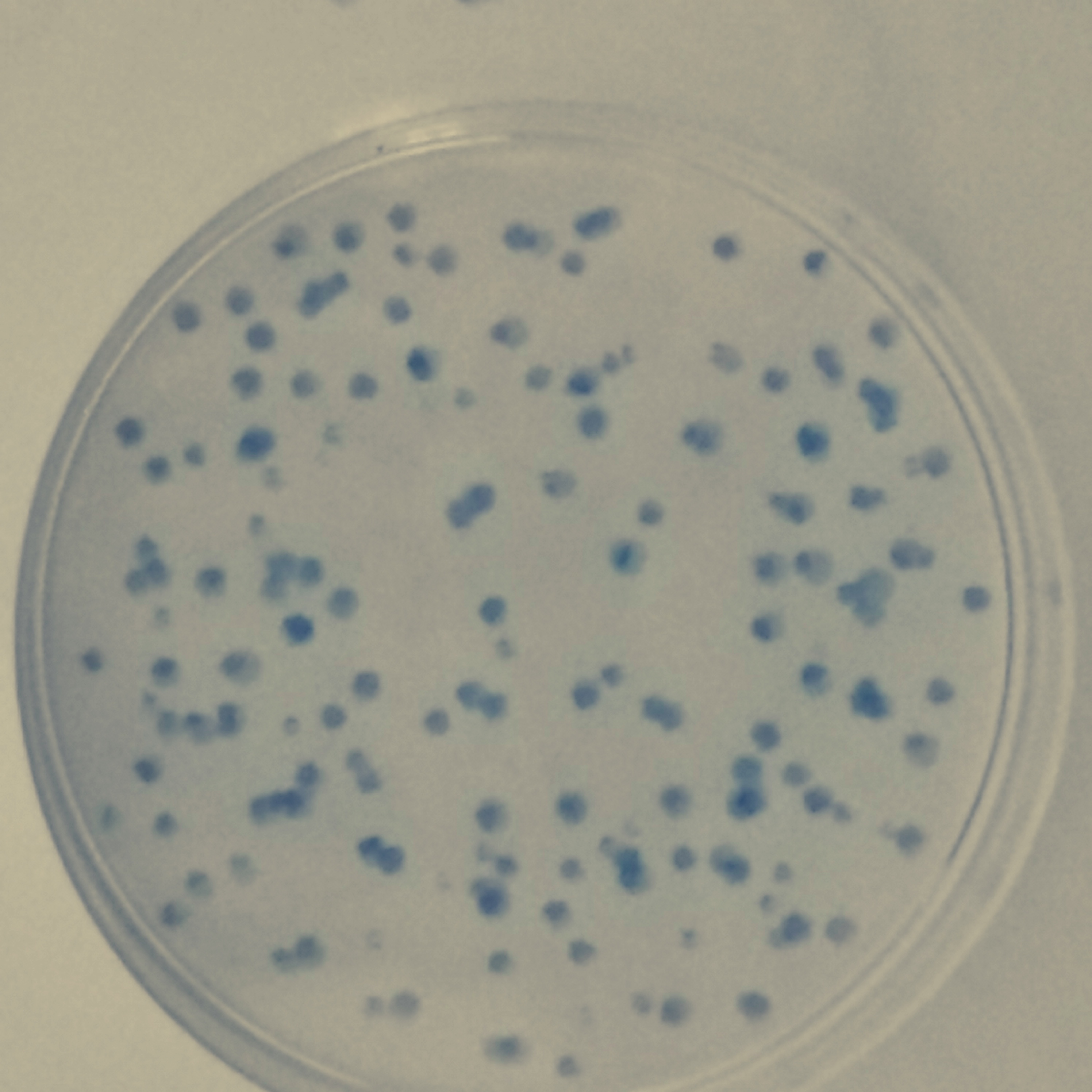

Supplement: Supplementary file 2 [file DataSheet_2.zip › fig3/colony-sw480-2.jpg]

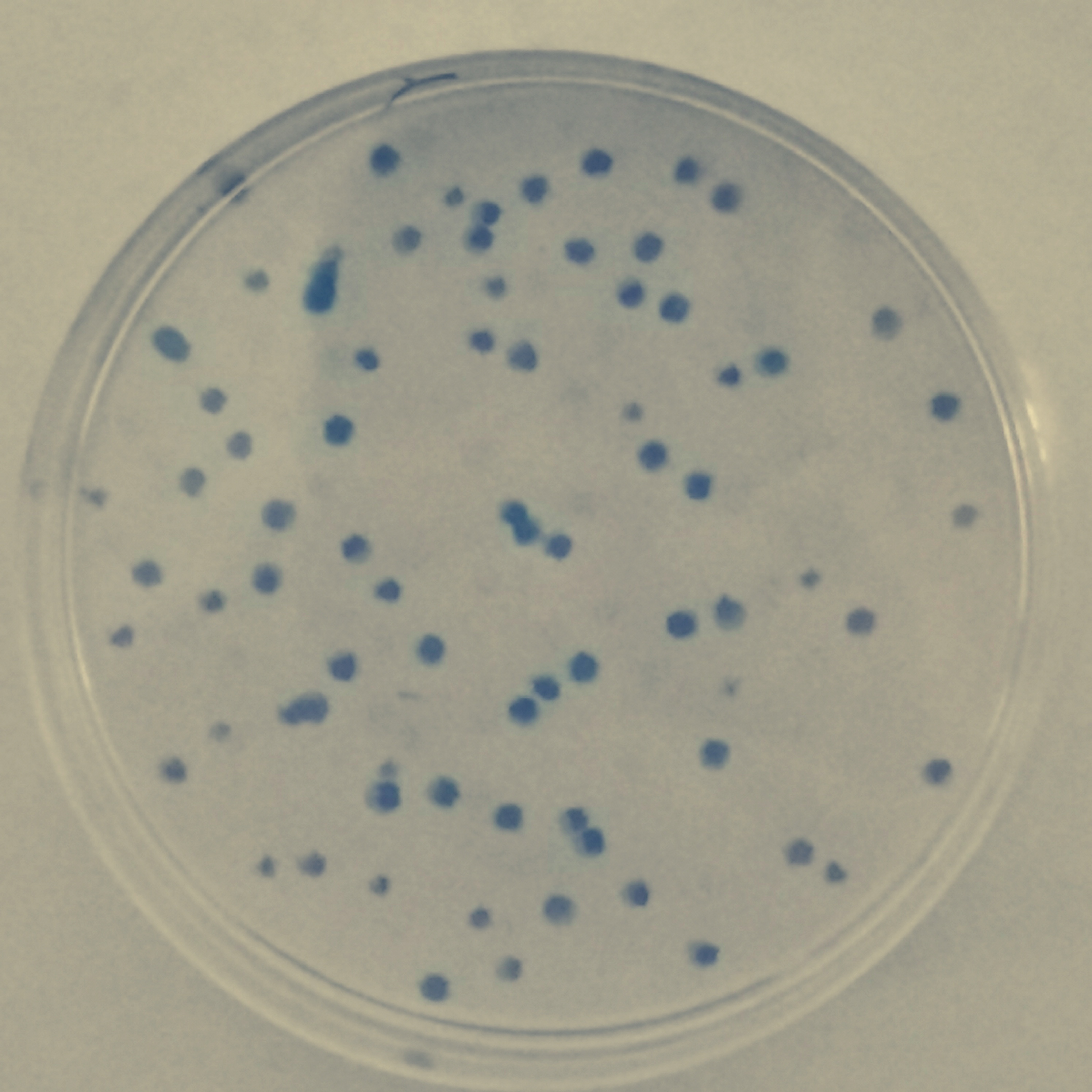

Supplement: Supplementary file 2 [file DataSheet_2.zip › fig3/colony-sw480-3.jpg]

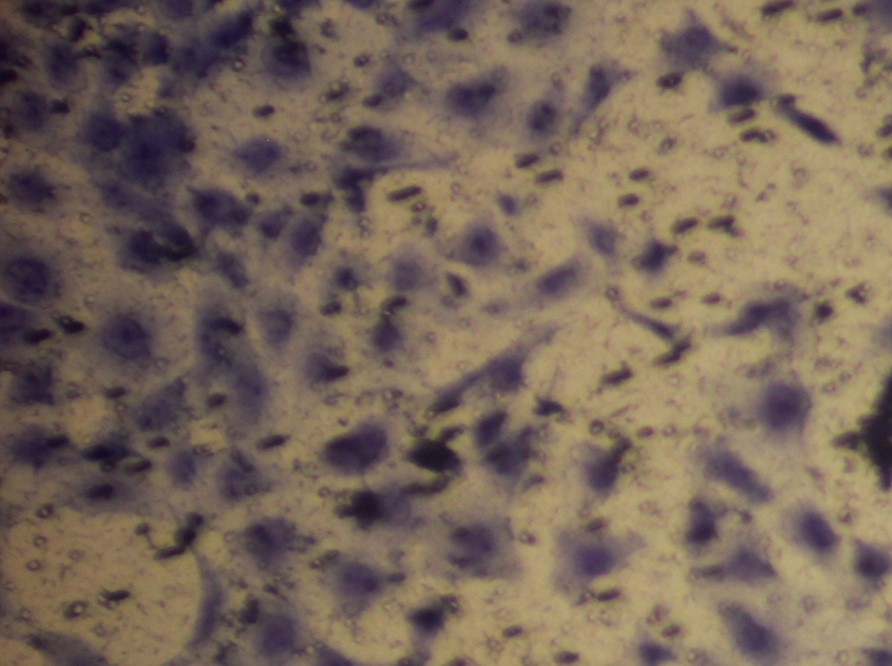

Supplement: Supplementary file 2 [file DataSheet_2.zip › fig3/HCT116-invasion-1.jpg]

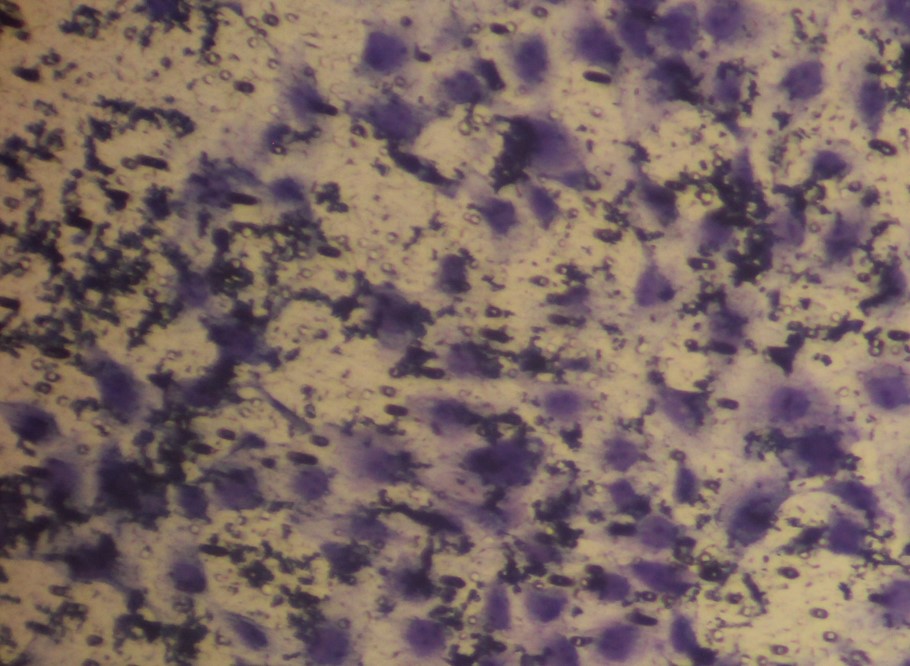

Supplement: Supplementary file 2 [file DataSheet_2.zip › fig3/HCT116-invasion-2.jpg]

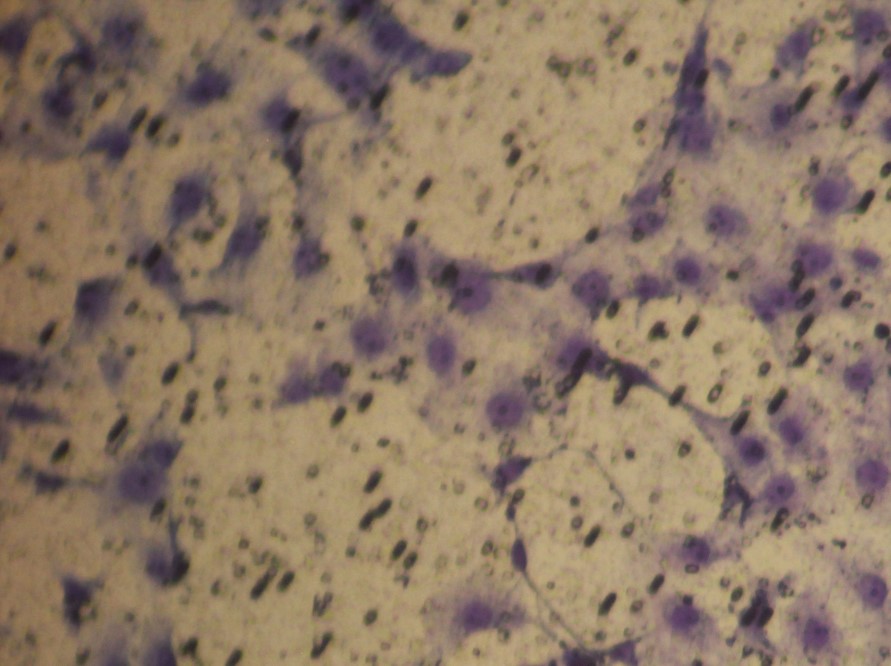

Supplement: Supplementary file 2 [file DataSheet_2.zip › fig3/HCT116-invasion-3.jpg]

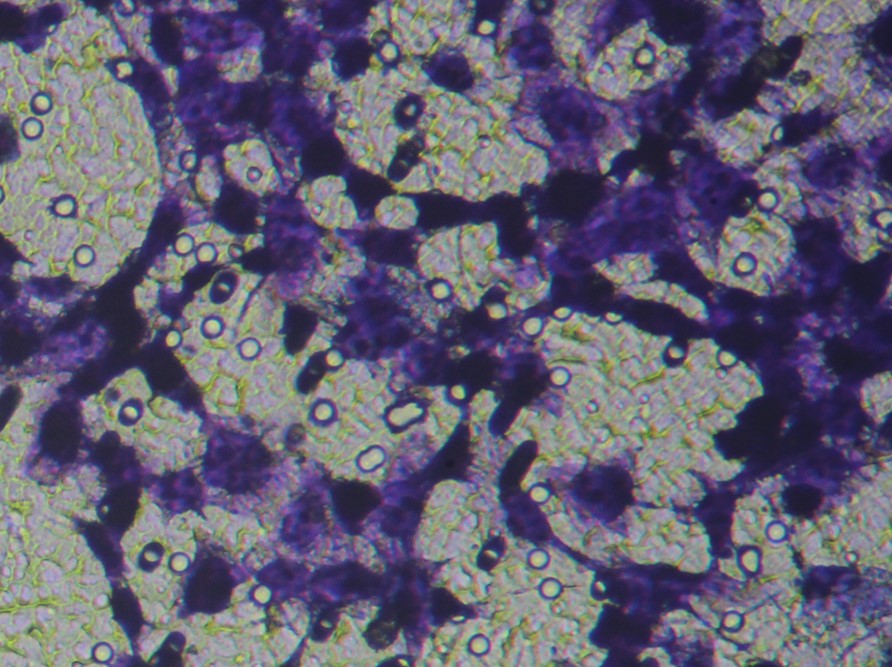

Supplement: Supplementary file 2 [file DataSheet_2.zip › fig3/sw480-invasion-1.jpg]

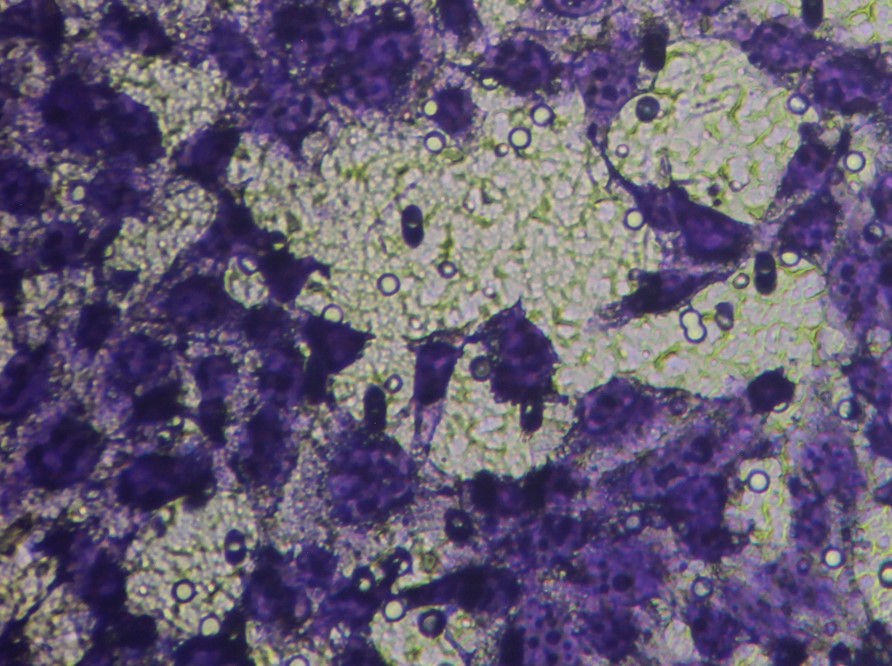

Supplement: Supplementary file 2 [file DataSheet_2.zip › fig3/sw480-invasion-2.jpg]

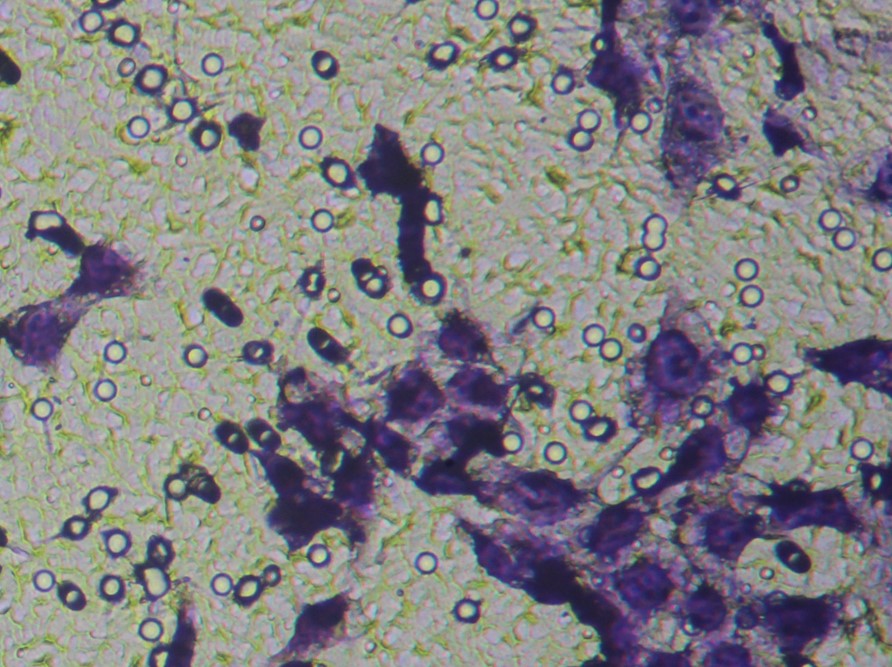

Supplement: Supplementary file 2 [file DataSheet_2.zip › fig3/sw480-invasion-3.jpg]

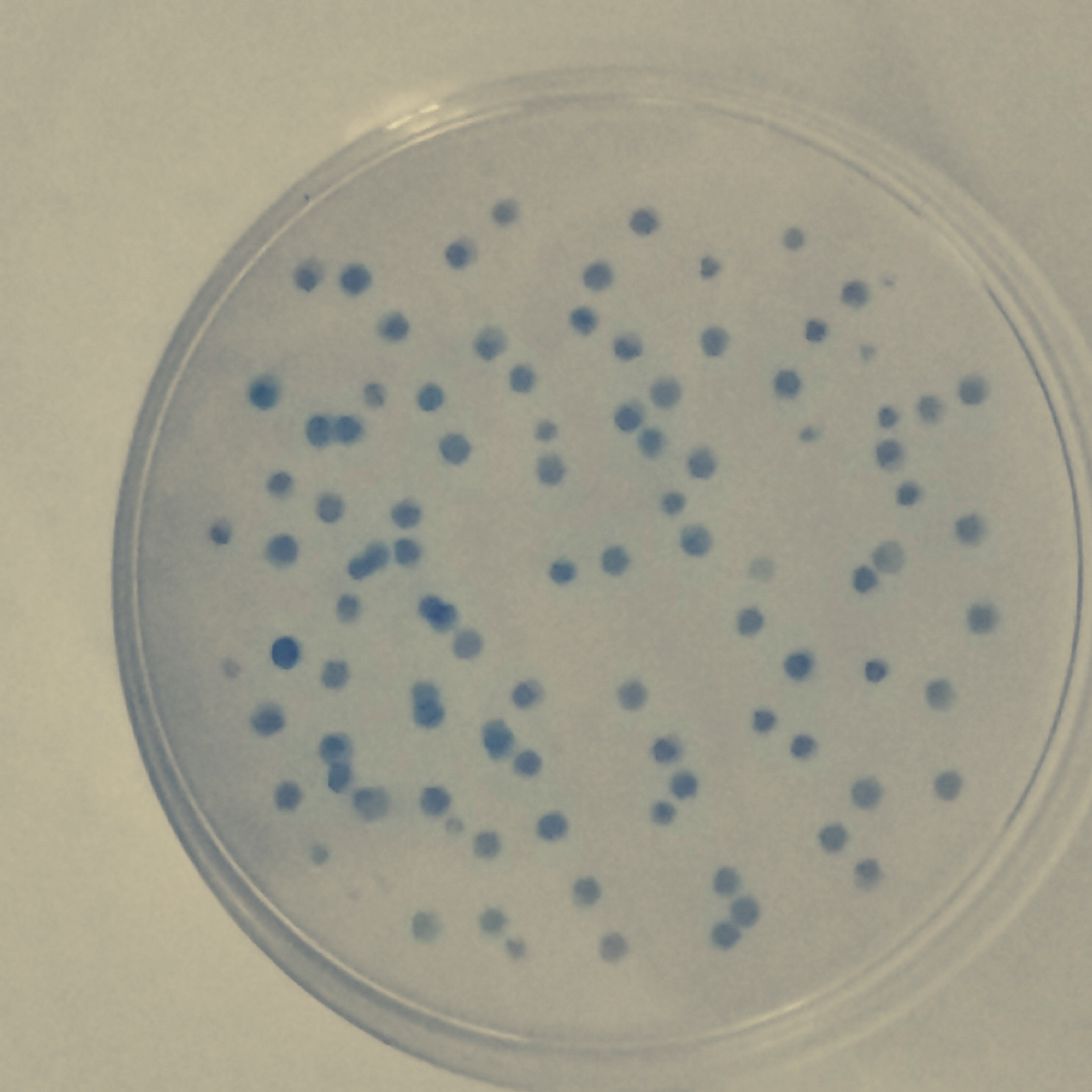

Supplement: Supplementary file 3 [file DataSheet_3.zip › fig6/colony-1.jpg]

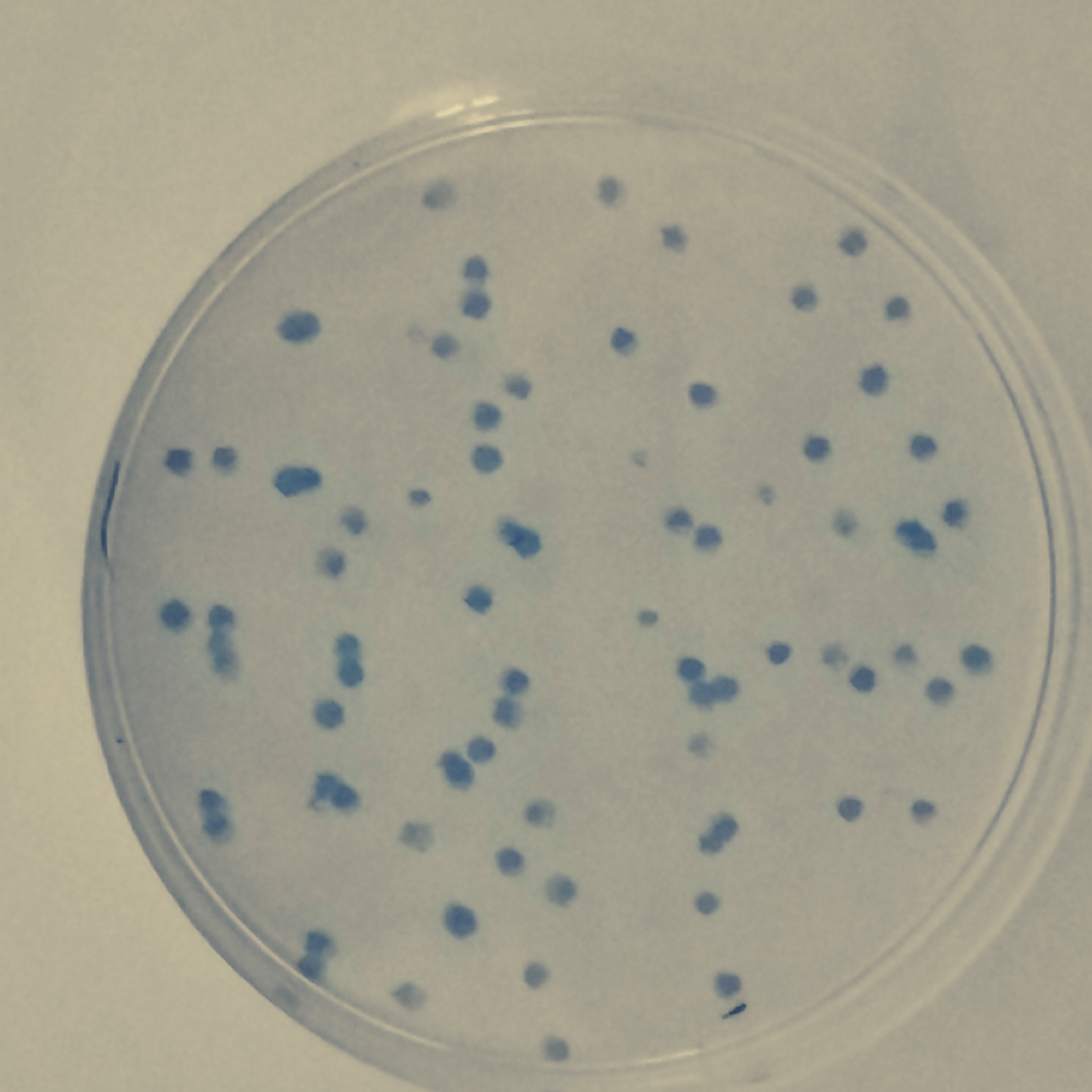

Supplement: Supplementary file 3 [file DataSheet_3.zip › fig6/colony-2.jpg]

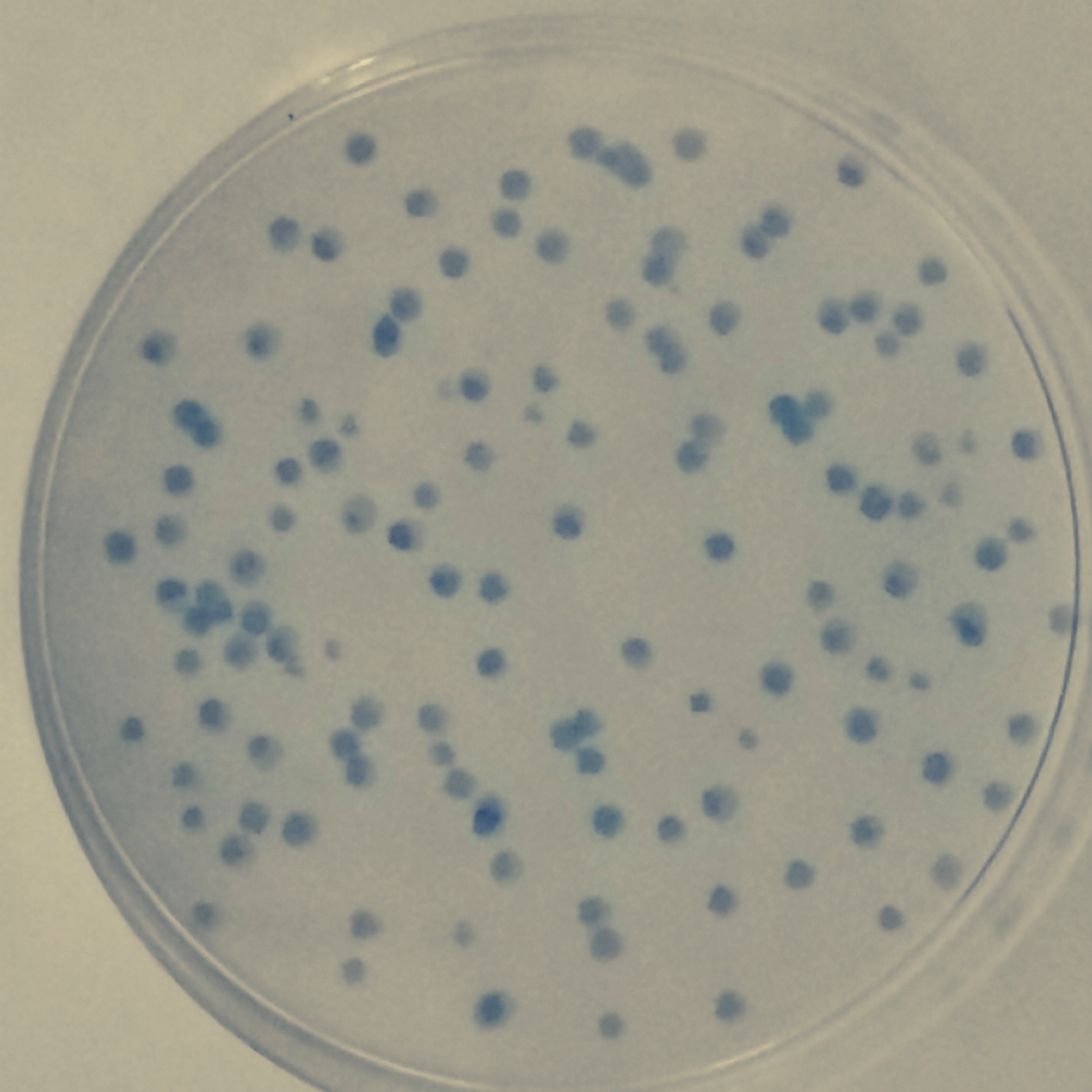

Supplement: Supplementary file 3 [file DataSheet_3.zip › fig6/colony-3.jpg]

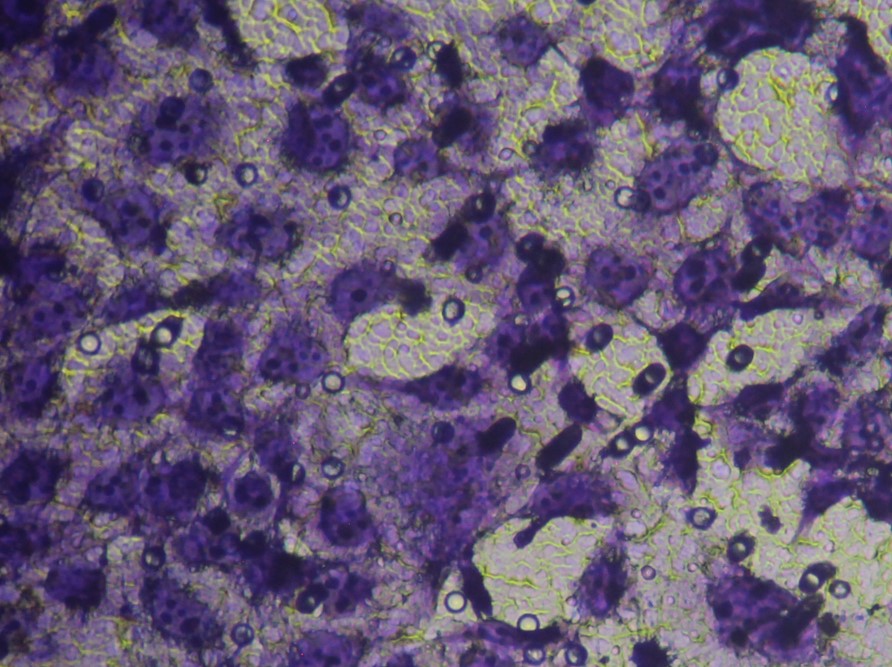

Supplement: Supplementary file 3 [file DataSheet_3.zip › fig6/invasion-1.jpg]

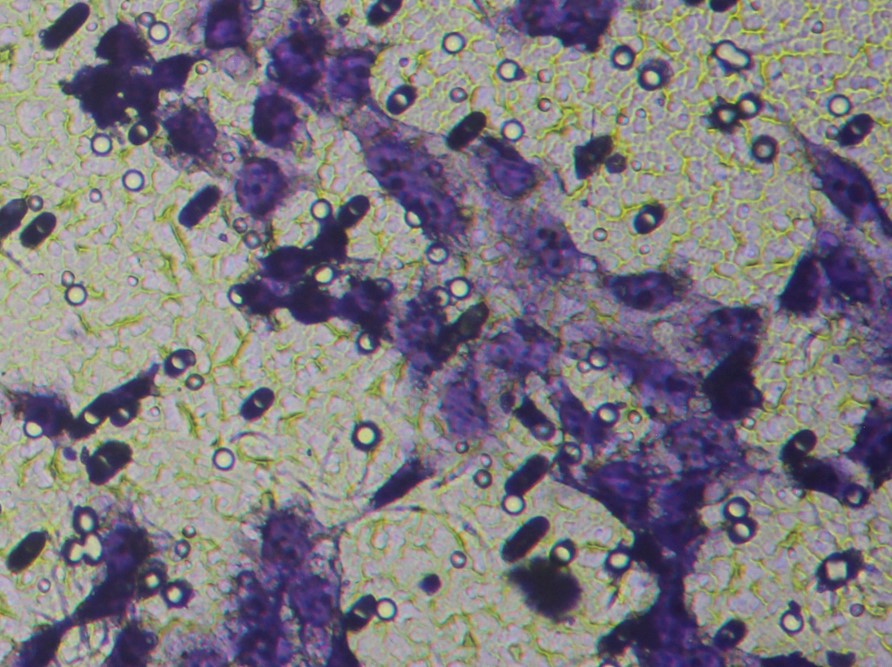

Supplement: Supplementary file 3 [file DataSheet_3.zip › fig6/invasion-2.jpg]

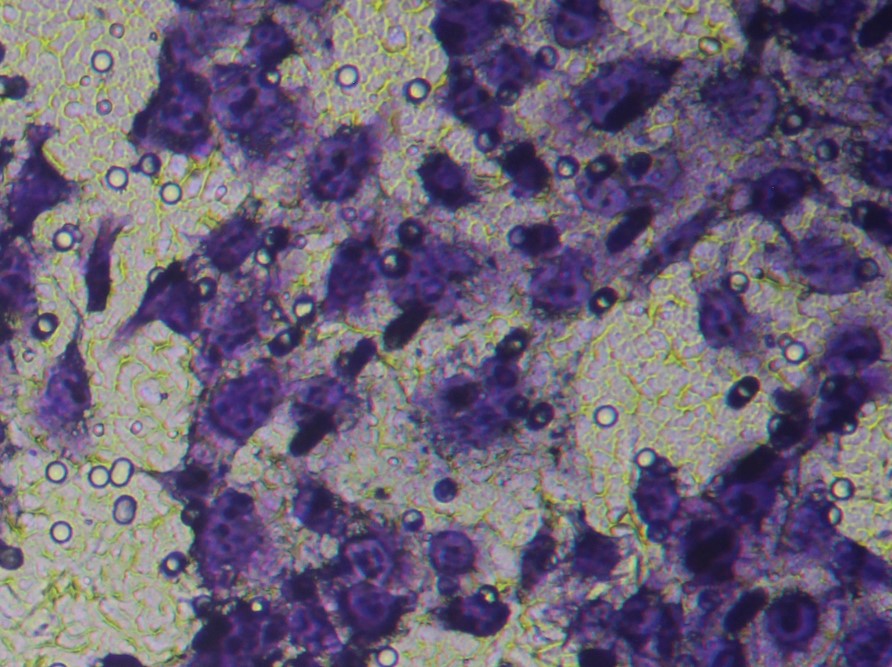

Supplement: Supplementary file 3 [file DataSheet_3.zip › fig6/invasion-3.jpg]

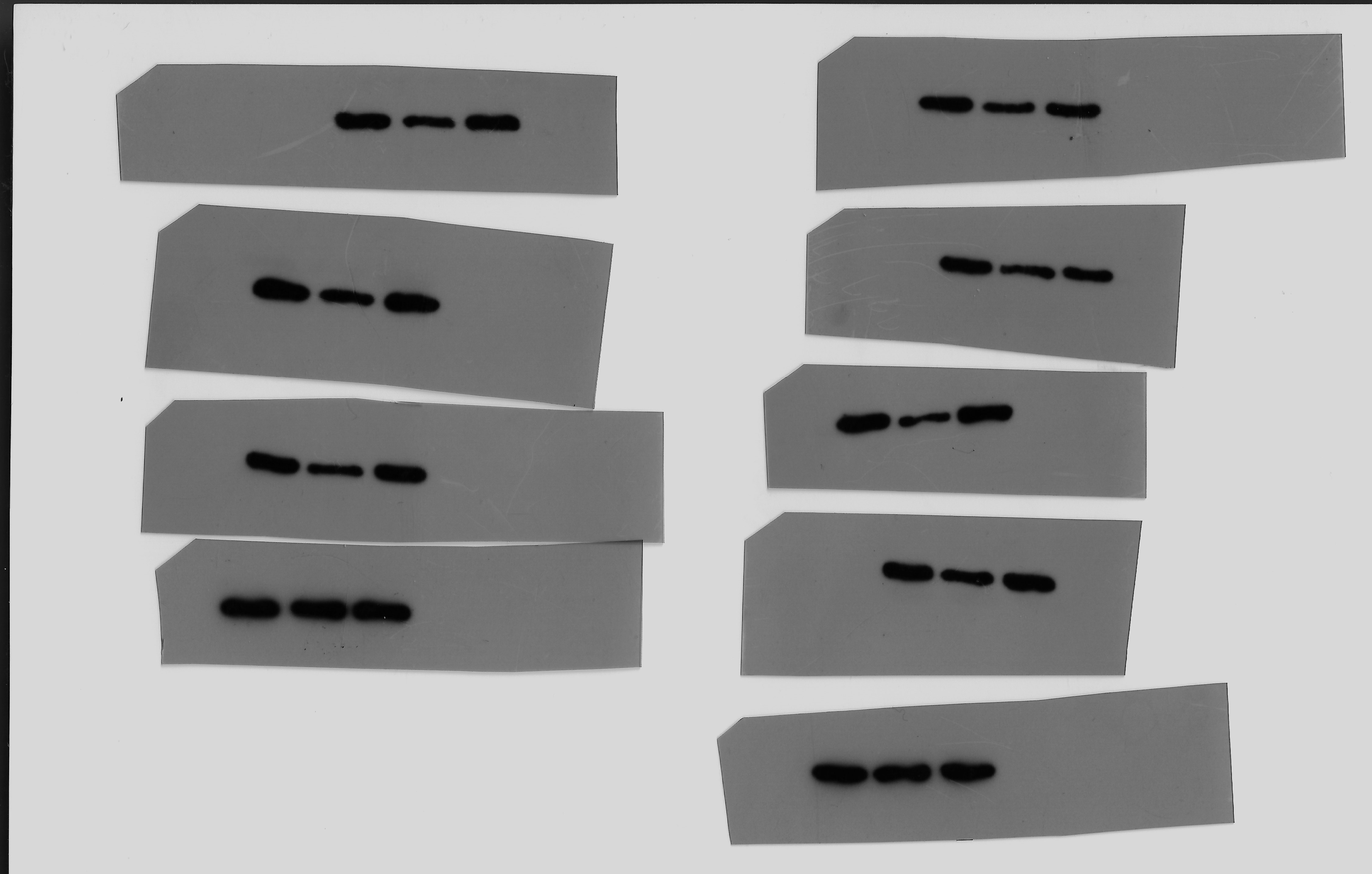

Supplement: Supplementary file 3 [file DataSheet_3.zip › fig6/western blot.jpg]

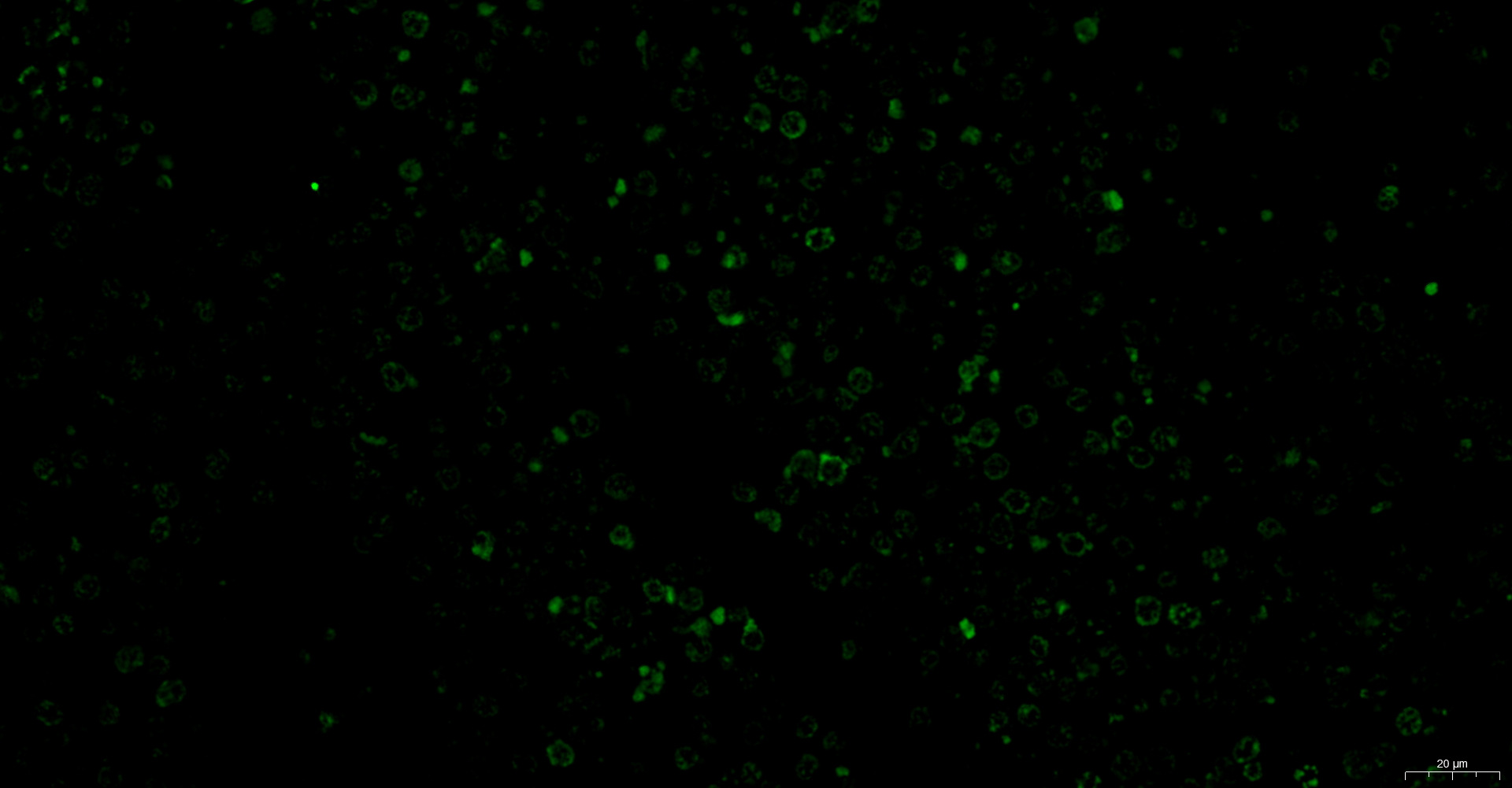

Supplement: Supplementary file 3 [file DataSheet_3.zip › fig5/D1-1-1.jpg]

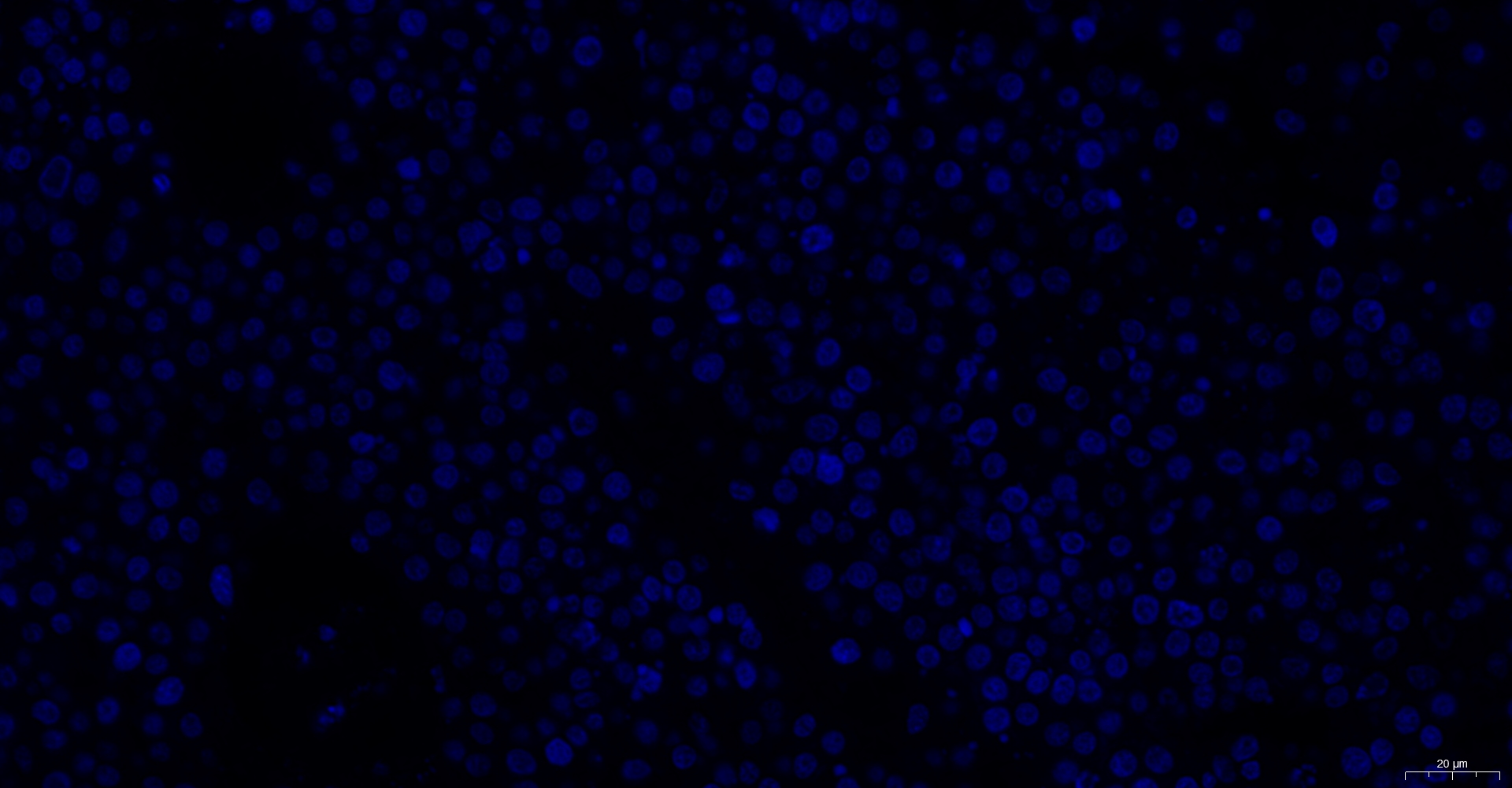

Supplement: Supplementary file 3 [file DataSheet_3.zip › fig5/D1-1-2.jpg]

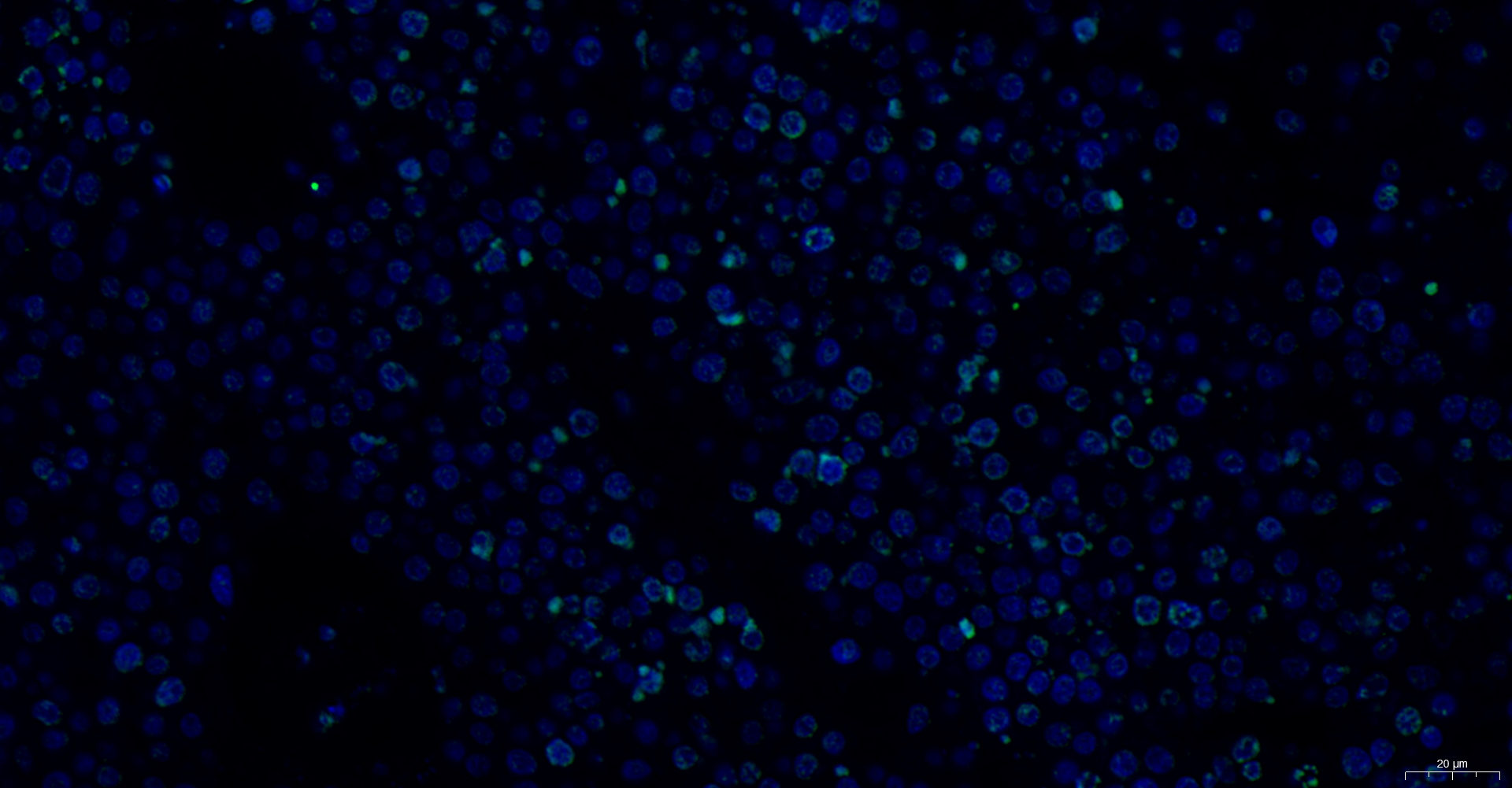

Supplement: Supplementary file 3 [file DataSheet_3.zip › fig5/D1-1-3.jpg]

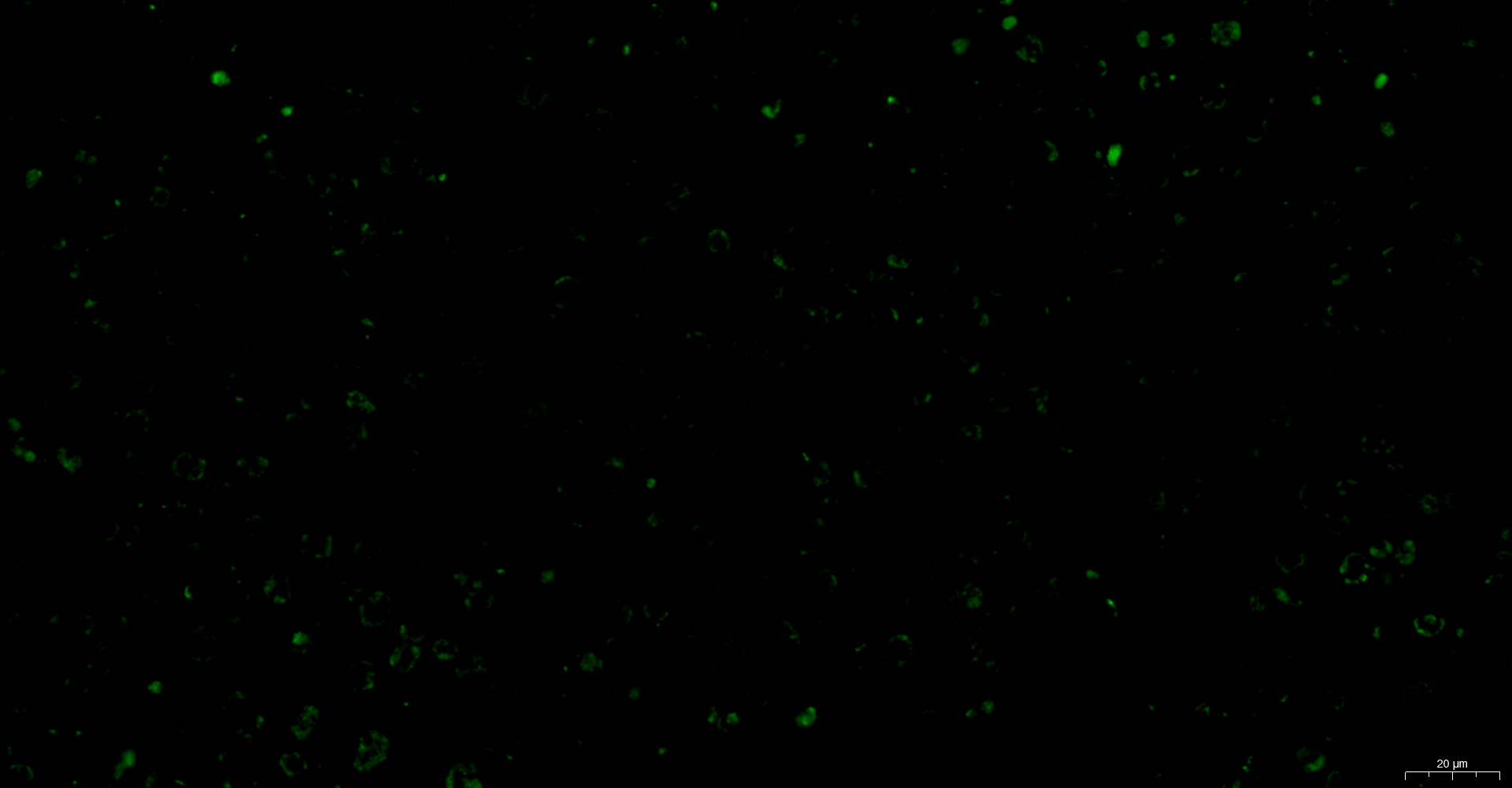

Supplement: Supplementary file 3 [file DataSheet_3.zip › fig5/D1-2-1.jpg]

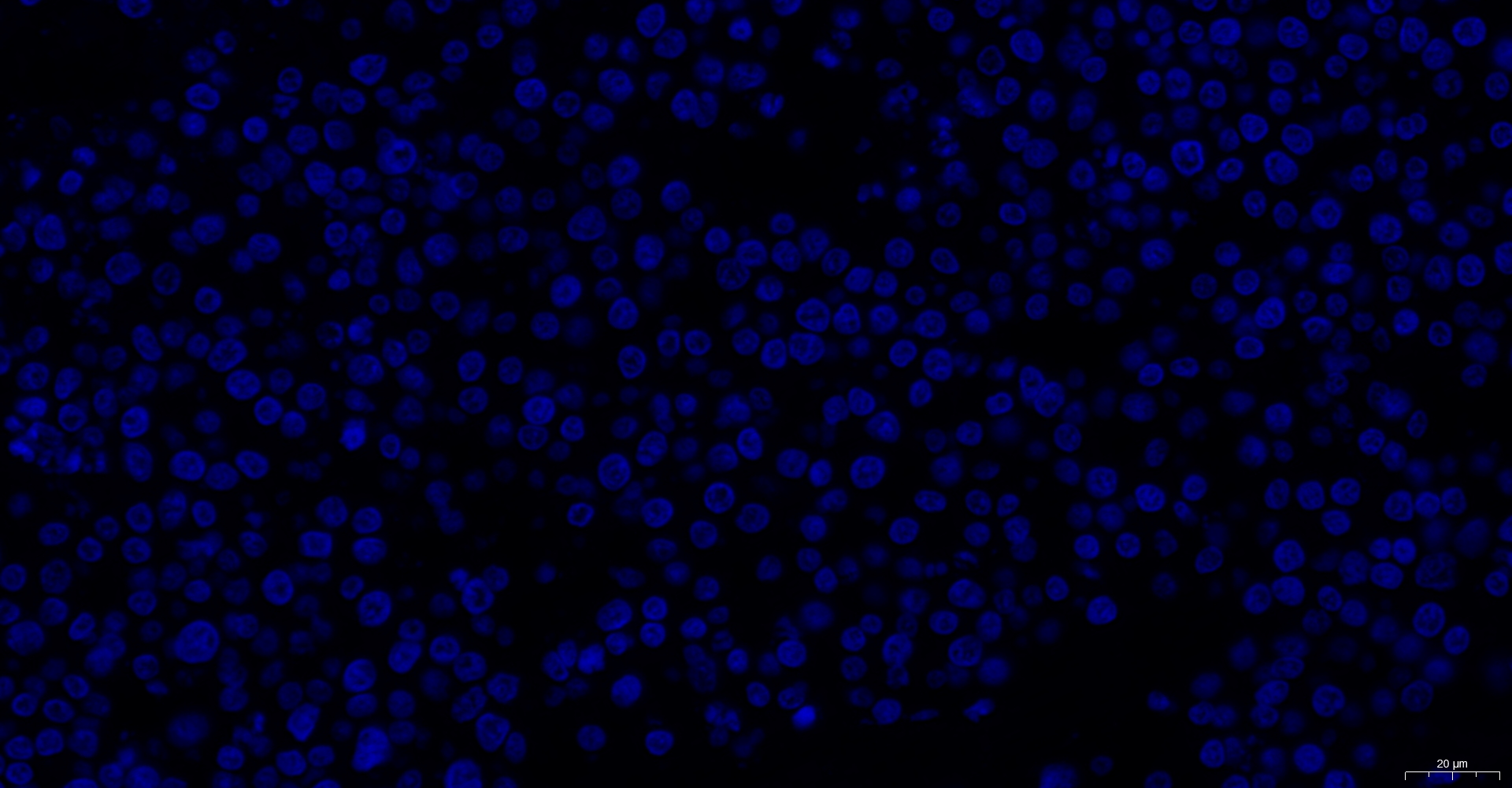

Supplement: Supplementary file 3 [file DataSheet_3.zip › fig5/D1-2-2.jpg]

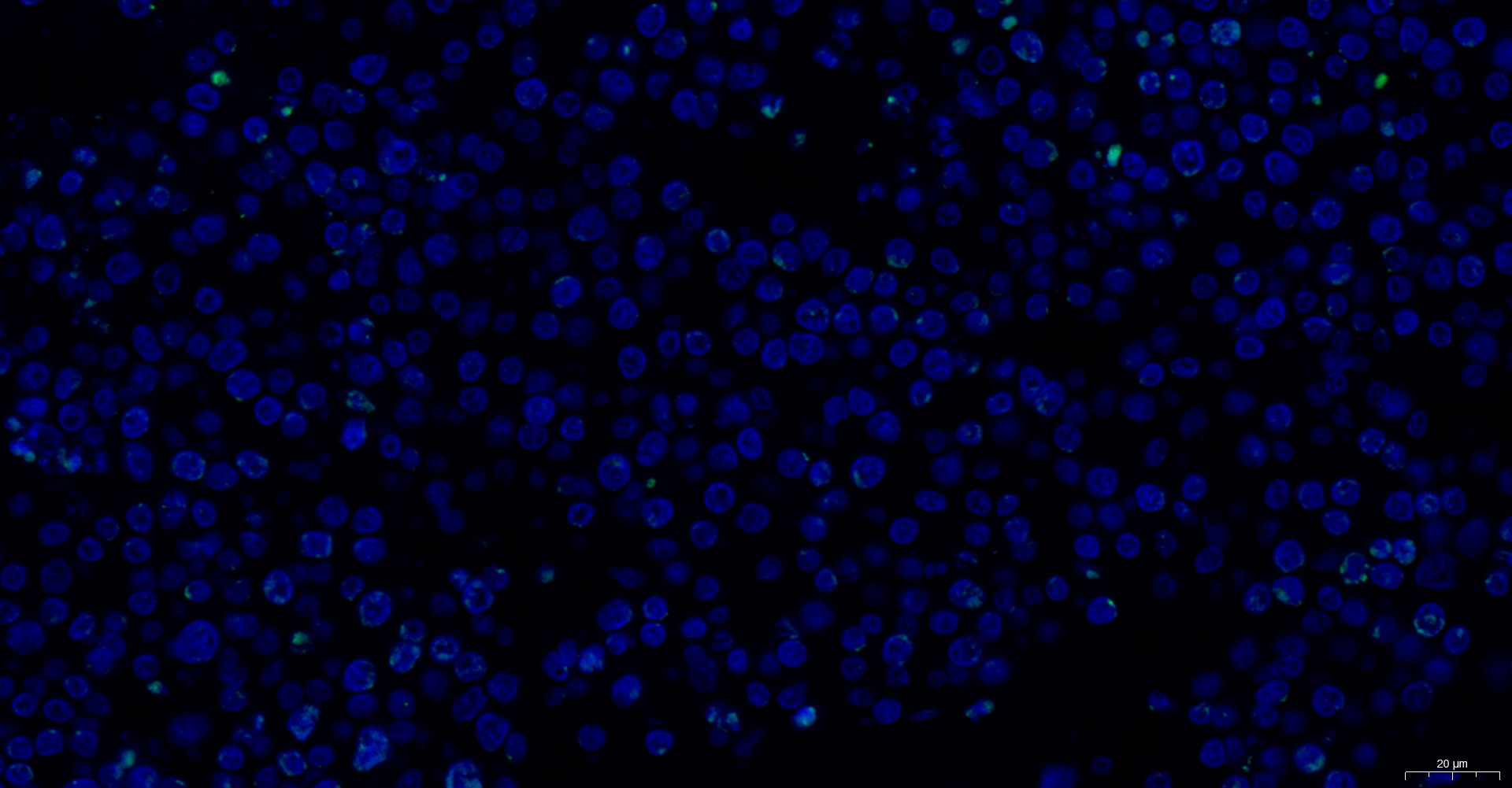

Supplement: Supplementary file 3 [file DataSheet_3.zip › fig5/D1-2-3.jpg]

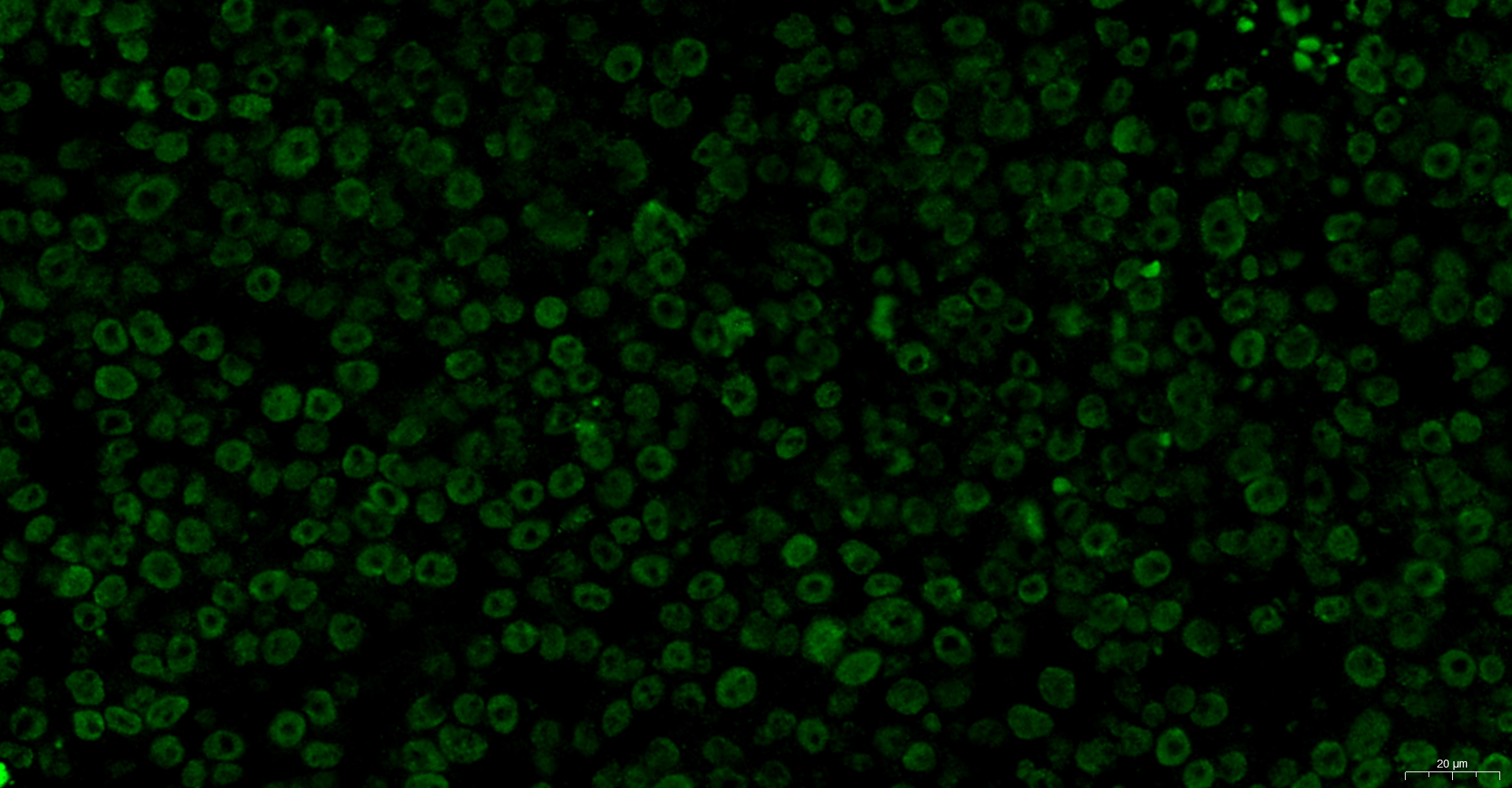

Supplement: Supplementary file 3 [file DataSheet_3.zip › fig5/D1-3-1.jpg]

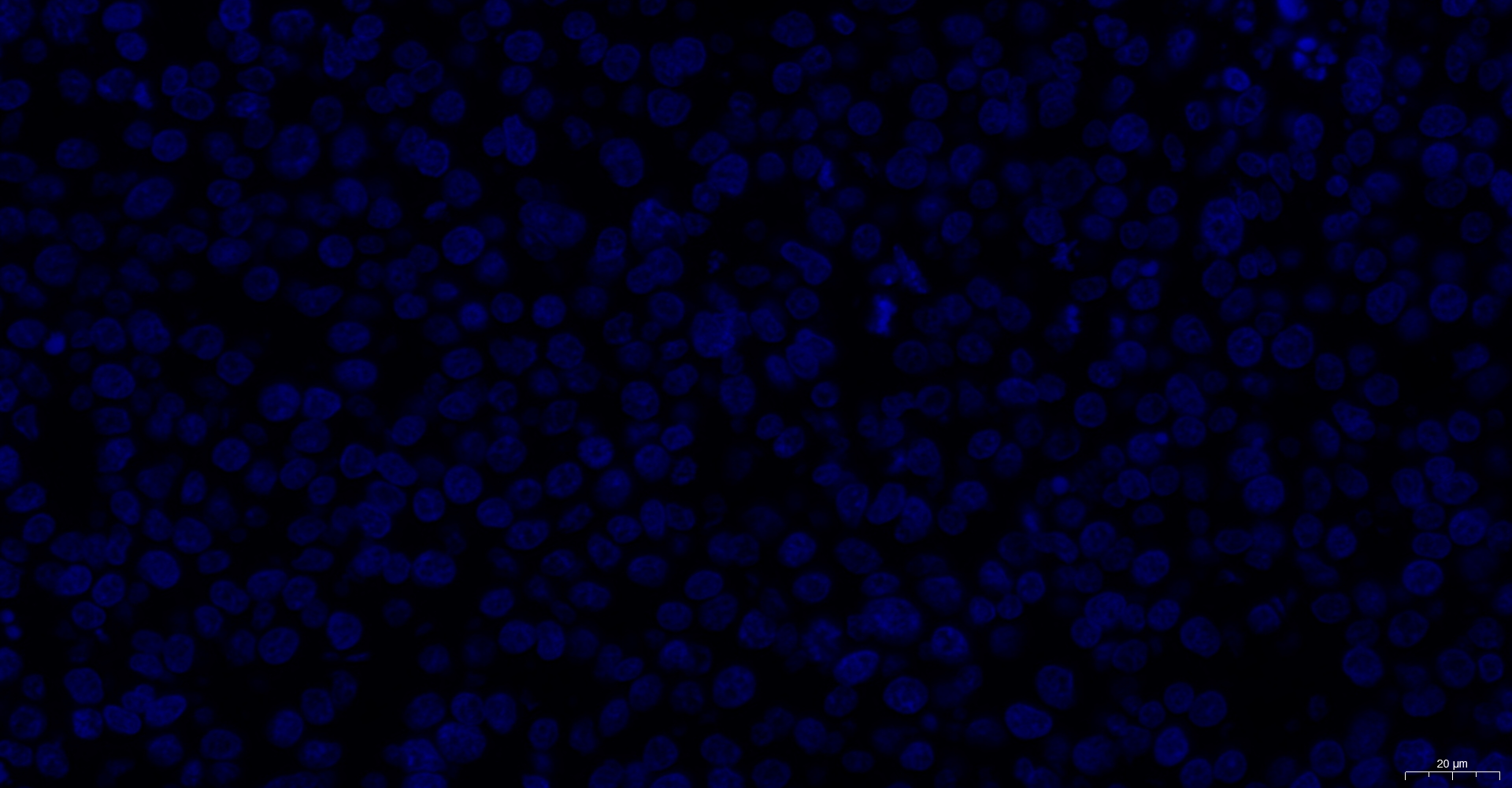

Supplement: Supplementary file 3 [file DataSheet_3.zip › fig5/D1-3-2.jpg]

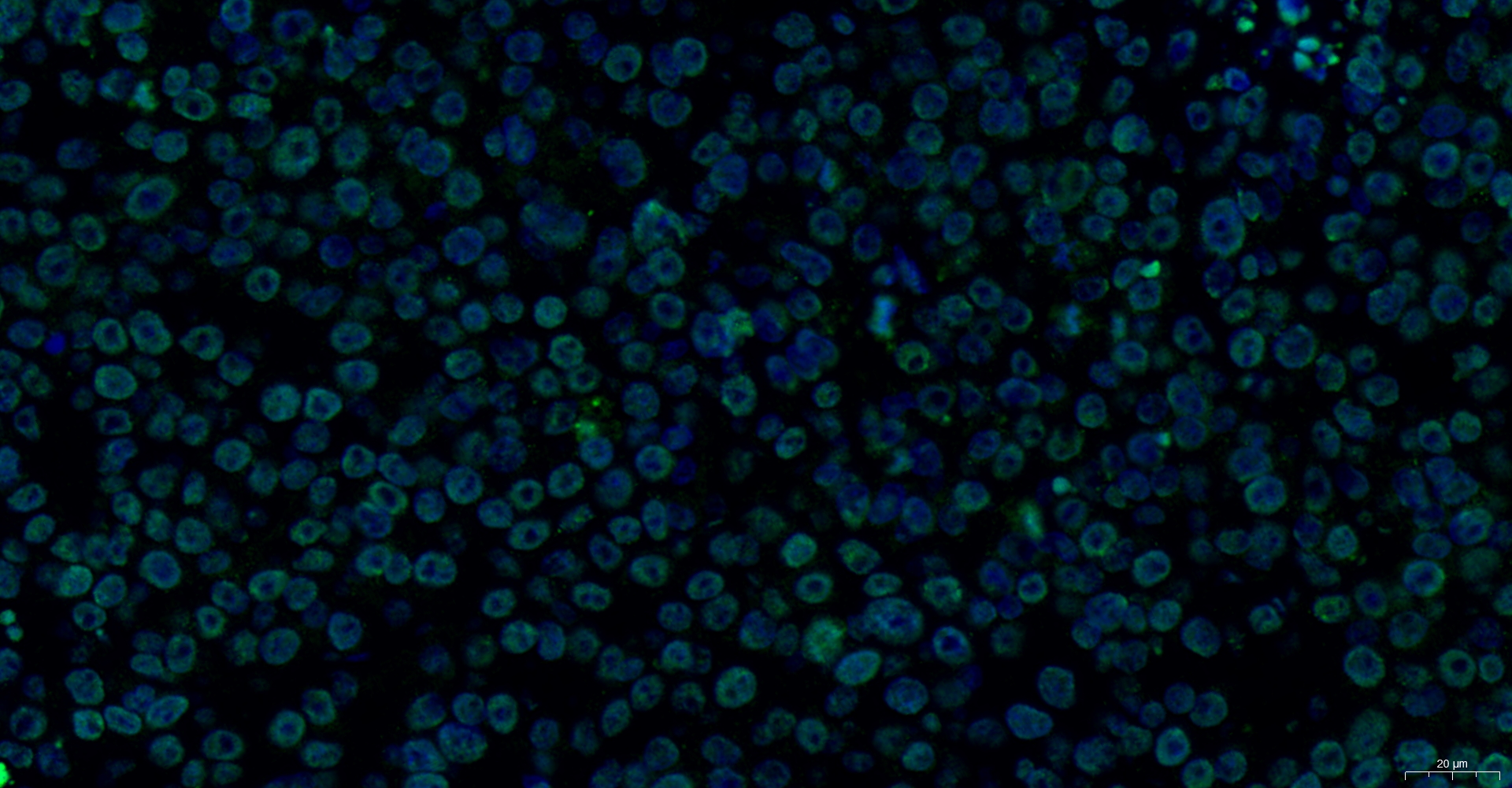

Supplement: Supplementary file 3 [file DataSheet_3.zip › fig5/D1-3-3.jpg]

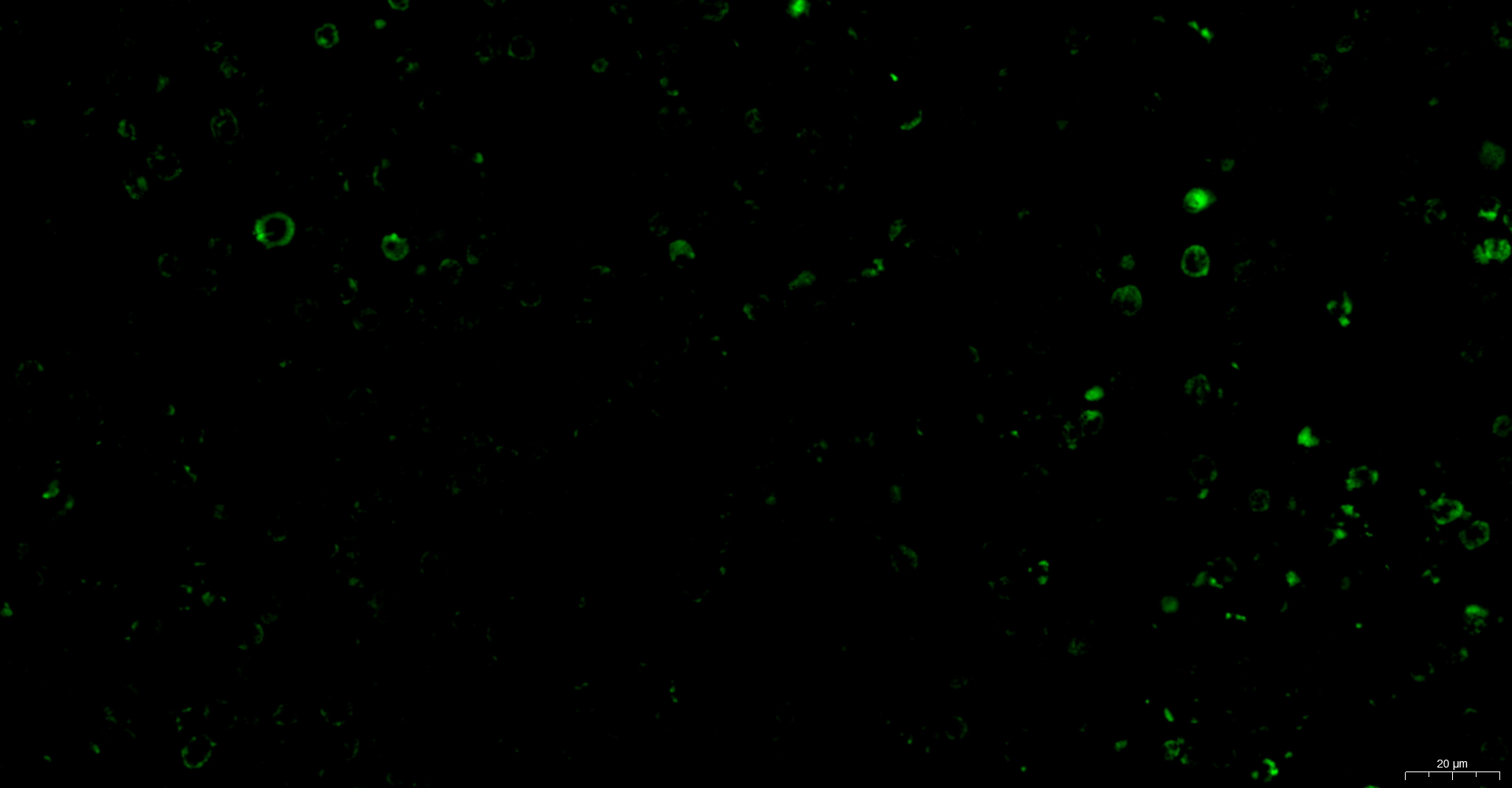

Supplement: Supplementary file 3 [file DataSheet_3.zip › fig5/E2-1-1.jpg]

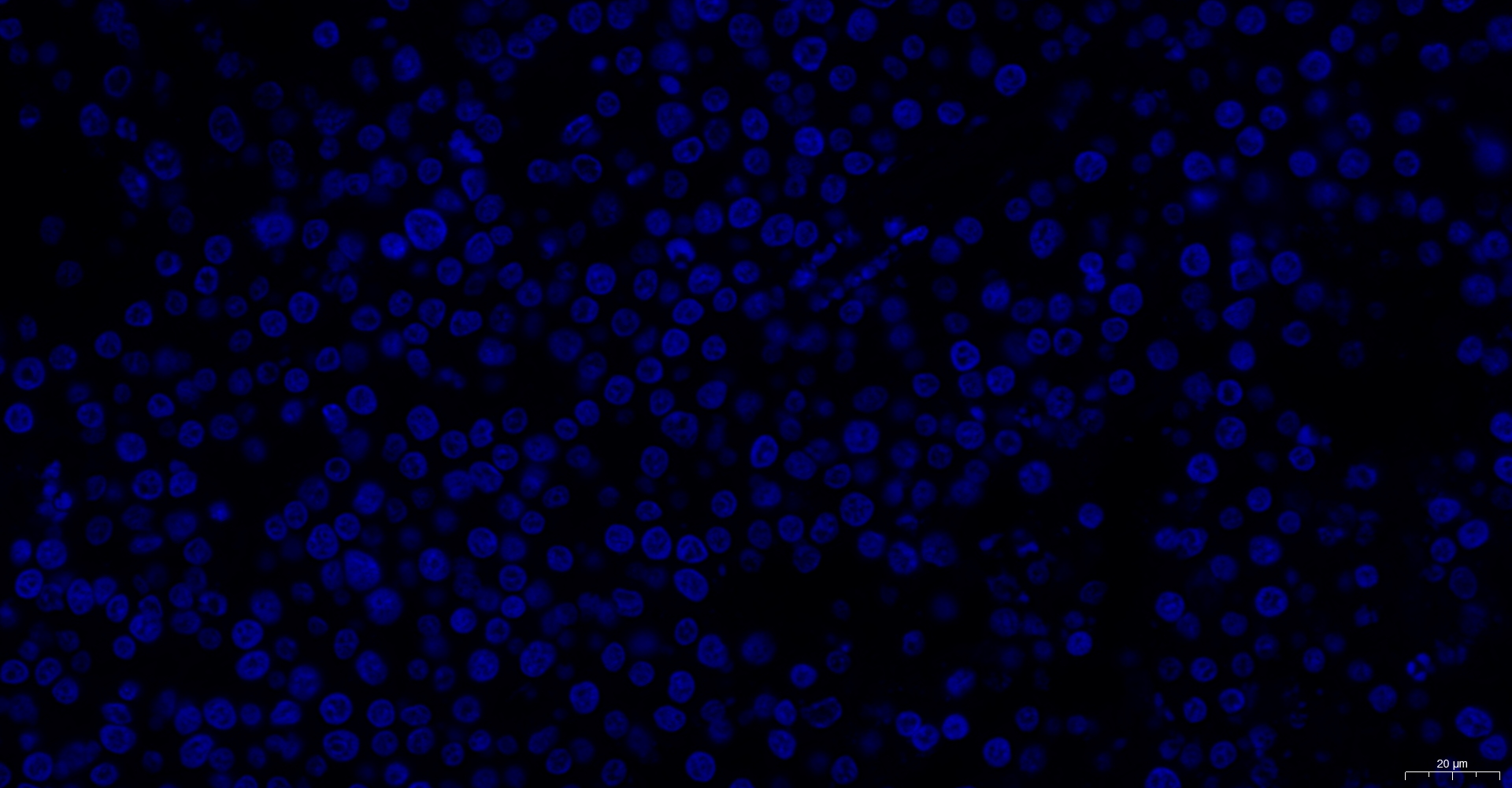

Supplement: Supplementary file 3 [file DataSheet_3.zip › fig5/E2-1-2.jpg]

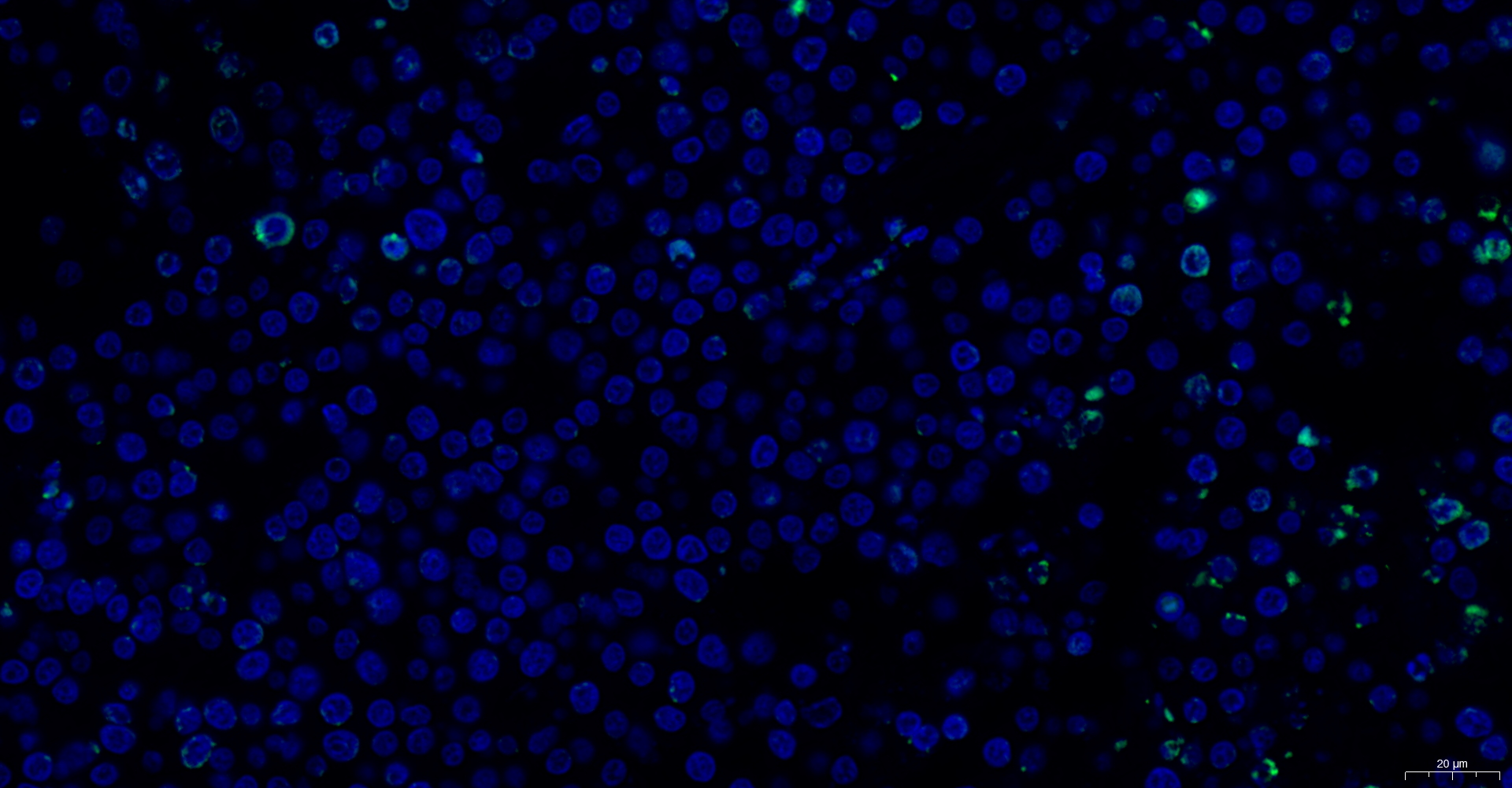

Supplement: Supplementary file 3 [file DataSheet_3.zip › fig5/E2-1-3.jpg]

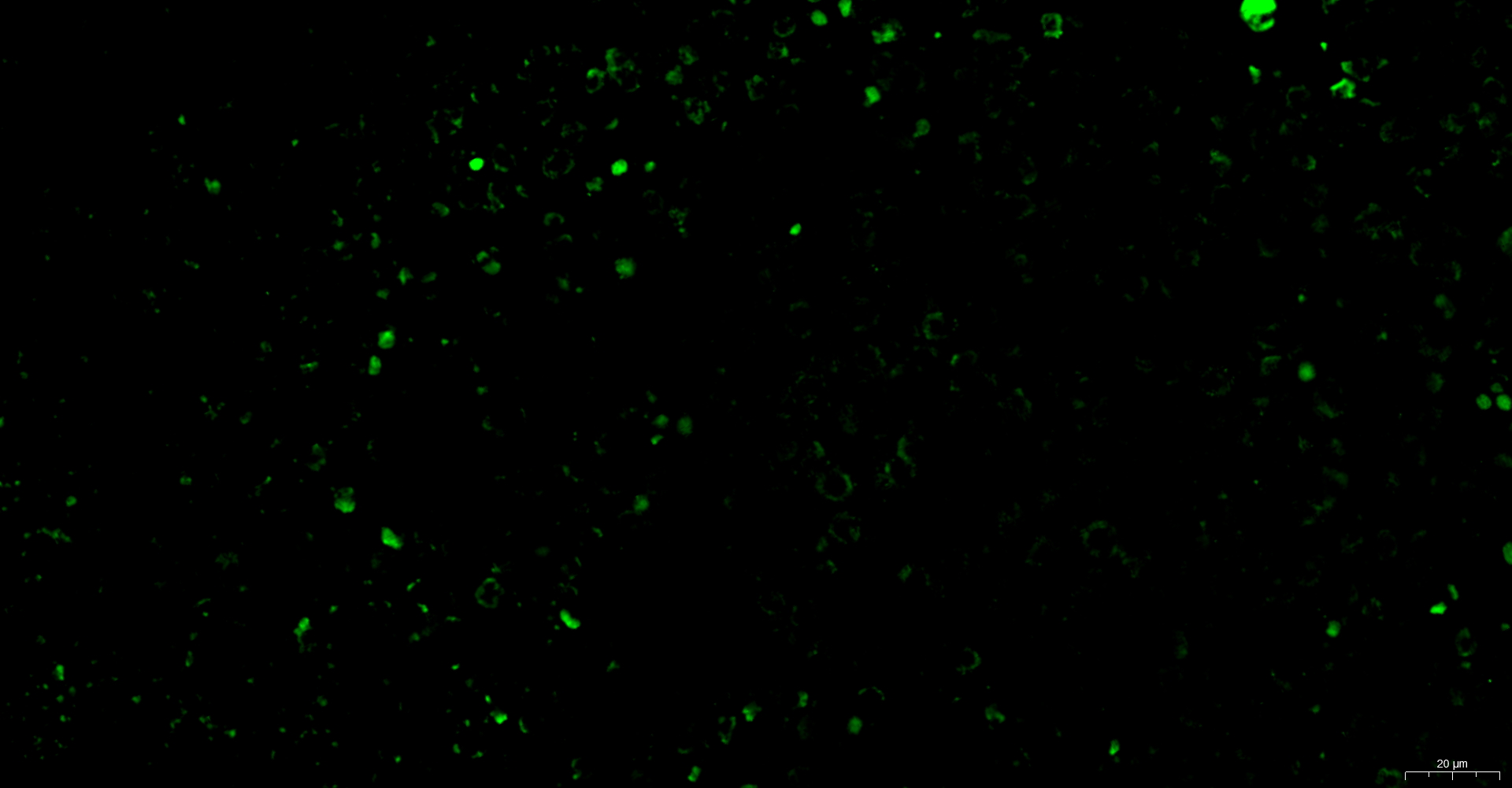

Supplement: Supplementary file 3 [file DataSheet_3.zip › fig5/E2-2-1.jpg]

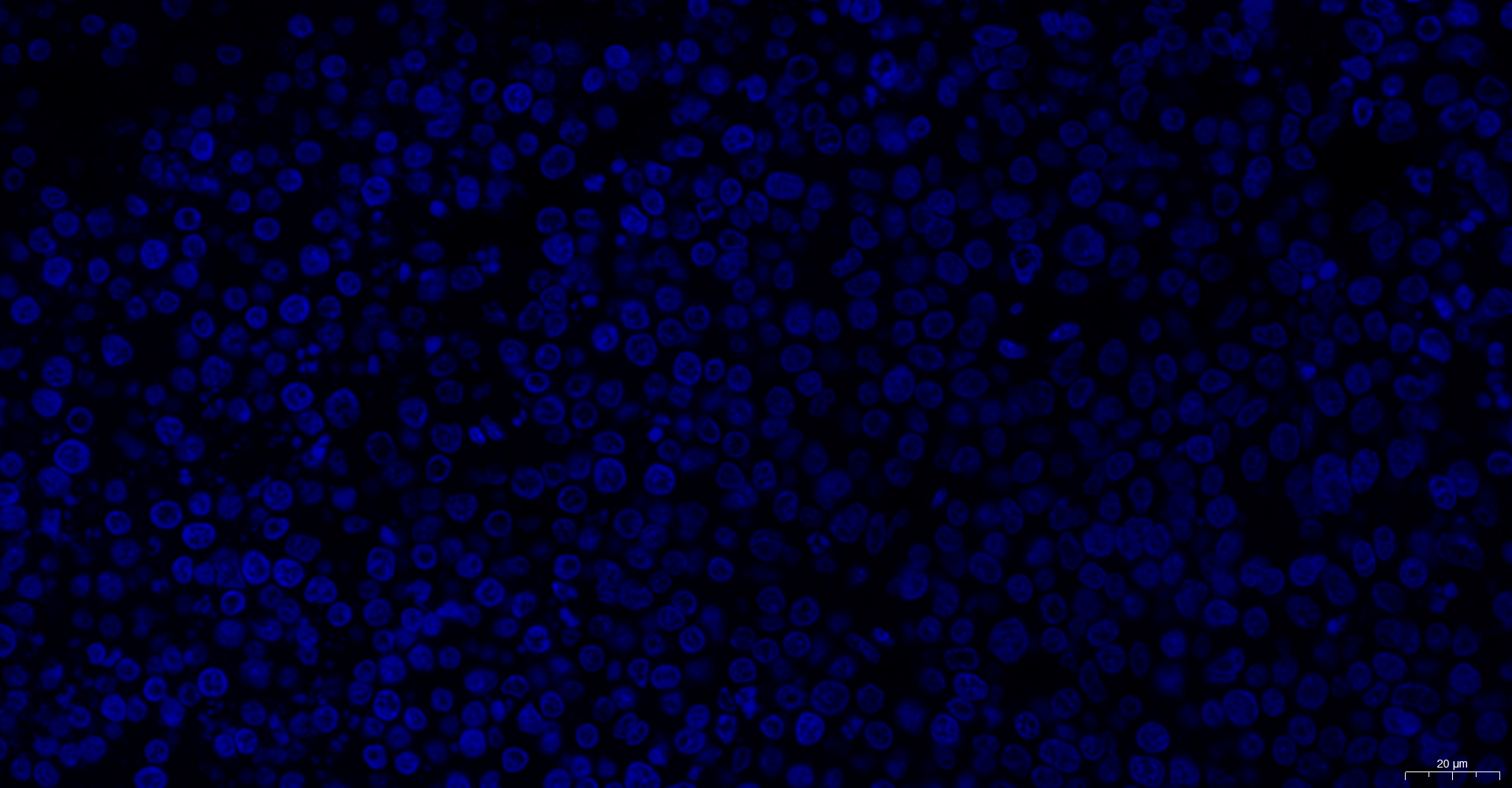

Supplement: Supplementary file 3 [file DataSheet_3.zip › fig5/E2-2-2.jpg]

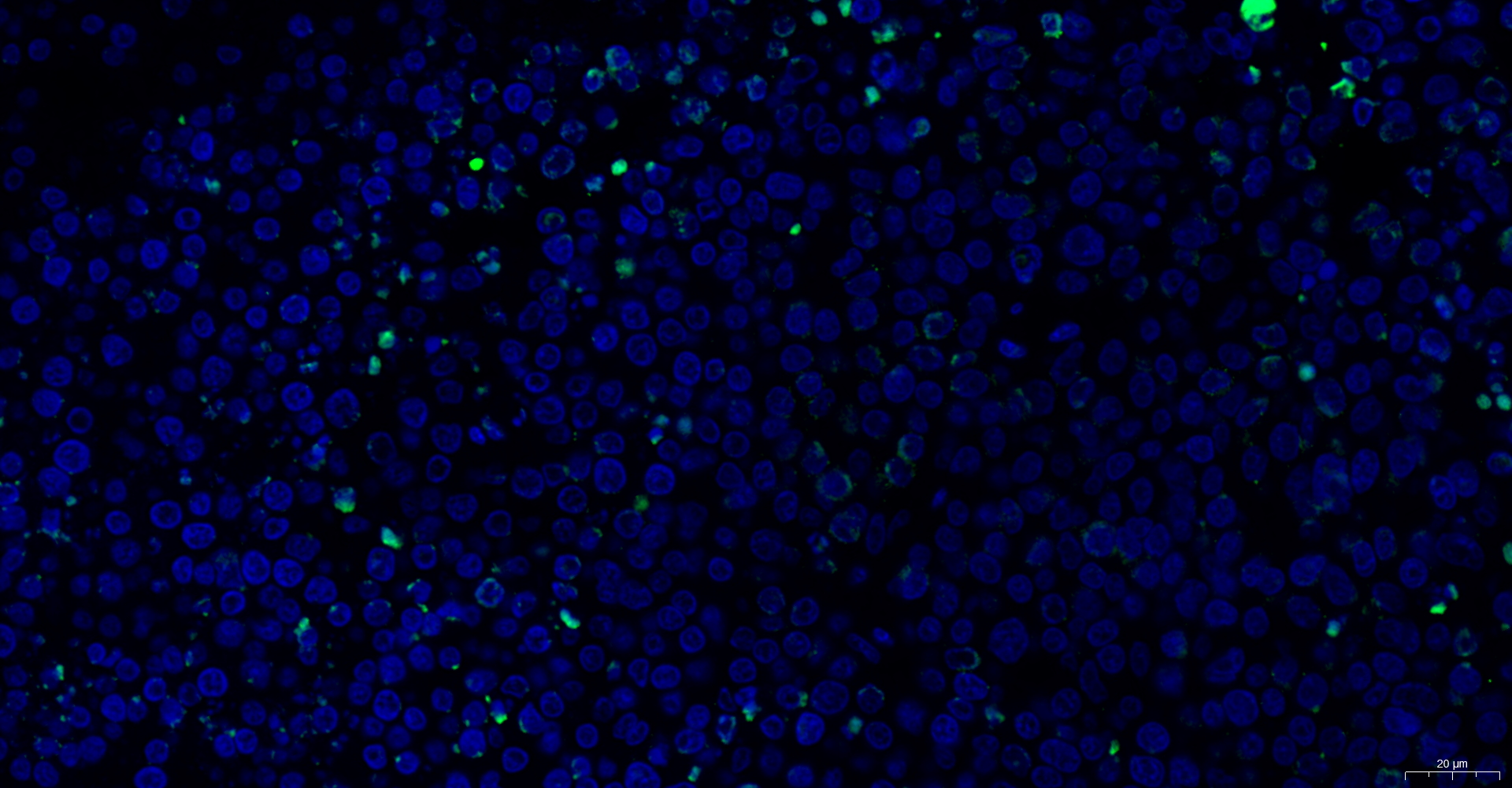

Supplement: Supplementary file 3 [file DataSheet_3.zip › fig5/E2-2-3.jpg]

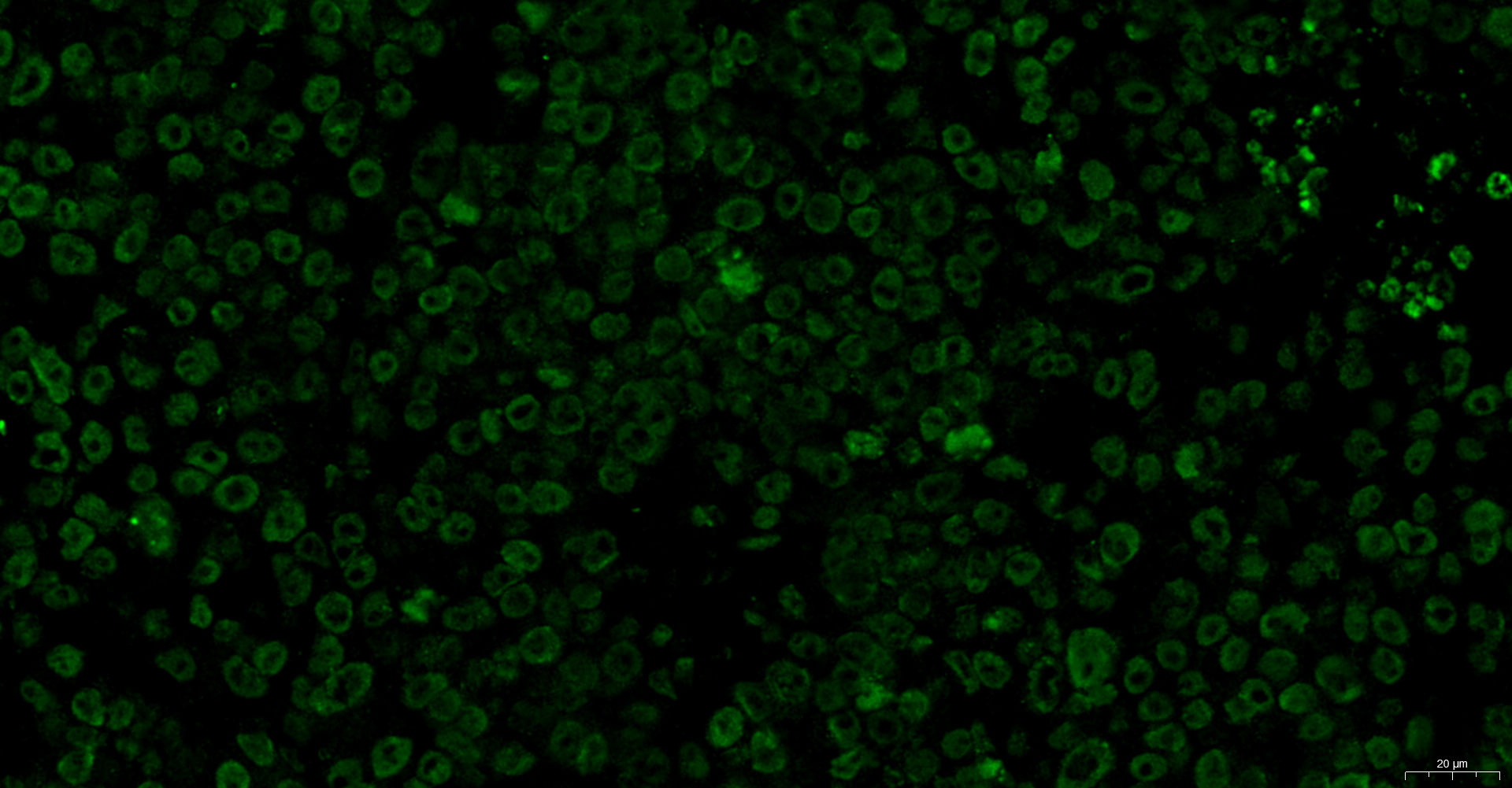

Supplement: Supplementary file 3 [file DataSheet_3.zip › fig5/E2-3-1.jpg]

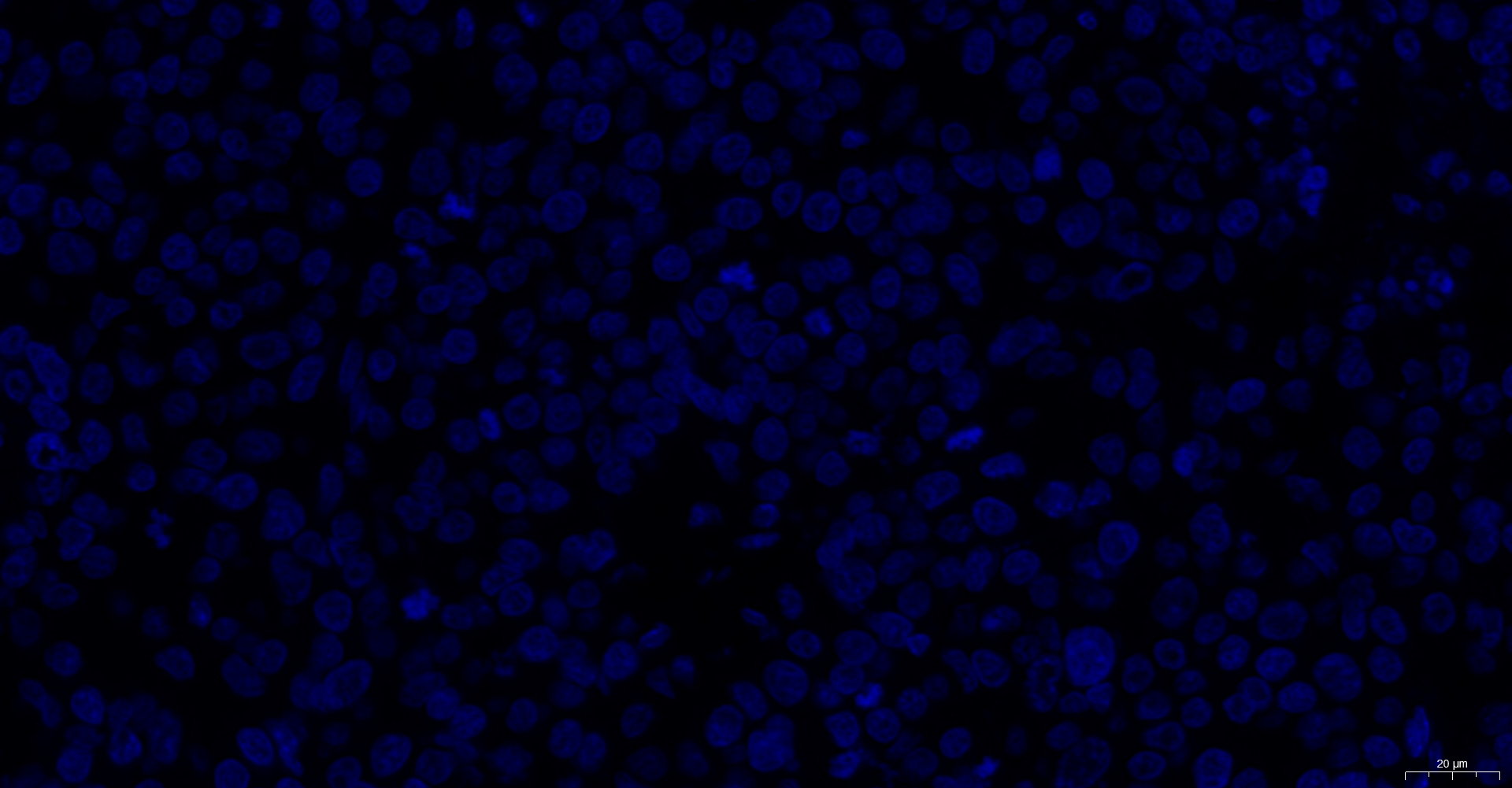

Supplement: Supplementary file 3 [file DataSheet_3.zip › fig5/E2-3-2.jpg]

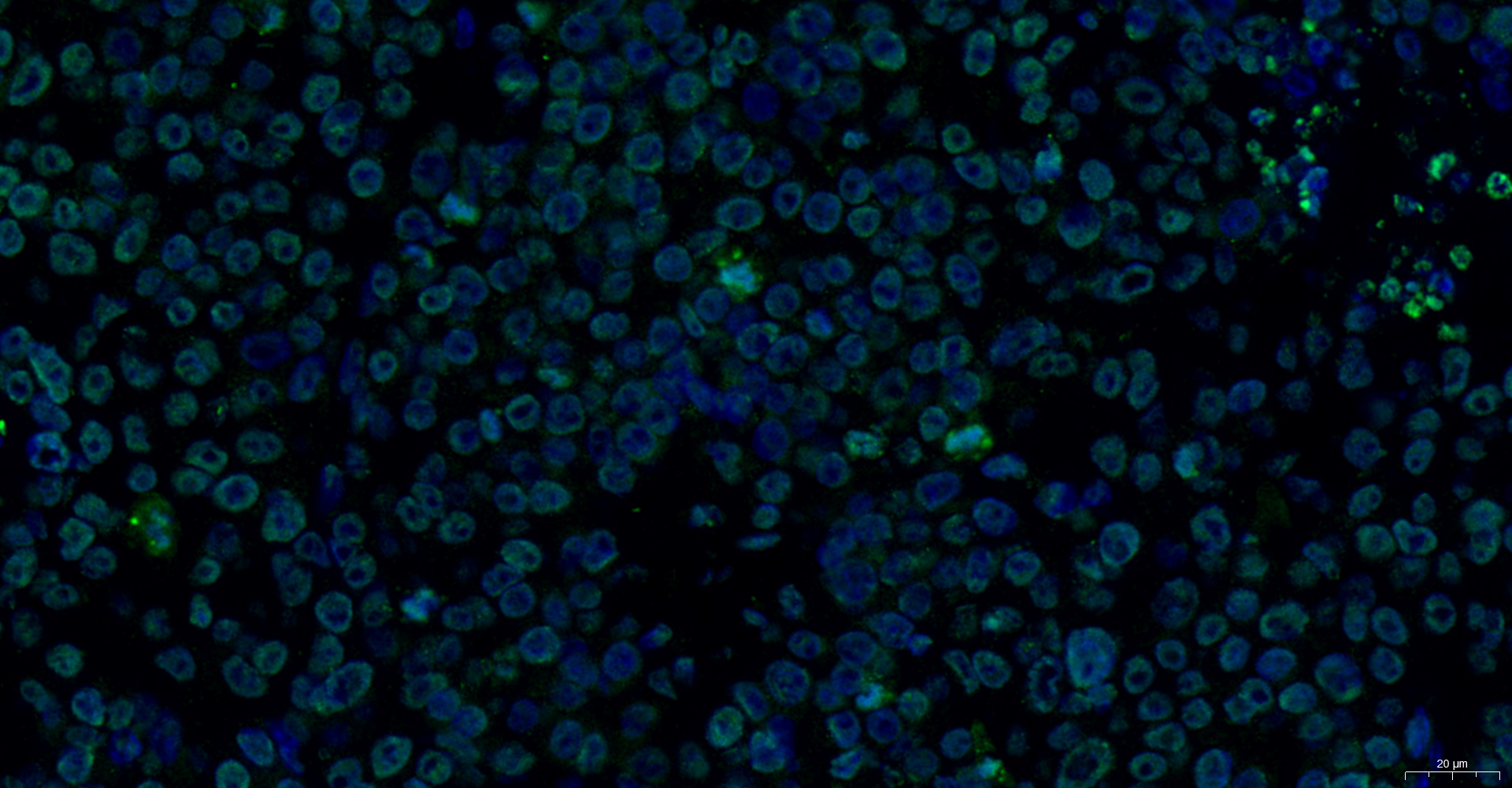

Supplement: Supplementary file 3 [file DataSheet_3.zip › fig5/E2-3-3.jpg]

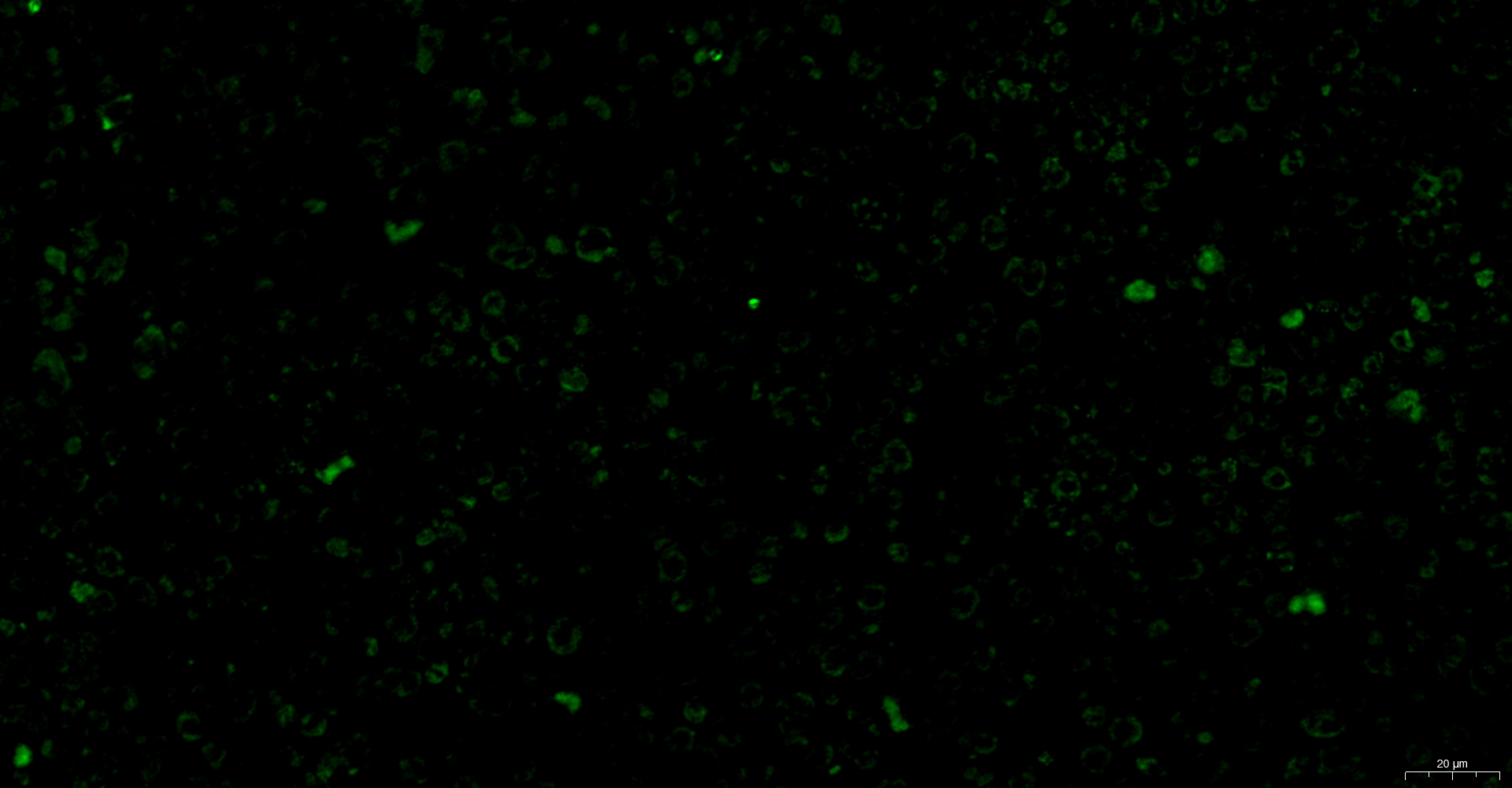

Supplement: Supplementary file 3 [file DataSheet_3.zip › fig5/F3-1-1.jpg]

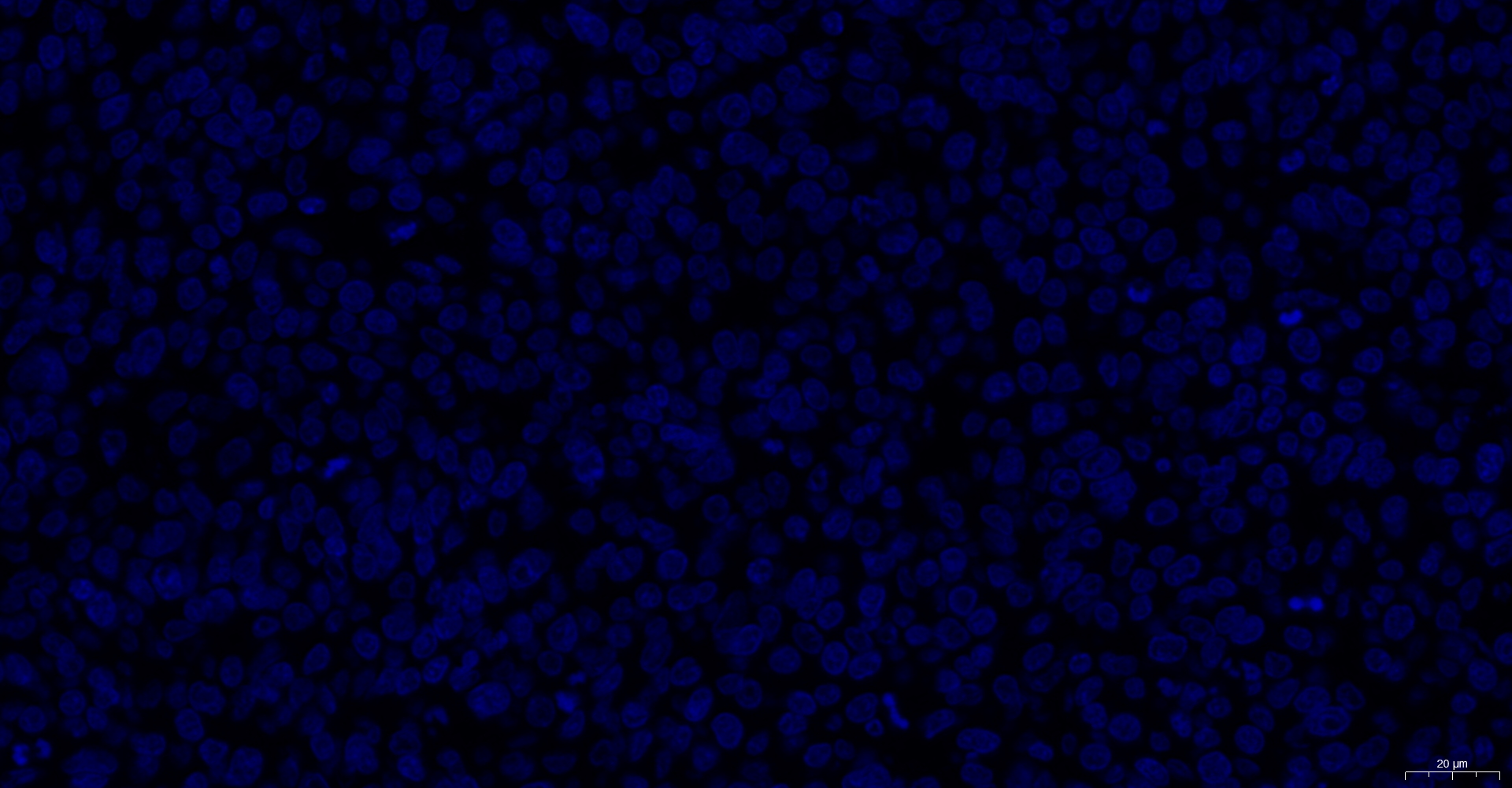

Supplement: Supplementary file 3 [file DataSheet_3.zip › fig5/F3-1-2.jpg]

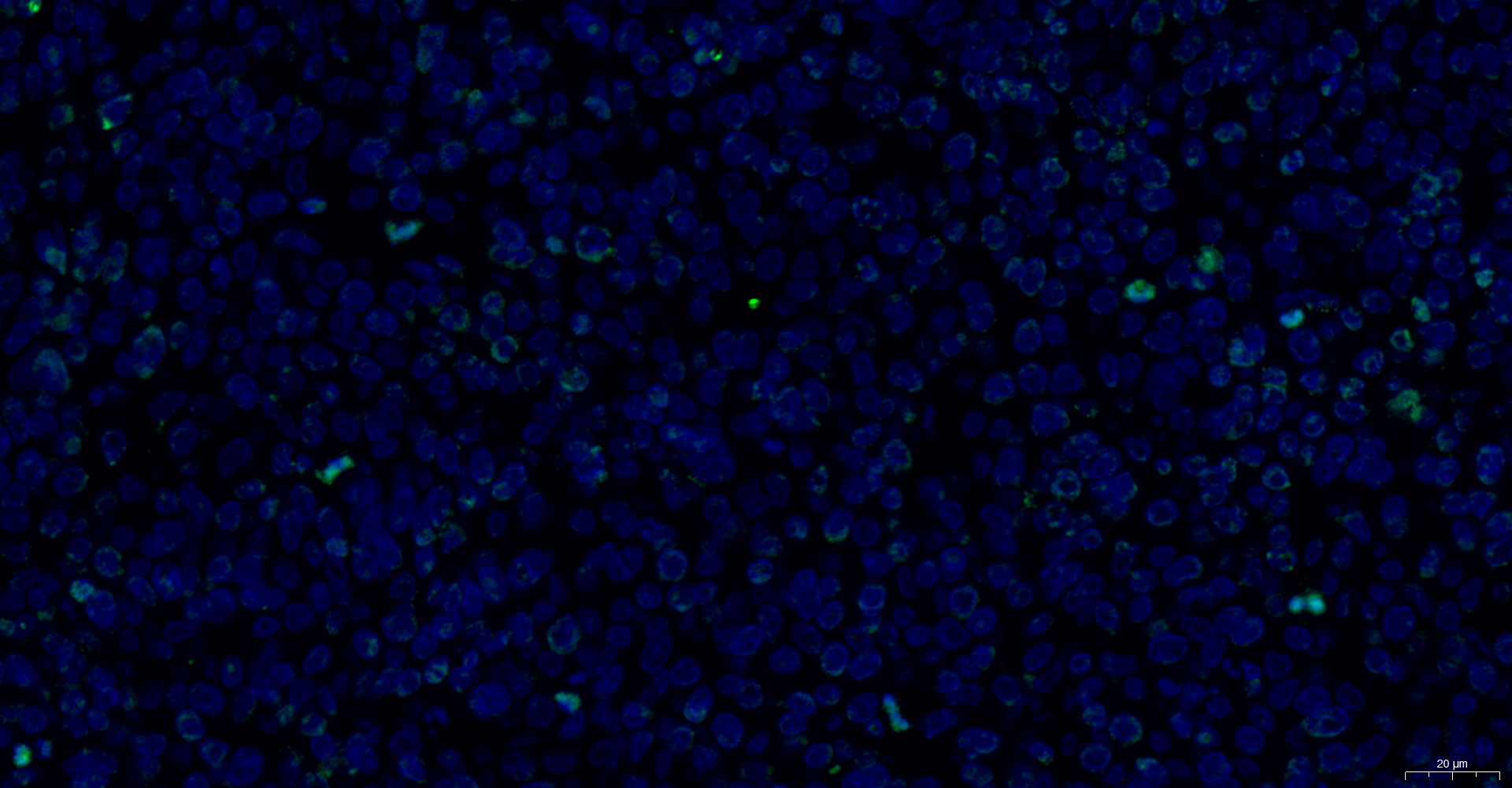

Supplement: Supplementary file 3 [file DataSheet_3.zip › fig5/F3-1-3.jpg]

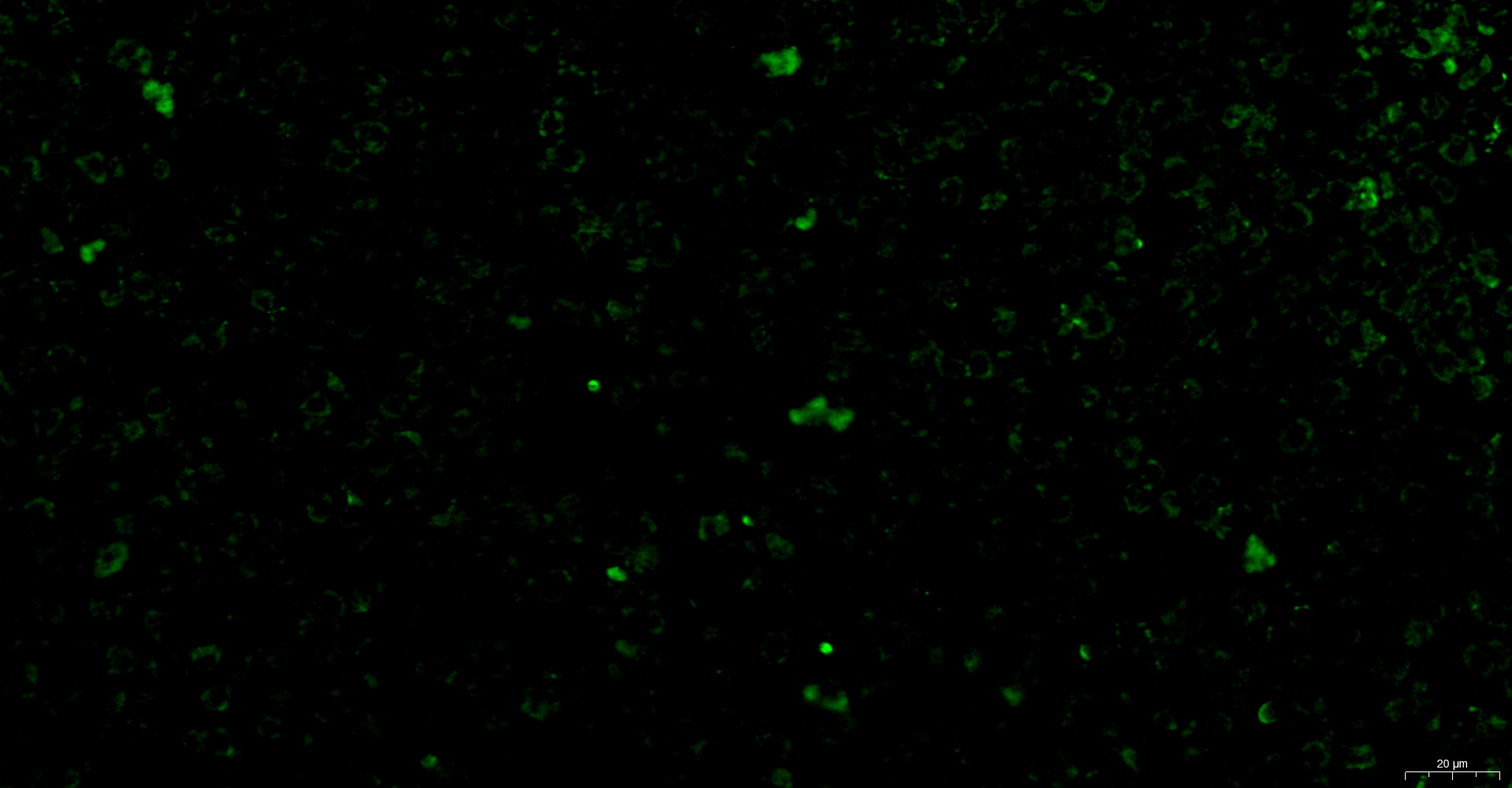

Supplement: Supplementary file 3 [file DataSheet_3.zip › fig5/F3-2-1.jpg]

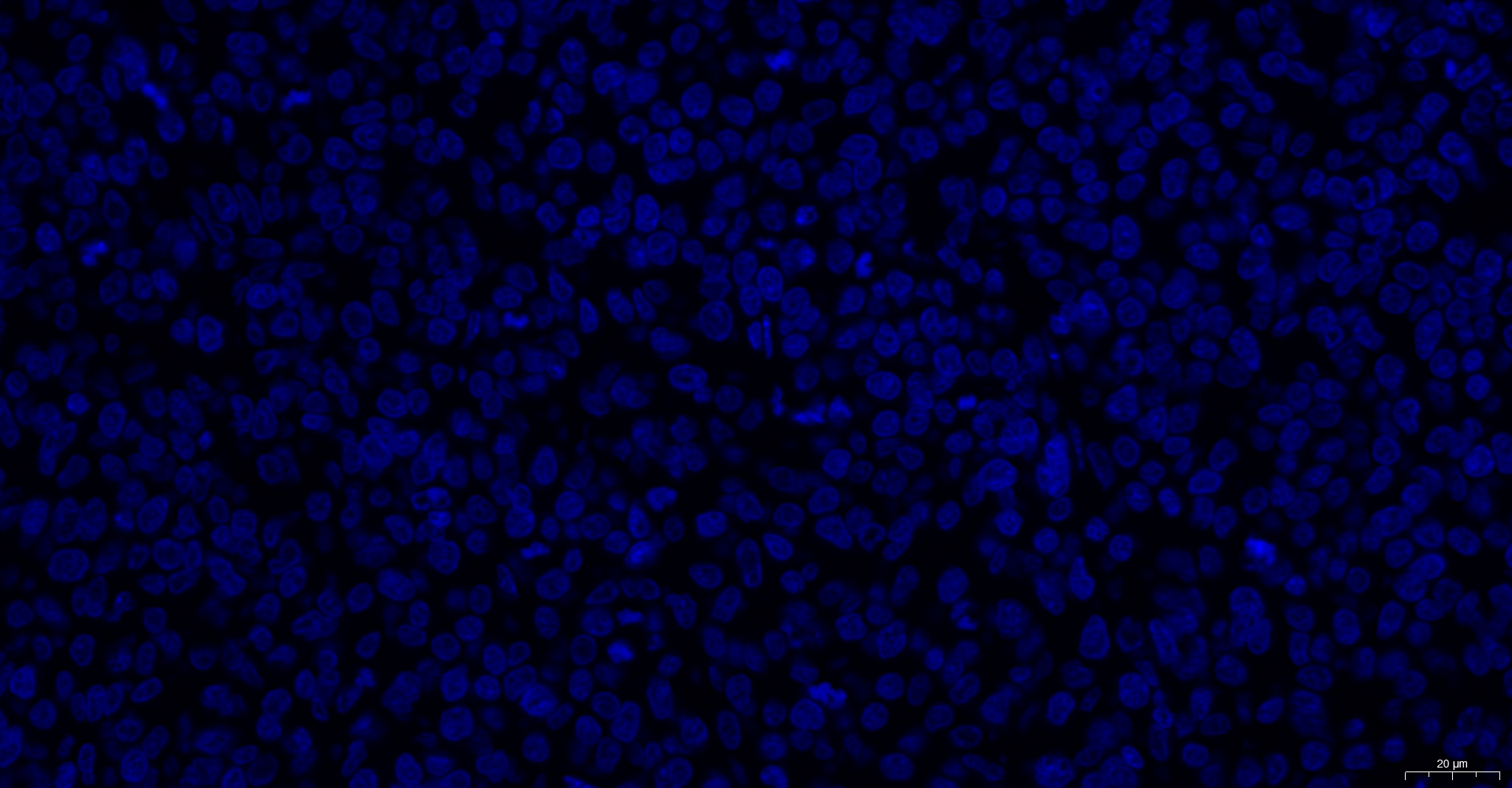

Supplement: Supplementary file 3 [file DataSheet_3.zip › fig5/F3-2-2.jpg]

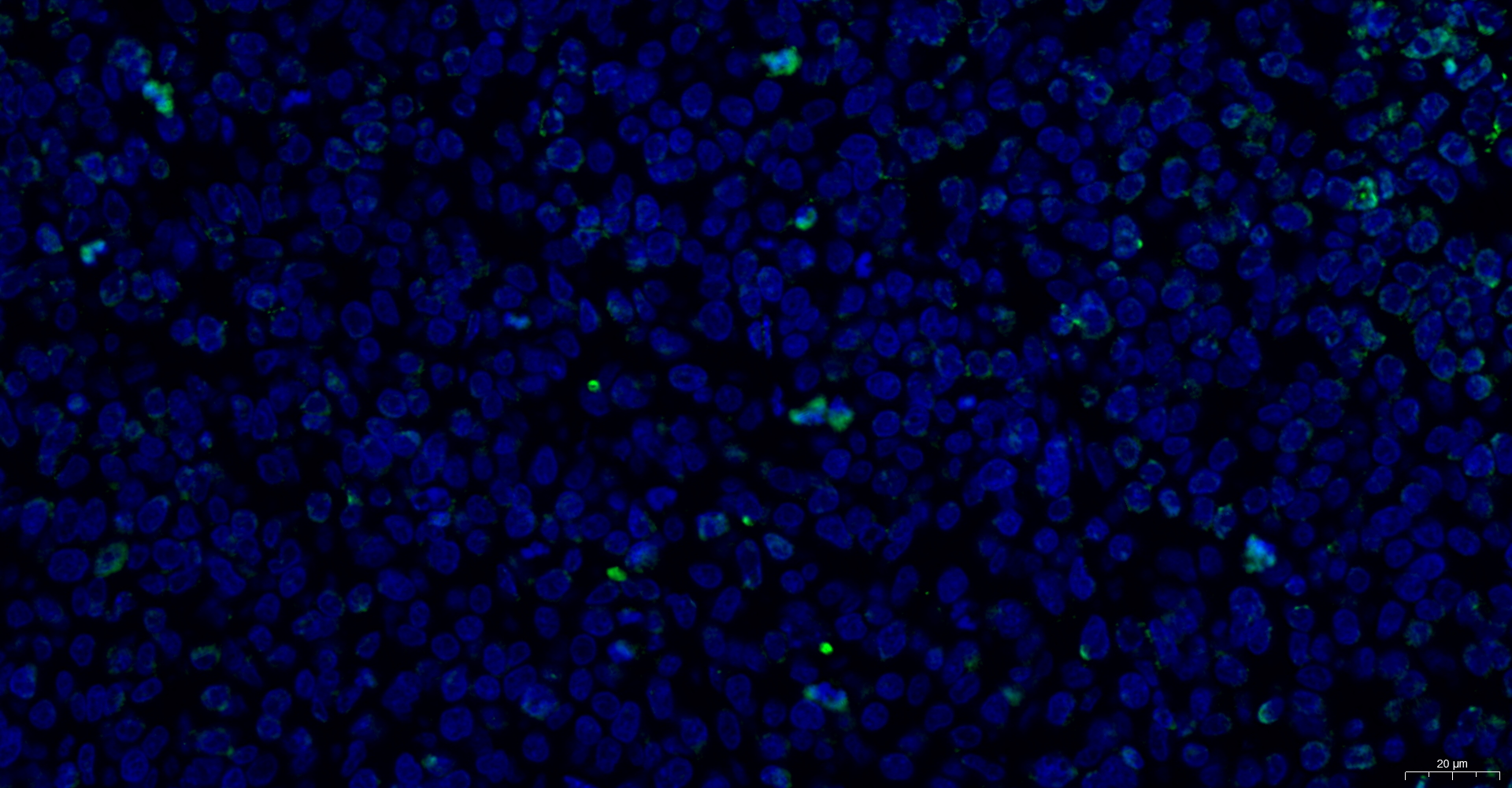

Supplement: Supplementary file 3 [file DataSheet_3.zip › fig5/F3-2-3.jpg]

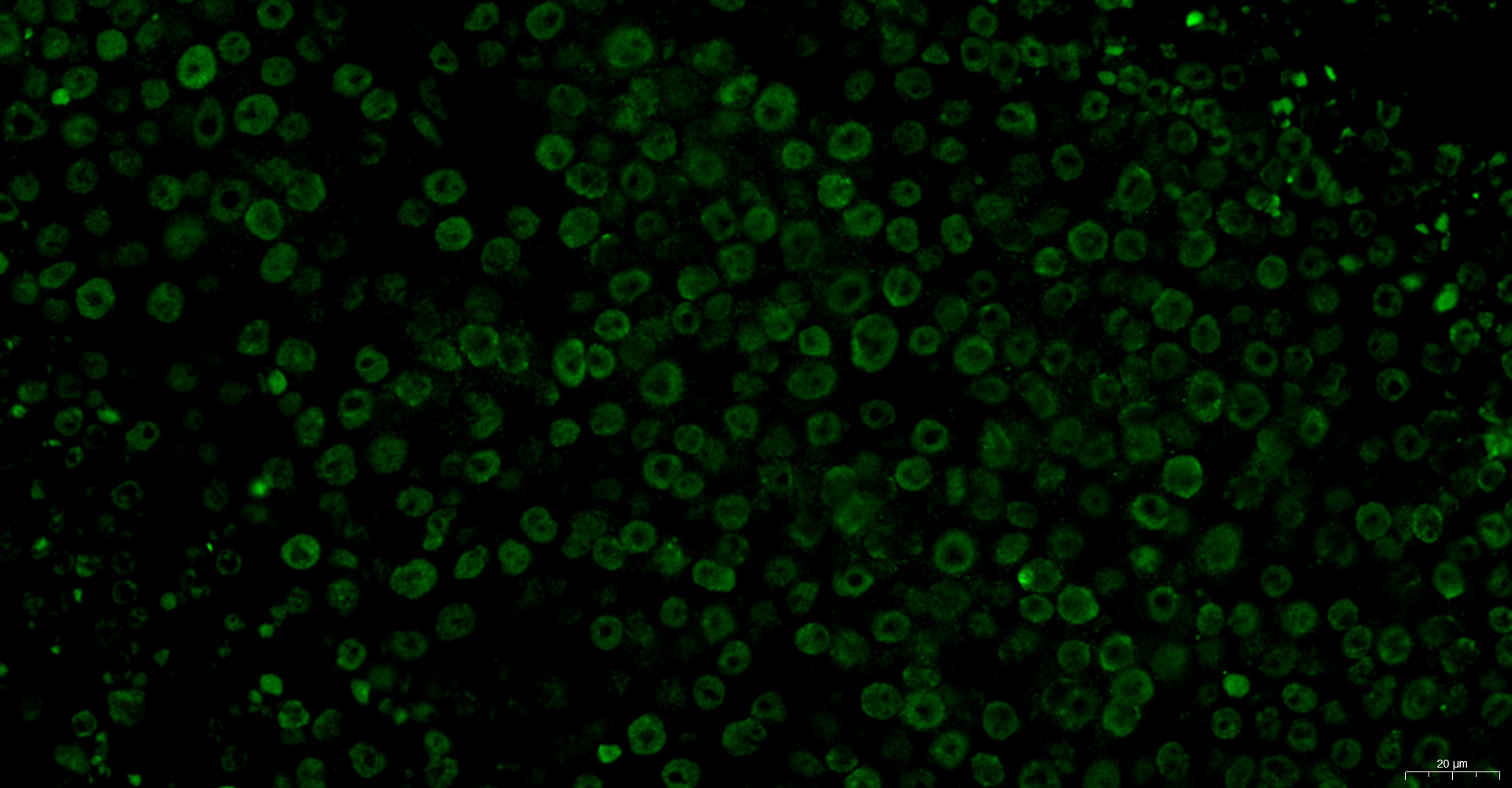

Supplement: Supplementary file 3 [file DataSheet_3.zip › fig5/F3-3-1.jpg]

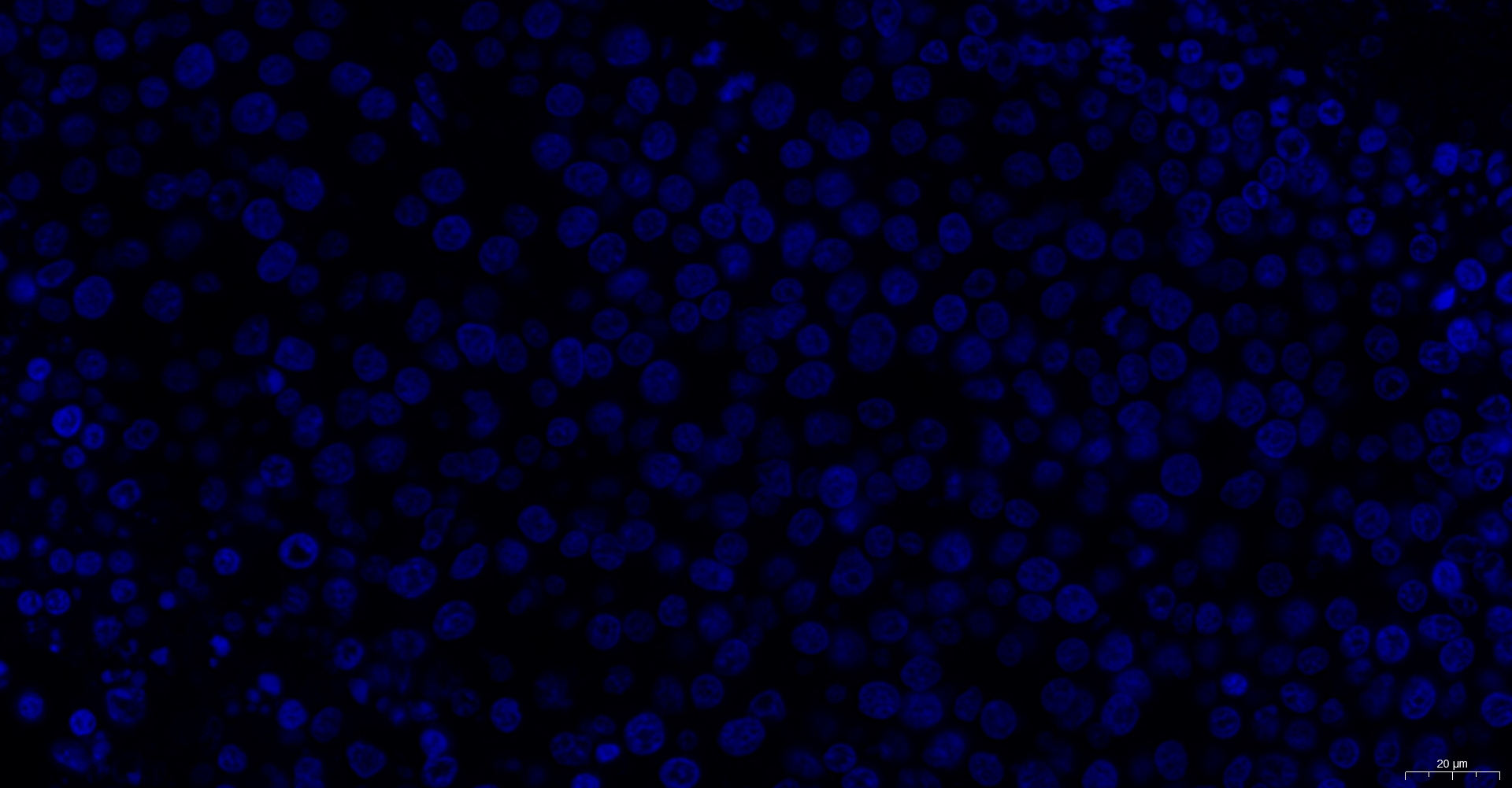

Supplement: Supplementary file 3 [file DataSheet_3.zip › fig5/F3-3-2.jpg]

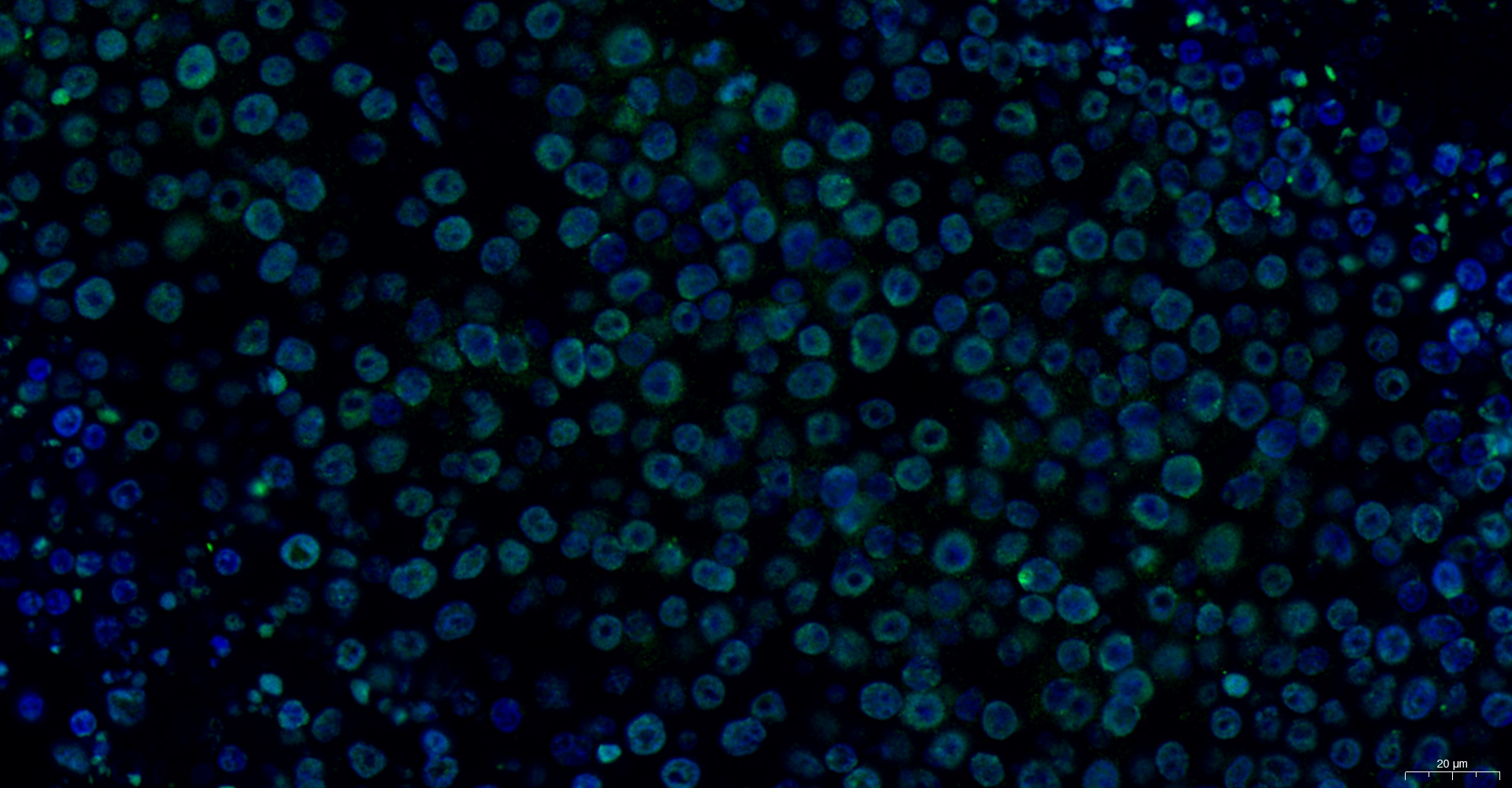

Supplement: Supplementary file 3 [file DataSheet_3.zip › fig5/F3-3-3.jpg]

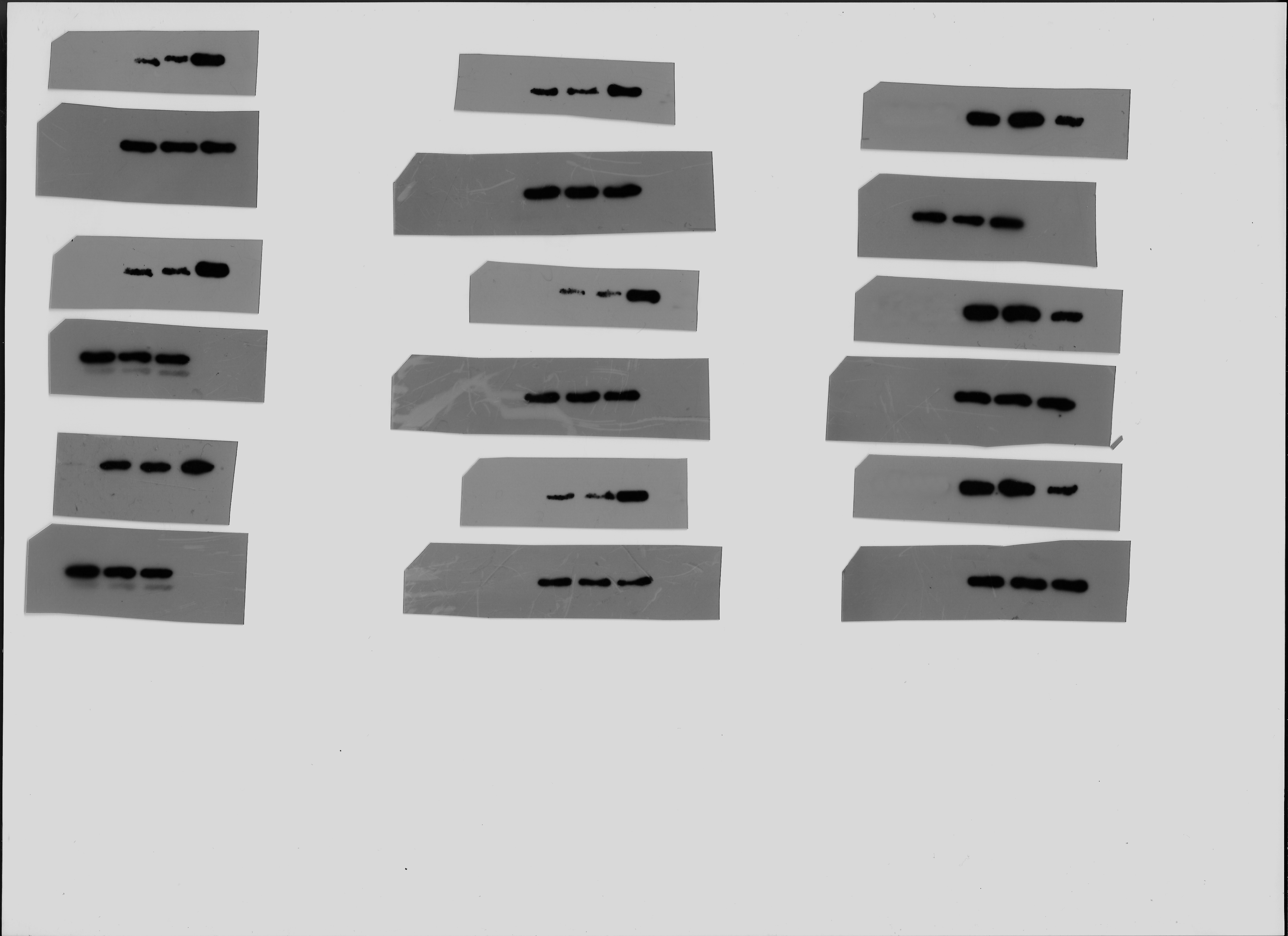

Supplement: Supplementary file 3 [file DataSheet_3.zip › fig5/fig5.jpg]

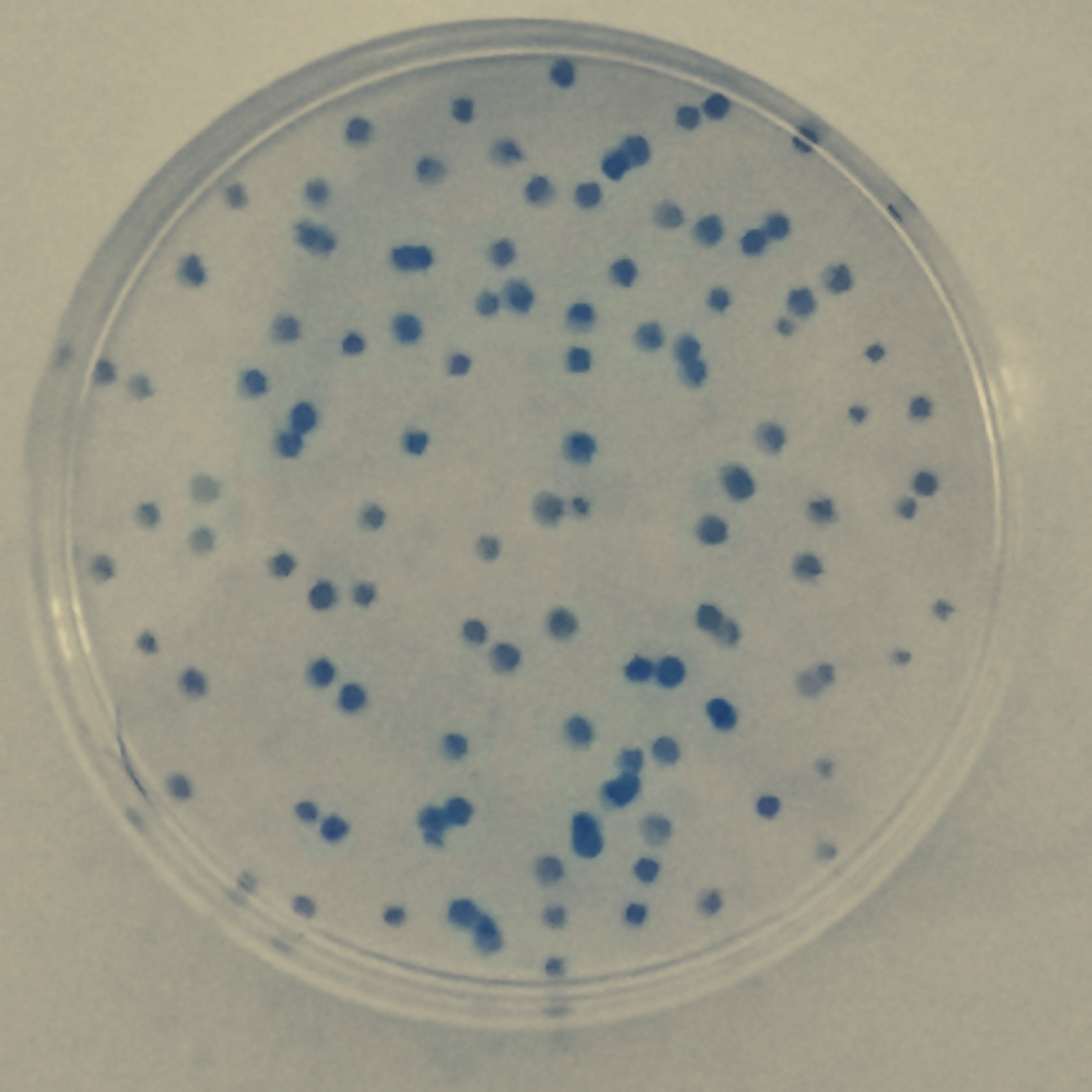

Supplement: Supplementary file 4 [file DataSheet_4.zip › fig8/colony-1.jpg]

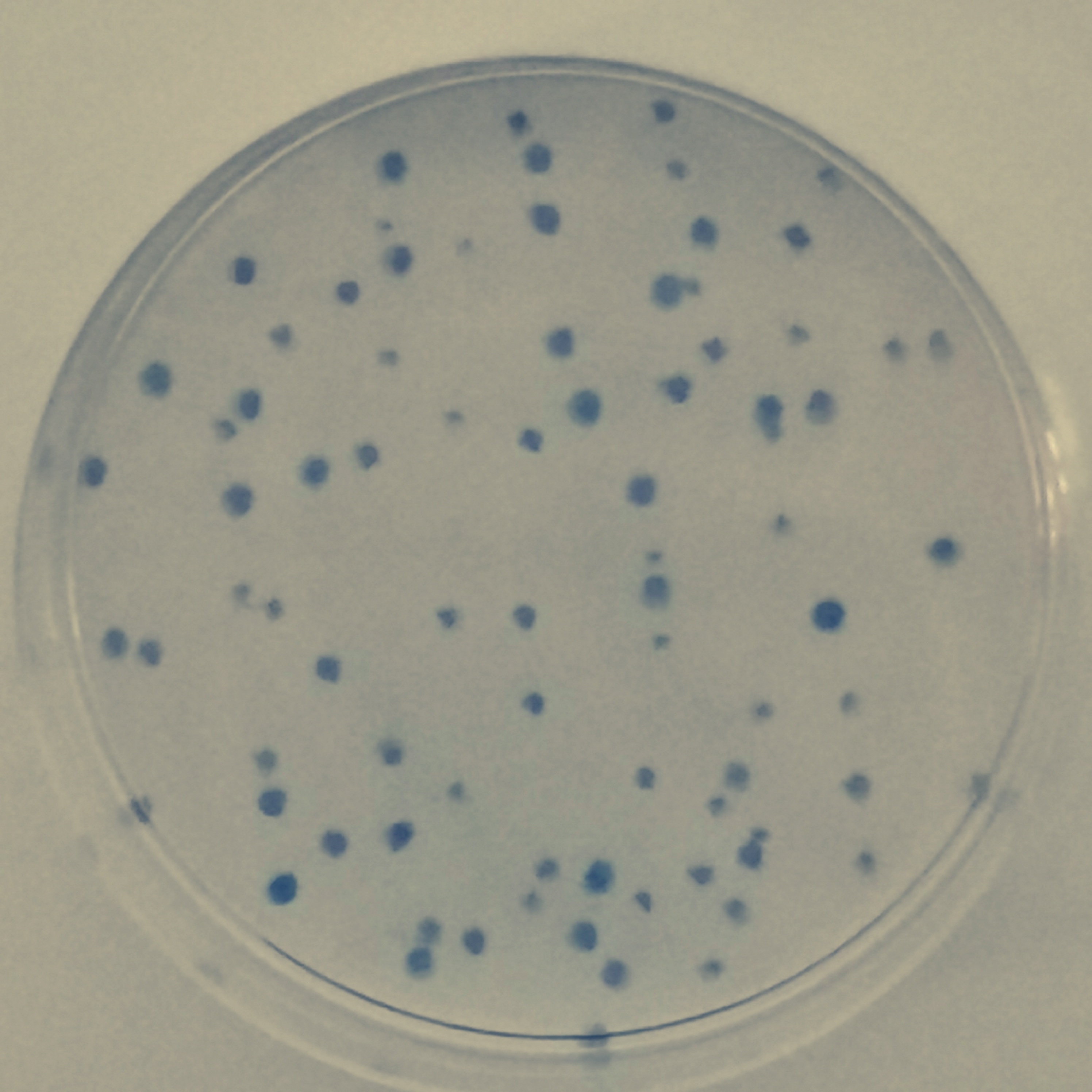

Supplement: Supplementary file 4 [file DataSheet_4.zip › fig8/colony-2.jpg]

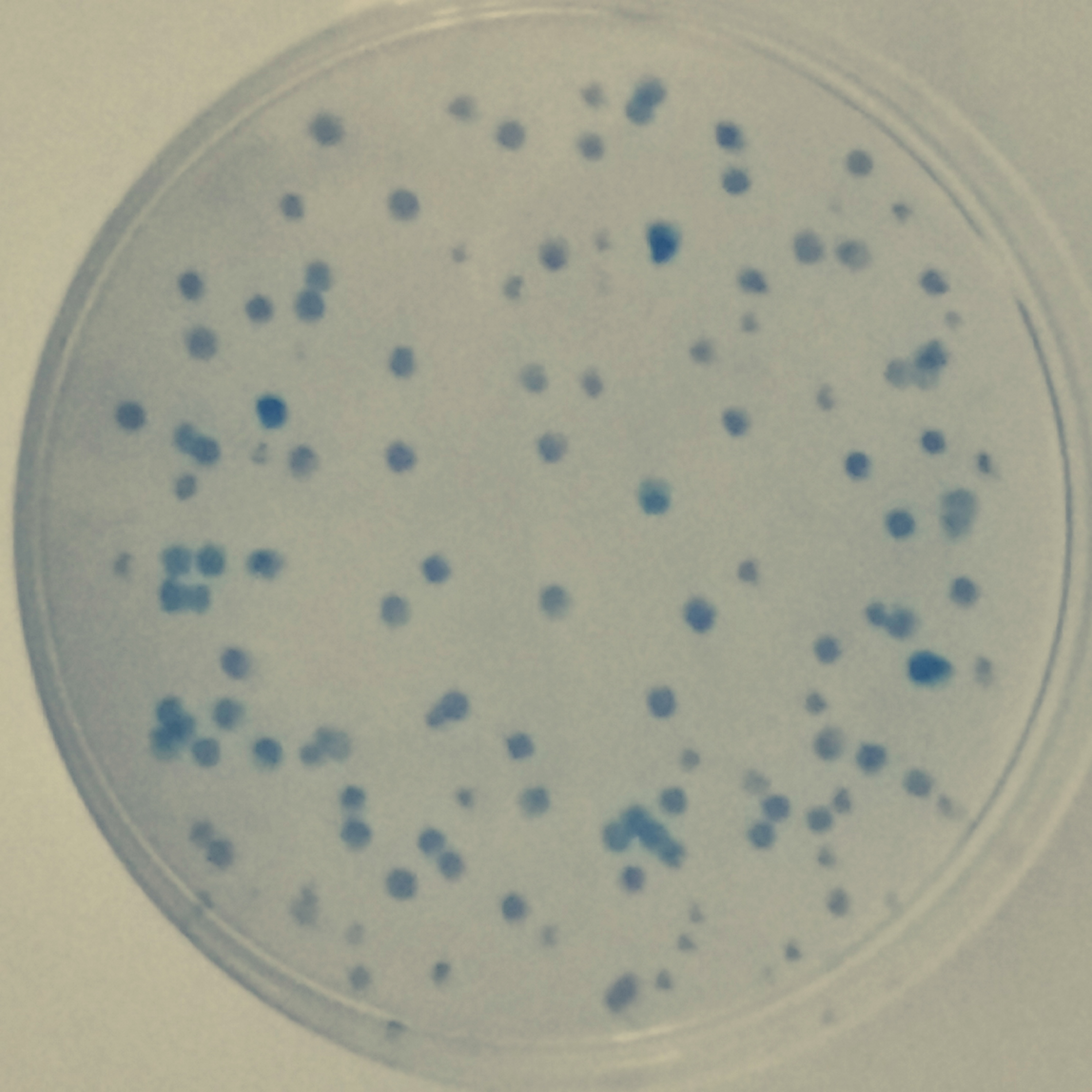

Supplement: Supplementary file 4 [file DataSheet_4.zip › fig8/Colony-3.jpg]

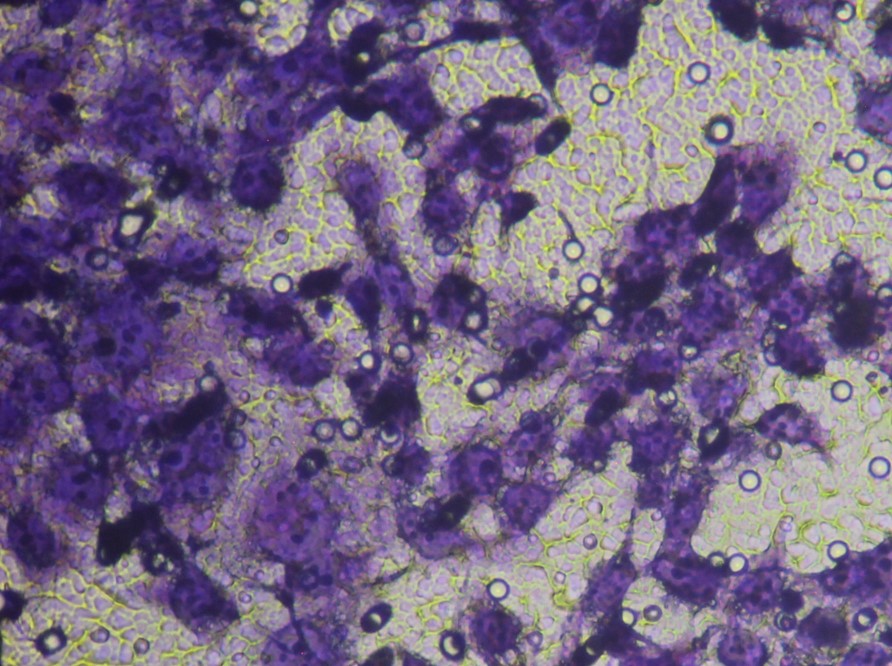

Supplement: Supplementary file 4 [file DataSheet_4.zip › fig8/invasion-1.jpg]

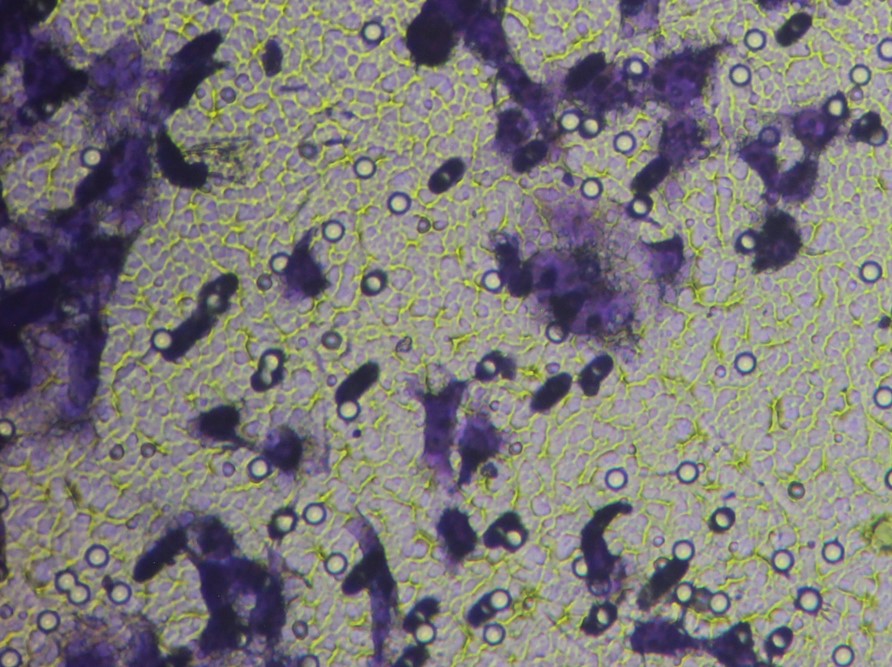

Supplement: Supplementary file 4 [file DataSheet_4.zip › fig8/invasion-2.jpg]

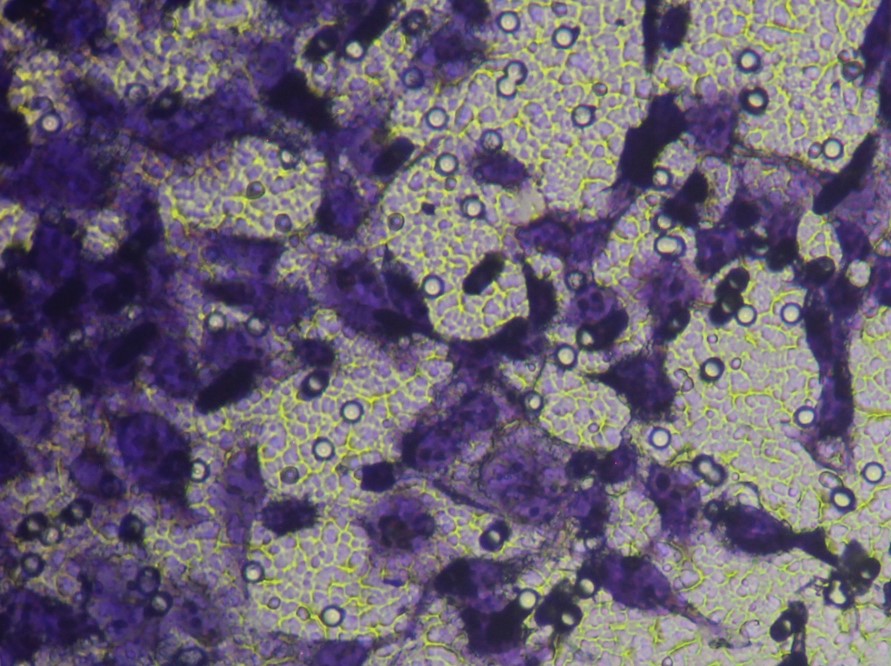

Supplement: Supplementary file 4 [file DataSheet_4.zip › fig8/invasion-3.jpg]

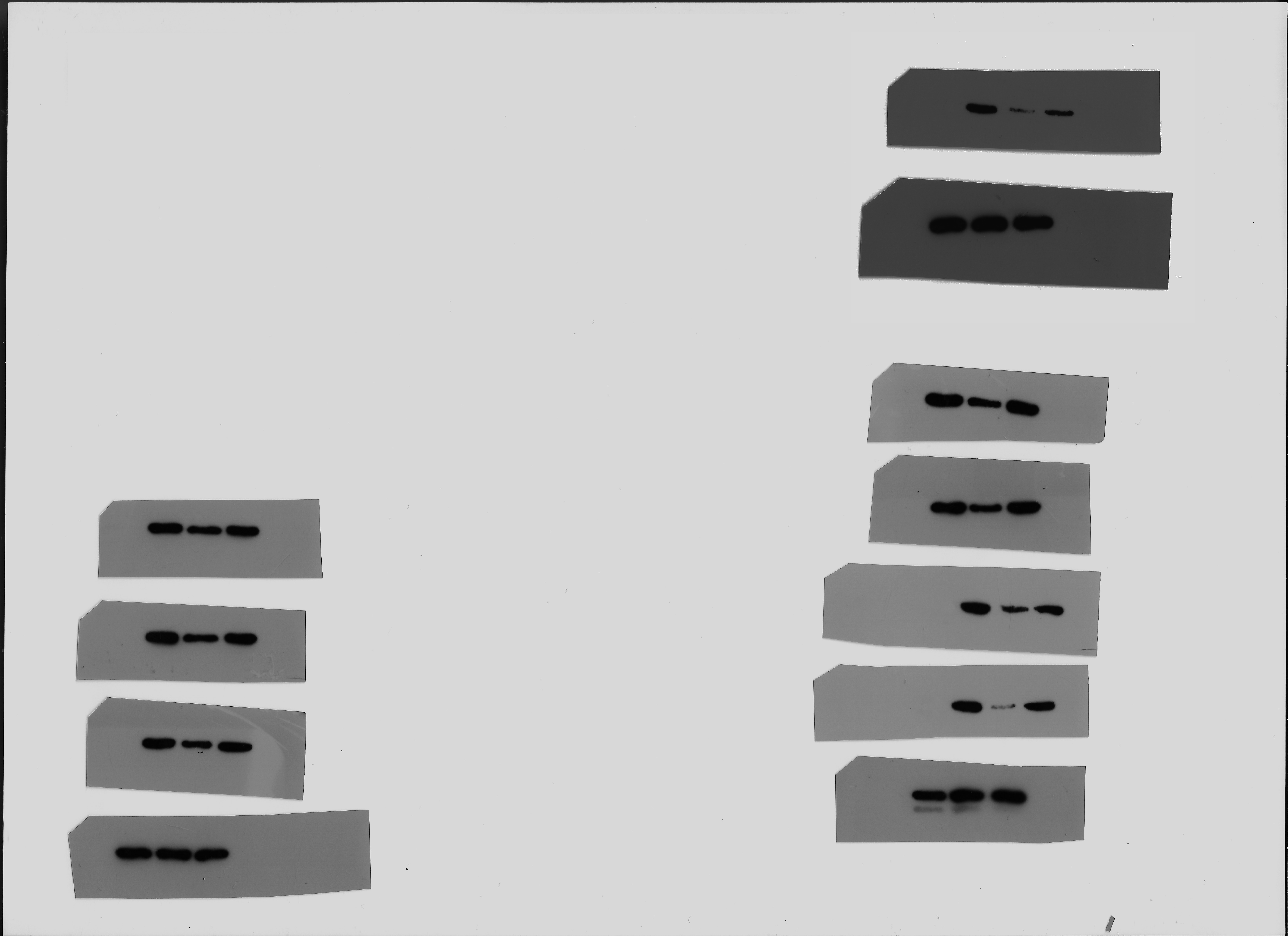

Supplement: Supplementary file 4 [file DataSheet_4.zip › fig8/western blot.jpg]

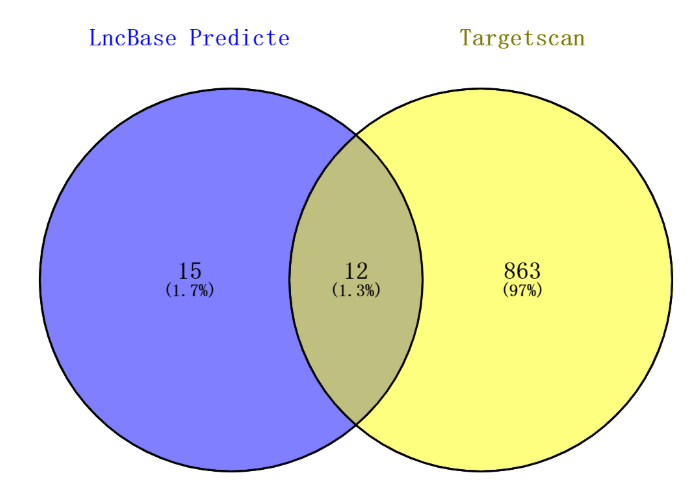

Supplement: Supplementary file 4 [file DataSheet_4.zip › fig7/+¦+n+v1616336729.png]
