# Supplementary material for: A Positive Feedback Loop of lncRNA MIR31HG-miR-361-3p -YY1 Accelerates Colorectal Cancer Progression Through Modulating Proliferation, Angiogenesis, and Glycolysis
Source: Front Oncol. 2021 Aug 17;11:684984. doi: 10.3389/fonc.2021.684984 (PMC8416113; doi:10.3389/fonc.2021.684984)
Supplement: Supplementary file 4 [file DataSheet_4.zip › fig7/--+¿ Microsoft Word +-¦¦.docx]

Position on chromosome:9:21455665-21455689Conserved Species:panTro2,rheMac2,loxAfr3Binding area:

| C | U | G | G | _ | U | _ | _ | C | U | U | C | _ | _ | _ | C | U | U | C | U | _ | _ | _ | _ | _ | _ | _ | _ | _ |
| --- | --- | --- | --- | --- | --- | --- | --- | --- | --- | --- | --- | --- | --- | --- | --- | --- | --- | --- | --- | --- | --- | --- | --- | --- | --- | --- | --- | --- |
| _ | _ | _ | _ | C | _ | G | A | _ | _ | _ | _ | C | A | C | _ | _ | _ | _ | _ | C | C | U | G | G | G | G | G | A |
| _ | _ | _ | _ | \| | _ | \| | \| | _ | _ | _ | _ | \| | \| | \| | _ | _ | _ | _ | _ | \| | \| | \| | \| | \| | \| | \| | \| | \| |
| _ | _ | _ | _ | G | _ | C | U | _ | _ | _ | _ | G | U | G | _ | _ | _ | _ | _ | G | G | A | C | C | C | C | C | U |
| _ | _ | _ | A | _ | U | _ | _ | U | A | _ | _ | _ | _ | _ | U | _ | _ | _ | _ | _ | _ | _ | _ | _ | _ | _ | _ | _ |

| Position 4772-4778 of YY1 3' UTR  [hsa-miR-361-3p](http://www.mirbase.org/cgi-bin/mirna_entry.pl?acc=hsa-miR-361-3p) | 5'  ...UGUCUGCUGCUCUCUCUGGGGGC...                       \|\|\|\|\|\|\|  3'     UUUAGUCUUAGUGUGGACCCCCU |
| --- | --- |

| hsa-miR-361-3p |
| --- |
| hsa-miR-3619-5p |
| hsa-miR-214-3p |
| hsa-miR-342-3p |
| hsa-miR-6780a-3p |
| hsa-miR-5004-5p |
| hsa-miR-761 |
| hsa-miR-646 |
| hsa-miR-3617-3p |
| hsa-miR-2052 |
| hsa-miR-4516 |
| hsa-miR-4434 |
